# The diagnostic value of peroxisome proliferator-activated receptor-γ coactivator-1α in identifying different chronic heart failure phenotypes

**DOI:** 10.3389/fcvm.2022.973705

**Published:** 2022-09-06

**Authors:** Shiwen Zhang, Yufei Zhou, Yanfang Ma, Zhan Li, Yinglong Hou

**Affiliations:** ^1^Department of Cardiology, Shandong Provincial Qianfoshan Hospital, Shandong University, Jinan, China; ^2^Department of Cardiology, The Affiliated Hospital of Xuzhou Medical University, Xuzhou, China; ^3^Department of Cardiology, Shanghai Institute of Cardiovascular Diseases, Zhongshan Hospital and Institutes of Biomedical Sciences, Fudan University, Shanghai, China; ^4^Graduate School, Xuzhou Medical University, Xuzhou, China; ^5^Shandong Medicine and Health Key Laboratory of Cardiac Electrophysiology and Arrhythmia, Department of Cardiology, Shandong Provincial Qianfoshan Hospital, The First Affiliated Hospital of Shandong First Medical University, Jinan, China

**Keywords:** peroxisome proliferator-activated receptor-γ coactlvator-1α, heart failure with reduced ejection fraction, heart failure with mildly reduced ejection fraction, heart failure with preserved ejection fraction, diagnostic

## Abstract

**Background:**

Despite advances in diagnosing and treating chronic heart failure (HF), the underlying mechanisms in different HF phenotypes remain unclear. Mitochondrial energy metabolism is crucial in HF etiology. Our study aimed to explore the value of metabolic-associated biomarker peroxisome proliferator-activated receptor-γ coactivator-1α (PGC1α) in identifying different HF phenotypes.

**Methods:**

A total of 172 participants were enrolled in the Affiliated Hospital of Xuzhou Medical University and were subsequently divided into four groups based on the European Society of Cardiology HF management guideline: the non-HF control (Control, *N* = 46), heart failure with reduced ejection fraction (HFrEF, *N* = 54), heart failure with mildly reduced ejection fraction (HFmrEF, *N* = 22), and heart failure with preserved ejection fraction (HFpEF, *N* = 50) groups. Each participant’s baseline data were recorded, blood samples were taken, and echocardiography was conducted. The level of PGC1α expression was determined using an enzyme-linked immunosorbent assay (ELISA) kit. The receiver operative characteristics (ROC) curve was further established in the four groups to assess the diagnostic value for overall HF and each HF phenotype with the calculation of the area under the curve (AUC) and 95% confidence interval (CI).

**Results:**

PGC1α expression was significantly increased in HF patients (315.0 ± 69.58 nmol/L) compared to non-HF participants (233.3 ± 32.69 nmol/L). Considering different HF phenotypes, PGC1α expression was considerably higher in the HFmrEF group (401.6 ± 45.1 nmol/L)than in the other two phenotypes (299.5 ± 62.27 nmol/L for HFrEF and 293.5 ± 56.37 nmol/L for HFpEF, respectively).Furthermore, the AUCs of PGC1α in overall HF and each HF phenotype were all over 0.8, showing the ideal diagnostic value. Additionally, we provided the cut-off criteria for clinical use, which needs further validation. There was no significant correlation between PGC1α and N-terminal (NT)-prohormone B-type natriuretic peptide (BNP)/blood glucose, suggesting that PGC1α might exert a unique function in HF yet in a different pattern.

**Conclusion:**

We discovered that PGC1α could be used as a potential biomarker for differentiating HF patients from those without HF and for distinguishing HFmrEF from HFrEF and HFpEF.

## Introduction

As one of the most stumbling blocks in the cardiovascular field, chronic heart failure (HF) remains a clinical and public health problem with high morbidity and mortality (1). In recent years, a number of large randomized clinical trials on new HF drugs have been conducted, and several new drugs, such as angiotensin receptor/neprilysin inhibitor and sodium-glucose co-transporter 2 inhibitor, have been approved for clinical use (2). However, there is still a long way to go in terms of HF treatment accuracy due to the complexity of HF phenotypes. The ability to identify different types of HF and elucidate their pathogenesis by more precisely matching the drug plays an important role (3).

The current guidelines propose that HF diagnostic process should be constructed based on these aspects: left ventricular ejection fraction (LVEF) as determined by echocardiography, blood N-terminal pro-B-type natriuretic peptide (NT-proBNP) or BNP level, and clinical signs or symptoms of the patients. HF phenotypes can be divided into three main categories: heart failure with reduced ejection fraction (HFrEF), heart failure with preserved ejection fraction (HFpEF), and heart failure with mildly reduced ejection fraction (HFmrEF) (4). However, the management depending on the exact type among the three is still relatively common. The question of whether the pathogenesis of these three types differs warrants further investigation (5).

Metabolism is crucial in the pathophysiology of cardiovascular diseases, particularly HF (6). The energy required for cardiac contraction and relaxation in the normal heart comes mainly from two pathways. The first is mitochondrial oxidative phosphorylation, which provides approximately 95% of the energy, with the remaining 5% coming primarily from glycolysis (7). The progression of HF is believed to be caused by abnormal substrate utilization, intermediate metabolism, and oxidative stress in patients with HF (8). As a result, we wondered if there are any metabolic-associated markers that can help identify different HF phenotypes.

Adenosine 5′-monophosphate-activated protein kinase (AMPK)/Sirtuin 1 (Sirt1)/peroxisome proliferator-activated receptor-γ coactivator-1α (PGC1α) is a critical pathway in cardiac metabolism. Li et al. (9) have found that enhancing mitochondrial energy metabolism and reducing oxidative stress via regulating AMPK/Sirt1/PGC1α pathway could alleviate myocardial ischemia/reperfusion injury. Another two studies have reported that an exercise-based cardiac rehabilitation program increased Sirt1 activity and stimulated a systemic antioxidant defense in elderly HFpEF patients (10, 11). A series of basic research has also elucidated the beneficial effects of activating Sirt1 in HFpEF models. For instance, Zhang et al. (12) have found that resveratrol could ameliorate cardiac remodeling in a murine model of HFpEF via activating Sirt1. He et al. (13) have identified that canagliflozin could improve myocardial hypertrophy, fibrosis, and cardiac function induced by hypertension in dahl salt-sensitive rats via activating the AMPK/SIRT1/PGC1α pathway, which was highly associated with energy metabolism and oxidative stress. Moreover, Conti et al. (14) have discovered that Sirt1 activity in peripheral blood mononuclear cells could serve as a biomarker of various HF phenotypes. HFpEF patients had lower Sirt1 activity than both HFmrEF and HFrEF patients, but there was no difference when compared to non-HF control. Therefore, as the downstream effector of Sirt1, the value of PGC1α in identifying different HF phenotypes is worth investigating.

## Materials and methods

### Study population

The clinical study was constructed according to the Declaration of Helsinki, and the study protocol was approved by the Ethics Committee of the Affiliated Hospital of Xuzhou Medical University (approval number: XYFY2021-KL116-01; May 25, 2021, Xuzhou, China).

A total of 172 participants were recruited for the study from May 2021 to December 2021 and divided into four groups (control, HFrEF, HFmrEF, and HFpEF) based on the inclusion and exclusion criteria (3) displayed in Table 1.

**TABLE 1:** The inclusion and exclusion criteria for enrolled participants.

Group	Criteria
Inclusion criteria for HFrEF	(1) Typical HF signs and symptoms
	(2) Left ventricular ejection fraction (LVEF) of <40% as determined by echocardiography
	(3) N-terminal pro-brain natriuretic peptide (NT-proBNP) of >125 pg/mL or BNP of >35 pg/mL
Inclusion criteria for HFmrEF	(1) Typical HF signs and symptoms
	(2) LVEF of 40–49%
	(3) NT-proBNP of > 125 pg/mL or BNP of > 35 pg/mL
Inclusion criteria for HFpEF	(1) Typical HF signs and symptoms
	(2) LVEF of > 50%
	(3) NT-proBNP of >125 pg/mL or BNP of >35 pg/mL
	(4) Objective evidence of cardiac structural and/or functional abnormalities consistent with the presence of LV diastolic dysfunction/raised LV filling pressures
Inclusion criteria for non-HF	(1) Be free of HF symptoms and signs
	(2) LVEF of >50%
	(3) Be free of echocardiographic signs of severe diastolic dysfunction
Exclusion criteria for all groups	(1) Malignant tumor
	(2) Severe blood system disease or severe rheumatic immune disease
	(3) Severe mental disease
	(4) Systemic inflammatory response syndrome
	(5) Participants who were unwilling to participate

Control, non-heart failure participants; HFrEF, heart failure with reduced ejection fraction; HFmrEF, heart failure with mildly reduced ejection fraction; HFpEF, heart failure with preserved ejection fraction.

We recorded the participants’ demographic data (age, gender, etc.) and drug use (antiplatelet drug, β-blocker, etc.), measured several items (height, weight, systolic and diastolic blood pressure, etc.), collected data on cardiovascular disease events (coronary heart disease, hypertension, cardiomyopathy, etc.), assessed cardiac function using echocardiography and NYHA classification, and performed blood biochemistry tests.

### Transthoracic echocardiography

Transthoracic echocardiography was performed on all participants using a commercial echocardiographic device (iE33, Philips Healthcare, Best, the Netherlands). Measurements of left ventricular diastolic function were performed according to the guidelines. Trans-mitral early (E) and mitral annular early (e′) diastolic velocity indices were obtained, and E/e′ ratio was calculated to reflect diastolic function.

### Blood sample collection

Whole blood samples were collected, and routine laboratory tests (blood routine examination, C-reactive protein, serum lipid, electrolytes, and hepatorenal function) were performed in the clinical lab of the Affiliated Hospital of Xuzhou Medical University according to the manufacturer’s instruction. A dedicated kit-based NT-proBNP assay (Roche Diagnostics, Basel, Switzerland) was used to measure plasma NT-proBNP levels.

### Enzyme-linked immunosorbent assay

PGC1α expression was quantified using an enzyme-linked immunosorbent assay (ELISA) kit (LP-H06260, Lanpai bio, Shanghai, China). In brief, we added serum, standards, and HRP-labeled detection antibodies to pre-coated PGC1α antibody-coated micropores. For color rendering, 3,3′,5,5′-tetramethylbenzidine (TMB) was used after incubation and thorough washing. TMB is converted to blue by peroxidase and to yellow by acid finally. A positive correlation was observed between color depth and PGC1α in the samples. We measured the absorbance [optic density (OD) value] at 450 nm with a microplate reader and finally calculated the sample concentration.

### Receiver operative characteristics curve establishment and diagnostic value assessment

To evaluate the diagnostic value of PGC1α in identifying HF and each HF phenotype, the Receiver operative characteristics (ROC) curve was established using “pROC” R package (R version 3.6.3). To assess the sensitivity and specificity of the ROC curve, the area under curve (AUC) and 95% confidence interval (CI) were calculated via the “ggplot2” R package.

### Statistical analysis

Data were analyzed using SPSS software Version 20.0 (IBM Corporation, Armonk, NY, United States); GraphPad Prism Version 8.3.0 (GraphPad Software, San Diego, CA, United States). Regarding continuous variables, initial analyses of the normality were conducted using the Kolmogorov-Smirnov (KS) test in conjunction with Q-Q plots (Supplementary Data 1). Differences between multiple groups were evaluated by one-way analysis of variance (ANOVA) with the Bonferroni *post-hoc* test. Data with a normal distribution were represented by mean ± standard deviation (SD), whereas median [interquartile range (IQR)] was used to represent data of a non-normal distribution. The χ^2^ test was used to compare categorical variables that were presented as numbers and percentages. *P* < 0.05 was considered statistically significant.

## Results

### Parameters related to different heart failure phenotypes

**FIGURE 1:**
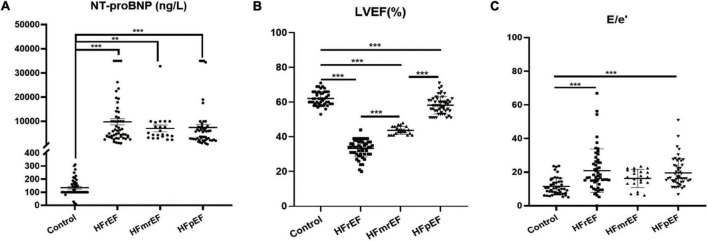
Visualized analysis of important parameters in diagnosing HF phenotypes. **(A)** The comparison regarding the expression of NT-proBNP among Control, HFrEF, HFmrEF and HFpEF groups. **(B,C)** The comparison of LVEF and E/e′ ratio revealed from echocardiography among the four groups. Control, non-heart failure participants; HFrEF, heart failure with reduced ejection fraction; HFmrEF, heart failure with mildly reduced ejection fraction; HFpEF, heart failure with preserved ejection fraction; NT-proBNP, N-terminal pro-B-type natriuretic peptide; LVEF, left ventricular ejection fraction; E/e′, transmitral early (E)/mitral annular early (e′) diastolic velocity ratio. **P < 0.01, ***P < 0.001.

Table 2 and Figure 1 demonstrate important parameters related to the identification of HF phenotypes. NT-proBNP levels (mean ± SD) in the non-HF, HFrEF, HFmrEF, and HFpEF groups were 134.2 ± 66.13, 9809 ± 9406, 7073 ± 6360, and 7489 ± 8975 ng/L, respectively, showing a significant difference between the HF groups and the non-HF control group. Due to the large variation in NT-proBNP level, we further used median and quartile to describe NT-proBNP level. Similarly, the median expression level in the control group was lower than in the HF groups. Additionally, we discovered that the HFrEF group had a higher level of NT-proBNP expression than the other two HF groups.

**TABLE 2:** Parameters in diagnosing different heart failure phenotypes.

Characteristics	Control	HFrEF	HFmrEF	HFpEF	*P*-value
	*N* = 46	*N* = 54	*N* = 22	*N* = 50	
NT-proBNP, ng/L	134.2 ± 66.13	9,809 ± 94,06	7,073 ± 6,360	7,489 ± 8,975	<0.0001
Median (Q1, Q3)	100 (100, 170.3)	6,010 (3,501, 12,375)	4,885 (3,606, 9,674)	4,622 (2,355, 78,08)	
**Echocardiography**					
LVEF,%	62.13 ± 4.064	33.33 ± 4.979	43.73 ± 2.208	58.24 ± 5.057	<0.0001
E/e′	11.43 ± 5.084	20.84 ± 12.92	16.25 ± 5.437	19.6 ± 8.486	<0.0001

Continuous variables were presented as mean ± SD or median (Q1, Q3). P < 0.05 was considered statistically significant.

Control, non-heart failure participants; HFrEF, heart failure with reduced ejection fraction; HFmrEF, heart failure with mildly reduced ejection fraction; HFpEF, heart failure with preserved ejection fraction; NT-proBNP, N-terminal pro–B-type natriuretic peptide; LVEF, left ventricular ejection fraction; E/e′, transmitral early (E)/mitral annular early (e′) diastolic velocity ratio.

Furthermore, we assessed all participants by echocardiography. We mainly focused on LVEF and E/e′ ratio to assess cardiac systolic and diastolic functions. The results showed that LVEF (mean ± SD) in the non-HF, HFrEF, HFmrEF, and HFpEF groups were 62.13 ± 4.064, 33.33 ± 4.979, 43.73 ± 2.208, and 58.24 ± 5.057, respectively, which was consistent with the guidelines. Also, the E/e′ ratio for the four groups was 11.43 ± 5.084, 20.84 ± 12.92, 16.25 ± 5.437, and 19.6 ± 8.486, respectively, indicating that the diastolic cardiac function was impaired in HF.

### Baseline characteristics of the study population

As shown in Table 3, the study population comprised 172 participants divided into four groups (*N* = 46 for the non-HF group, *N* = 54 for the HFrEF group, *N* = 22 for the HFmrEF group, and *N* = 50 for the HFpEF group). There was no difference in terms of gender or height. Intriguingly, several items showed significant differences among the four groups. HF patients had a higher coronary heart disease rate and used more angiotensin-converting enzyme inhibitor (ACEI)/angiotensin receptor blocker (ARB) and diuretic drugs. Regarding blood biochemistry tests, HF patients had lower hemoglobin, higher creatinine, and higher C-reactive protein levels, indicating that HF patients are prone to inflammation when compared with non-HF controls.

**TABLE 3:** Main characteristics of the study population.

Characteristics	Control	HFrEF	HFmrEF	HFpEF	*P-*value
	*N* = 46	*N* = 54	*N* = 22	*N* = 50	
Age, years	62 (54, 68)	66 (58, 74)	72 (65, 81)	76 (69, 80)	<0.001
Gender, *n*(%)					0.443
Male	24 (52.17)	33 (61.11)	13 (50.09)	23 (46.00)	
Female	22 (47.83)	21 (38.89)	9 (40.91)	27 (54.00)	
Height, cm	161.5 (160, 168)	165 (160, 170)	165 (160, 175)	164 (160, 170)	0.281
Weight, kg	70 (60, 76)	62 (55, 71)	69 (60, 85)	65 (60, 75)	0.122
BMI, kg/m^2^	25.61 ± 3.52	23.37 ± 3.47	27.13 ± 6.57	25.14 ± 4.51	0.003
HR, bpm	70 (63, 78)	84 (70, 100)	79 (68, 93)	73 (61, 89)	<0.001
SBP, mmHg	137 ± 17	121 ± 23.40	127 ± 20	133 ± 21	0.001
DBP, mmHg	83 (75, 90)	76 (65, 81)	75 (64, 85)	77 (63, 83)	0.015
Smoking, *n*(%)	13 (28.26)	21 (38.89)	11 (50.00)	17 (34.00)	0.341
NYHA, *n*(%)					0.001
I/II	0 (0)	13 (24.07)	6 (27.27)	31 (62.00)	
III/IV	0 (0)	41 (75.93)	16 (72.73)	19 (38.00)	
CHD, *n*(%)	24 (52.17)	31 (57.41)	17 (77.28)	38 (76.00)	0.034
MI, *n*(%)	0 (0)	17 (31.48)	9 (40.91)	20 (40.00)	0.595
Hypertension, *n*(%)	22 (47.83)	19 (35.19)	12 (54.55)	31 (62.00)	0.050
Cardiomyopathy, *n*(%)	0 (0)	14 (25.93)	1 (4.55)	4 (8.00)	0.012
Hyperlipidemia, *n*(%)	8 (17.39)	3 (5.56)	1 (4.55)	5 (10.00)	0.190
AF, *n*(%)	5 (10.87)	13 (24.07)	7 (31.82)	12 (24.00)	0.182
PAH, *n*(%)	0 (0)	5 (9.26)	2 (9.09)	6 (10.00)	0.987
DM, *n*(%)	11 (23.91)	18 (33.33)	10 (45.45)	13 (26.00)	0.266
**Drug Use, *n*(%)**					
Aspirin	27 (58.70)	22 (40.74)	9 (40.91)	26 (52.00)	0.265
Aspirin + clopidogrel	8 (17.39)	12 (22.22)	8 (36.36)	19 (38.00)	0.079
Statins	36 (78.26)	32 (59.26)	15 (68.18)	38 (76)	0.146
β-blocker	25 (54.35)	44 (81.48)	20 (90.91)	27 (54)	<0.001
ACEI/ARB	9 (19.57)	44 (81.48)	13 (59.09)	25 (50.00)	<0.001
Diuretics	3 (6.52)	49 (90.74)	19 (86.36)	30 (60.00)	<0.001
**Blood biochemistry**					
Hemoglobin, g/L	139 ± 16	131 ± 24	121 ± 22	119 ± 24	<0.001
CRP, mg/L	0.7 (0.5,1.9)	4.3 (1.5, 14.3)	9.5 (0.6, 36.8)	4.5 (1.1,27.0)	<0.001
Creatinine, μmol/L	60 (52, 67)	72 (58, 92)	82 (58, 116)	74 (60, 102)	0.001
Blood glucose, mmol/L	5.1 (4.5, 6.2)	5.4 (4.7, 7.3)	5.9 (5.5, 8.0)	5.4 (4.9, 6.3)	0.015
HbA1c,%	6.1 ± 0.9	6.9 ± 1.6	6.7 ± 1.1	6.4 ± 1.2	0.108
TC, mmol/L	4.18 (3.58, 3.86)	3.83 (3.18, 4.51)	3.78 (3.29, 4.48)	3.89 (3.20, 4.47)	0.075
TG, mmol/L	1.10 (0.88, 1.71)	1.18 (0.84, 1.48)	1.09 (0.79, 1.67)	1.24 (0.85, 1.67)	0.353
HDL, mmol/L	1.09 (0.89, 1.37)	0.91 (0.76, 1.61)	0.93 (0.75, 1.09)	0.99 (0.74, 1.24)	0.111
LDL, mmol/L	2.48 ± 0.72	2.40 ± 0.74	2.33 ± 0.79	2.23 ± 0.83	0.503
Na^+^, mmol/L	140.4 (138.4, 142.5)	140.6 (138.6, 142.6)	139.8 (137.9, 141.3)	139.7 (137, 141.9)	0.336

Continuous variables were presented as mean ± SD or median (IQR), and categorical variables were presented as n (%). P < 0.05 was considered statistically significant.

Control, non-heart failure participants; HFrEF, heart failure with reduced ejection fraction; HFmrEF, heart failure with mildly reduced ejection fraction; HFpEF, heart failure with preserved ejection fraction; BMI, body mass index; HR, heart rate; SBP, systolic blood pressure; DBP, diastolic blood pressure; NYHA, New York Heart Association; CHD, coronary heart disease; MI, myocardial infarction; AF, atrial fibrillation; PAH, pulmonary arterial hypertension; DM, diabetes mellitus; ACEI, angiotensin-converting enzyme inhibitors; ARB, angiotensin receptor blockers; CRP, C-reactive protein; HbA1c, glycated hemoglobin; TC, total cholesterol; TG, total triglyceride; HDL, high-density lipoprotein; LDL, low-density lipoprotein.

### Peroxisome proliferator-activated receptor-γ coactivator-1α expression

**FIGURE 2:**
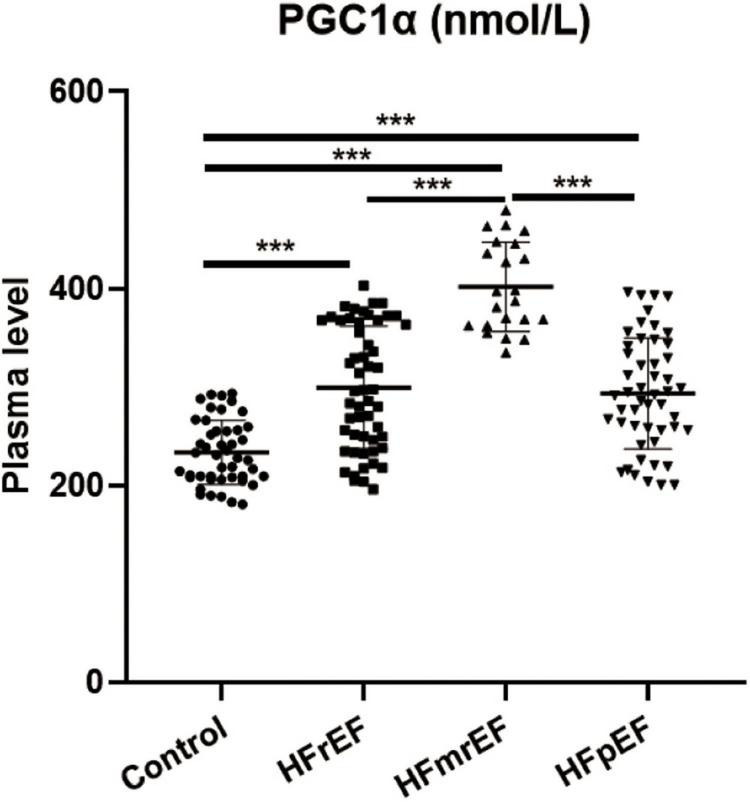
PGC1α expression in different HF phenotypes. PGC1α, peroxisome proliferator-activated receptor-γ coactivator-1α; Control, non-heart failure participants; HFrEF, heart failure with reduced ejection fraction; HFmrEF, heart failure with mildly reduced ejection fraction; HFpEF, heart failure with preserved ejection fraction. ***P < 0.001.

Figure 2 shows the expression level of PGC1α in non-HF controls and three HF phenotypes. The relative plasma level in the four groups (control, HFrEF, HFmrEF, and HFpEF groups, mean ± SD) was 233.3 ± 32.69, 299.5 ± 62.27, 401.6 ± 45.10, and 293.5 ± 56.37 nmol/L, respectively. We revealed that compared with non-HF controls, PGC1α expression was remarkably upregulated in both three HF phenotypes. Additionally, HFmrEF patients had a higher PGC1α level than the other two HF phenotypes.

### Diagnostic value of expression of peroxisome proliferator-activated receptor-γ coactivator-1α

**FIGURE 3:**
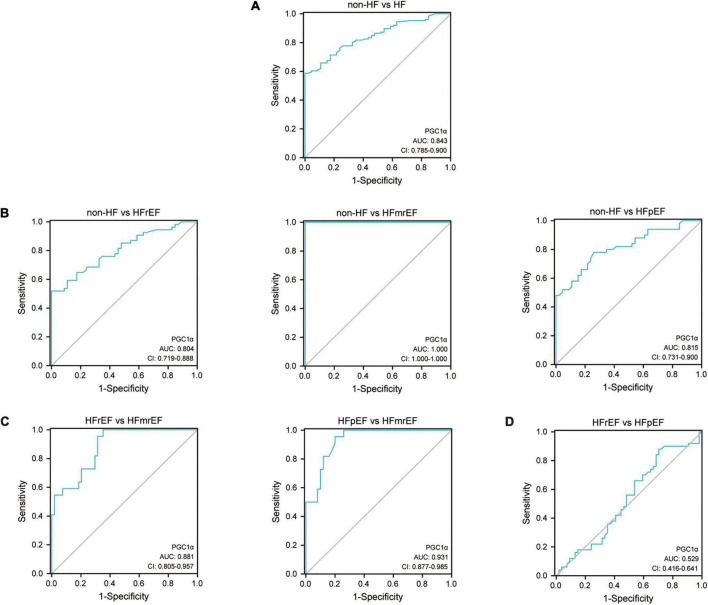
ROC curve of PGC1α in diagnosing HF phenotypes. **(A)** The ROC curve of PGC1α in diagnosing HF compared with non-HF. **(B)** The ROC curve of PGC1α in identifying various HF phenotypes compared with non-HF. **(C)** The ROC curve of PGC1α in distinguishing HFmrEF from the other two HF phenotypes. **(D)** The ROC curve regarding the comparison of PGC1α between HFrEF and HFpEF. ROC, receiver operating characteristic; AUC, area under the curve; CI, confidence interval; PGC1α, peroxisome proliferator-activated receptor-γ coactivator-1α; HF, heart failure; HFrEF, heart failure with reduced ejection fraction; HFmrEF, heart failure with mildly reduced ejection fraction; HFpEF, heart failure with preserved ejection fraction.

The ROC curve analysis showed that the AUC of PGC1α for the diagnosis of HF compared to non-HF was 0.843 (CI: 0.785–0.900), and the cut-off criterium was 293.918 nmol/L, suggesting that 293.918 could be used as the criterion for distinguishing HF from non-HF. Additionally, the AUC for diagnosing HFrEF, HFmrEF, and HFpEF compared to non-HF was 0.804, 1.000, and 0.815, respectively. The above-mentioned results revealed the ideal diagnostic value in identifying HF and its phenotypes from non-HF controls (Figure 3). Furthermore, the AUC regarding distinguishing HFmrEF from HFrEF and HFpEF was 0.881 and 0.931, respectively, and the cut-off criteria were 332.818 and 348.132, suggesting its powerful value in identifying HFmrEF from the other two HF phenotypes. The detailed statistical analysis is listed in Table 4.

**TABLE 4:** Statistical analysis of ROC curve in identifying HF and each HF phenotypes.

Group	AUC	95%CI	Sensitivity	Specificity	Youden index	Cut-off criteria
Non-HF vs. HF	0.843	0.785–0.900	0.587	1.000	0.587	293.918
Non-HF vs. HFrEF	0.804	0.719–0.888	0.519	1.000	0.519	294.535
Non-HF vs. HFmrEF	1.000	1.000–1.000	1.000	1.000	1.000	314.220
Non-HF vs. HFpEF	0.815	0.731–0.900	0.780	0.739	0.519	255.705
HFrEF vs. HFmrEF	0.881	0.805–0.957	1.000	0.648	0.648	332.818
HFpEF vs. HFmrEF	0.931	0.877–0.985	0.955	0.800	0.755	348.132
HFrEF vs. HFpEF	0.529	0.416–0.641	0.880	0.296	0.176	/

ROC, receiver operative characteristics curve; non-HF, non-heart failure participants; HF, heart failure; HFrEF, heart failure with reduced ejection fraction; HFmrEF, heart failure with mildly reduced ejection fraction; HFpEF, heart failure with preserved ejection fraction; AUC, area under the curve; CI, confidence interval.

## Discussion

Compared to the most widely accepted categories of HF (HFrEF and HFpEF), HFmrEF represents a borderline group that has received little attention, lacking effective diagnostic methods and treatment (15). However, HFmrEF accounts for approximately 24.2% of all HF cases in the European Society of Cardiology (ESC) HF Long-Term Registry, which includes all regions of European and Mediterranean countries (16). Therefore, focusing on HFmrEF and trying to discover biomarkers for identifying HFmrEF is of great significance. Herein, we discovered that PGC1α could serve as the potential marker to differentiate HFmrEF from the other two HF phenotypes.

PGC1α has been initially identified as a cofactor for nuclear hormone receptor peroxisome proliferator-activated receptor gamma (PPARγ) in adipocytes, which is required for the adaptive thermogenic response to a lower temperature (17). It regulates several metabolic processes in cardiovascular disease by interacting with various downstream effectors. Monitoring and regulating the expression of PGC1α is essential for improving cardiac function. The importance of PGC1α in the cardiovascular system has been extensively studied in basic research. For instance, Ding et al. (18) have discovered that mitochondrial fission in diabetic hearts could be prevented via regulating the Sirt1-PGC1α pathway. Cui et al. (19) have confirmed that pathological cardiac hypertrophy could be alleviated by reducing oxidation, inflammation, and apoptosis induced by AngII via Sirt1-mediated activation of AMPK/PGC1α signal molecules. Zhou et al. (20) have found that nobiletin could attenuate post-myocardial infarction pathological cardiac remodeling via upregulating PGC1α. Besides, several new HF drugs exert their function via activating PGC1α. Packer (21) has summarized that SGLT2-inhibitors could protect HF both from activating the Sirt1/PGC1α/FGF21 signaling transition and directly upregulating the expression of Sirt1, PGC1α, and FGF21.

In our study, we revealed that PGC1α had the highest expression level in the HFmrEF group, which could be considered the transitory stage from HFpEF to HFrEF. PGC1α participates in mitochondrial biosynthesis (22); hence, we speculated that patients with HFmrEF had high metabolism status, indicating that the body was working hard to maintain or recover cardiac function. Also, when a patient had HFrEF, the PGC1α expression was once again downregulated compared with HFmrEF, indicating the failure to control HF. Therefore, since PGC1α expression in HFpEF patients is upregulated, we should pay more attention to modifying the use of drugs and medical devices.

The value of PGC1α in diagnosing HF and identifying HF phenotypes is important, as shown on the ROC curve. The AUCs of the four groups were all greater than 0.8. Furthermore, AUC and Youden index were equal to 1in HFmrEF, showing the highest sensitivity and specificity in diagnosing HFmrEF. Meanwhile, we established a detailed threshold of PGC1α in diagnosing different HF phenotypes, which could be helpful for clinical practice. Additionally, we conducted the correlation analysis using Pearson test to investigate the relationship between PGC1α and blood glucose/NT-proBNP in HFmrEF patients. The results showed that there was no statistically significant correlation between PGC1α and blood glucose/NT-proBNP in HFmrEF group (Supplementary Figure 1). Therefore, we speculated that PGC1α could be used as a new biomarker with a different mechanism comparing with blood glucose/NT-proBNP in HFmrEF pathogenesis.

The newest AHA/ACC/HFSA Guideline for the Management of Heart Failure (23) published in April 2022 prompted a new category of HF: HF with improved ejection fraction (HFimpEF). Patients with HFrEF who improve ejection fraction to over 40% should be considered HFimpEF patients. This is a new concept, and whether PGC1α is valuable in this category requires further investigation. Besides, despite the fact that our study demonstrated the importance of PGC1α in identifying various HF phenotypes, more experimental studies should be conducted to further confirm the PGC1α expression in various phenotypes. In addition to several classical models for HFrEF, there are a few ideal models for HFmrEF and HFpEF. More basic models should be developed.

## Conclusion

We discovered that PGC1α expression was significantly upregulated in HF patients compared with non-HF participants. Besides, HFmrEF patients had a higher PGC1α expression level than HFrEF and HFpEF patients, which could be employed as a potential biomarker for differentiating HF patients from those without HF, as well as for distinguishing HFmrEF from HFrEF and HFpEF.

## Data availability statement

The raw data supporting the conclusions of this article will be made available by the authors, without undue reservation.

## Ethics statement

The study protocol was approved by the Ethics Committee of the Affiliated Hospital of Xuzhou Medical University (Xuzhou, China) (Approval number: XYFY2021-KL116-01; May 25, 2021). The patients/participants provided their written informed consent to participate in this study.

## Author contributions

SZ and YH contributed to hypothesis development and manuscript preparation. SZ and YZ contributed to the study design, drafted and revised the manuscript. SZ was responsible for collecting clinical samples. YM and ZL undertook data analyses. All authors contributed to the article and approved the submitted version.

## References

[1] Roger VL. Epidemiology of heart failure: A contemporary perspective. *Circ Res.* (2021) 128:1421–34. 10.1161/circresaha.121.318172 33983838

[2] Bauersachs J. Heart failure drug treatment: The fantastic four. *Eur Heart J.* (2021) 42:681–3. 10.1093/eurheartj/ehaa1012 33447845 PMC7878007

[3] McDonagh TA Metra M Adamo M Gardner RS Baumbach A Böhm M 2021 ESC Guidelines for the diagnosis and treatment of acute and chronic heart failure. *Eur Heart J.* (2021) 42:3599–726. 10.1093/eurheartj/ehab368 34447992

[4] Pfeffer MA Shah AM Borlaug BA. Heart failure with preserved ejection fraction in perspective. *Circ Res.* (2019) 124:1598–617. 10.1161/circresaha.119.313572 31120821 PMC6534165

[5] van der Meer P Gaggin HK Dec GW. ACC/AHA versus ESC guidelines on heart failure: JACC guideline comparison. *J Am Coll Cardiol.* (2019) 73:2756–68. 10.1016/j.jacc.2019.03.478 31146820

[6] Lopaschuk GD Karwi QG Tian R Wende AR Abel ED. Cardiac energy metabolism in heart failure. *Circ Res.* (2021) 128:1487–513. 10.1161/circresaha.121.318241 33983836 PMC8136750

[7] Lopaschuk GD Jaswal JS. Energy metabolic phenotype of the cardiomyocyte during development, differentiation, and postnatal maturation. *J Cardiovasc Pharmacol.* (2010) 56:130–40. 10.1097/FJC.0b013e3181e74a14 20505524

[8] Bertero E Maack C. Metabolic remodelling in heart failure. *Nat Rev Cardiol.* (2018) 15:457–70. 10.1038/s41569-018-0044-6 29915254

[9] Tian L Cao W Yue R Yuan Y Guo X Qin D Pretreatment with Tilianin improves mitochondrial energy metabolism and oxidative stress in rats with myocardial ischemia/reperfusion injury via AMPK/SIRT1/PGC-1 alpha signaling pathway. *J Pharmacol Sci.* (2019) 139:352–60. 10.1016/j.jphs.2019.02.008 30910451

[10] Russomanno G Corbi G Manzo V Ferrara N Rengo G Puca AA The anti-ageing molecule sirt1 mediates beneficial effects of cardiac rehabilitation. *Immun Ageing.* (2017) 14:7. 10.1186/s12979-017-0088-1 28331525 PMC5353800

[11] Corbi G Conti V Troisi J Colucci A Manzo V Di Pietro P Cardiac rehabilitation increases SIRT1 activity and β-hydroxybutyrate levels and decreases oxidative stress in patients with HF with preserved ejection fraction. *Oxid Med Cell Longev.* (2019) 2019:7049237. 10.1155/2019/7049237 31885811 PMC6900956

[12] Zhang L Chen J Yan L He Q Xie H Chen M. Resveratrol ameliorates cardiac remodeling in a murine model of heart failure with preserved ejection fraction. *Front Pharmacol.* (2021) 12:646240. 10.3389/fphar.2021.646240 34177571 PMC8225267

[13] He L Ma S Zuo Q Zhang G Wang Z Zhang T An effective sodium-dependent glucose transporter 2 inhibition, canagliflozin, prevents development of hypertensive heart failure in Dahl salt-sensitive rats. *Front Pharmacol.* (2022) 13:856386. 10.3389/fphar.2022.856386 35370704 PMC8964360

[14] Conti V Corbi G Polito MV Ciccarelli M Manzo V Torsiello M Sirt1 activity in PBMCs as a biomarker of different heart failure phenotypes. *Biomolecules.* (2020) 10:1590. 10.3390/biom10111590 33238655 PMC7700185

[15] Hsu JJ Ziaeian B Fonarow GC. Heart failure with mid-range (borderline) ejection fraction: Clinical implications and future directions. *JACC Heart Fail.* (2017) 5:763–71. 10.1016/j.jchf.2017.06.013 29032140 PMC6668914

[16] Chioncel O Lainscak M Seferovic PM Anker SD Crespo-Leiro MG Harjola VP Epidemiology and one-year outcomes in patients with chronic heart failure and preserved, mid-range and reduced ejection fraction: An analysis of the ESC Heart Failure Long-Term Registry. *Eur J Heart Fail.* (2017) 19:1574–85. 10.1002/ejhf.813 28386917

[17] Puigserver P Wu Z Park CW Graves R Wright M Spiegelman BM. A cold-inducible coactivator of nuclear receptors linked to adaptive thermogenesis. *Cell.* (1998) 92:829–39. 10.1016/s0092-8674(00)81410-5 9529258

[18] Ding M Feng N Tang D Feng J Li Z Jia M Melatonin prevents Drp1-mediated mitochondrial fission in diabetic hearts through SIRT1-PGC1α pathway. *J Pineal Res.* (2018) 65:e12491. 10.1111/jpi.12491 29575122 PMC6099285

[19] Cui YK Hong YX Wu WY Han WM Wu Y Wu C Acacetin ameliorates cardiac hypertrophy by activating Sirt1/AMPK/PGC-1α pathway. *Eur J Pharmacol.* (2022) 920:174858. 10.1016/j.ejphar.2022.174858 35219729

[20] Zhou Y Yin T Shi M Chen M Wu X Wang K Nobiletin attenuates pathological cardiac remodeling after myocardial infarction via activating PPARγ and PGC1α. *PPAR Res.* (2021) 2021:9947656. 10.1155/2021/9947656 34422028 PMC8373512

[21] Packer M. Cardioprotective effects of sirtuin-1 and its downstream effectors: Potential role in mediating the heart failure benefits of SGLT2 (sodium-glucose cotransporter 2) inhibitors. *Circ Heart Fail.* (2020) 13:e007197. 10.1161/circheartfailure.120.007197 32894987

[22] Huang Q Su H Qi B Wang Y Yan K Wang X A SIRT1 activator, Ginsenoside Rc, promotes energy metabolism in cardiomyocytes and neurons. *J Am Chem Soc.* (2021) 143:1416–27. 10.1021/jacs.0c10836 33439015

[23] Heidenreich PA Bozkurt B Aguilar D Allen LA Byun JJ Colvin MM 2022 AHA/ACC/HFSA Guideline for the management of heart failure: A report of the American College of Cardiology/American Heart Association Joint Committee on Clinical Practice Guidelines. *Circulation.* (2022) 145:e895–1032. 10.1161/cir.0000000000001063 35363499

