# Supplementary material for: The diagnostic value of peroxisome proliferator-activated receptor-γ coactivator-1α in identifying different chronic heart failure phenotypes
Source: Front Cardiovasc Med. 2022 Sep 6;9:973705. doi: 10.3389/fcvm.2022.973705 (PMC9485562; doi:10.3389/fcvm.2022.973705)
Supplement: Supplementary file 1 [file Data_Sheet_1.pdf]

Supplementary Data 1. The Q-Q plots of continuous variables regarding the main characteristics of the study population.

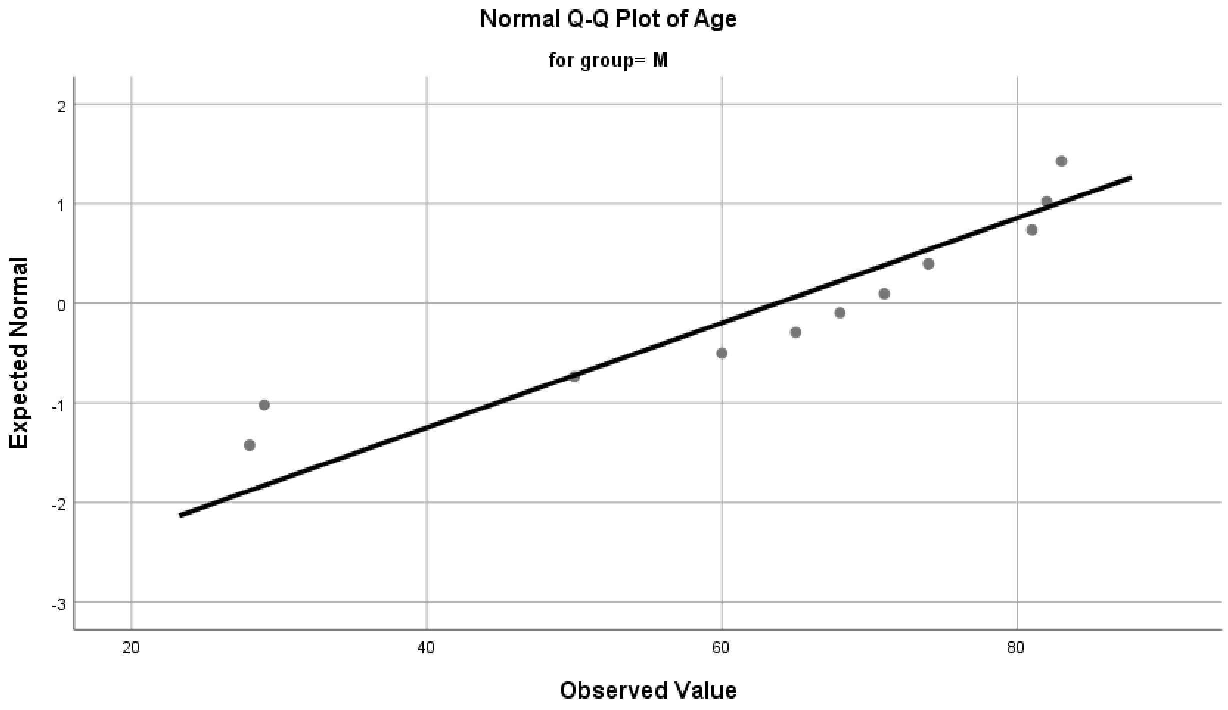

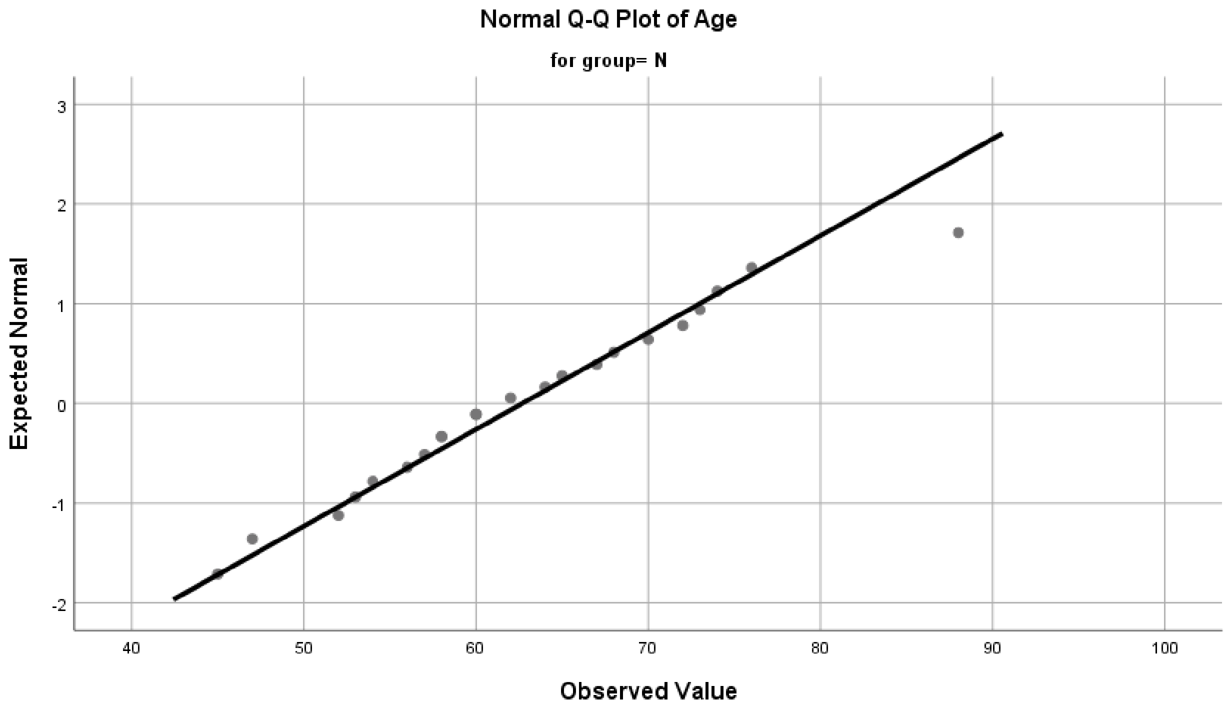

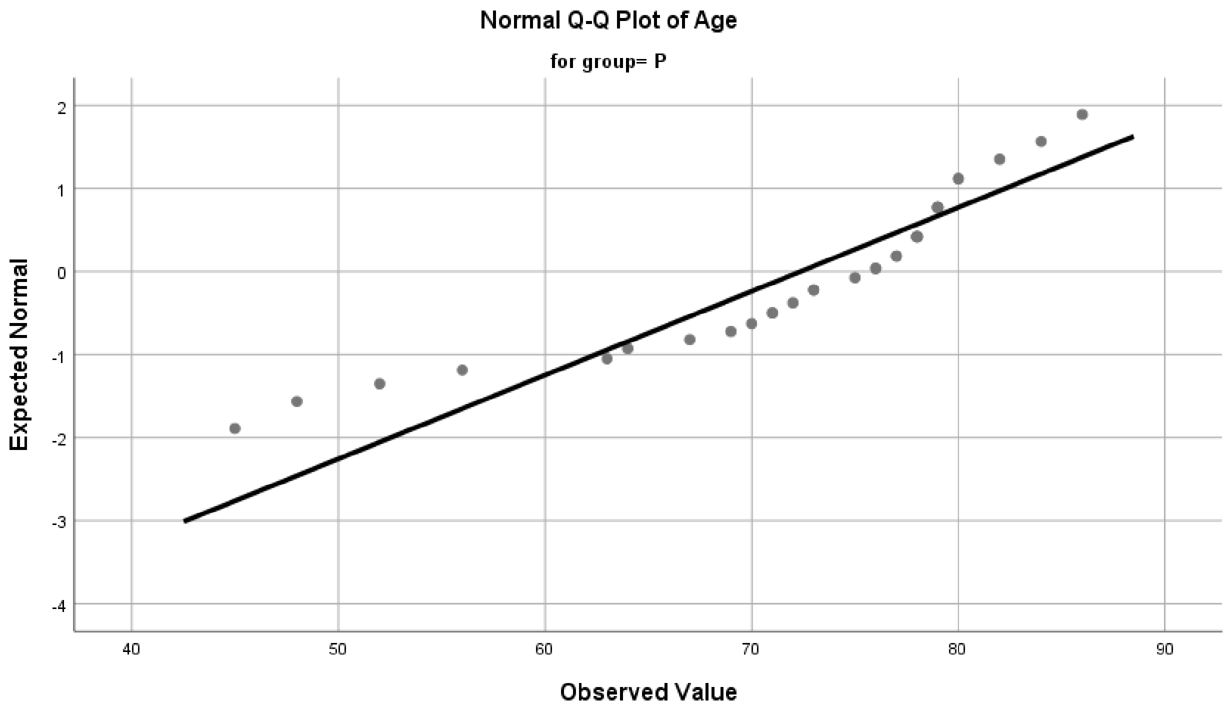
Normal Q-Q Plot of Age
for group= P
Expected Normal
Observed Value
2
1
0
-1
-2
-3
-4
40
50
60
70
80
90

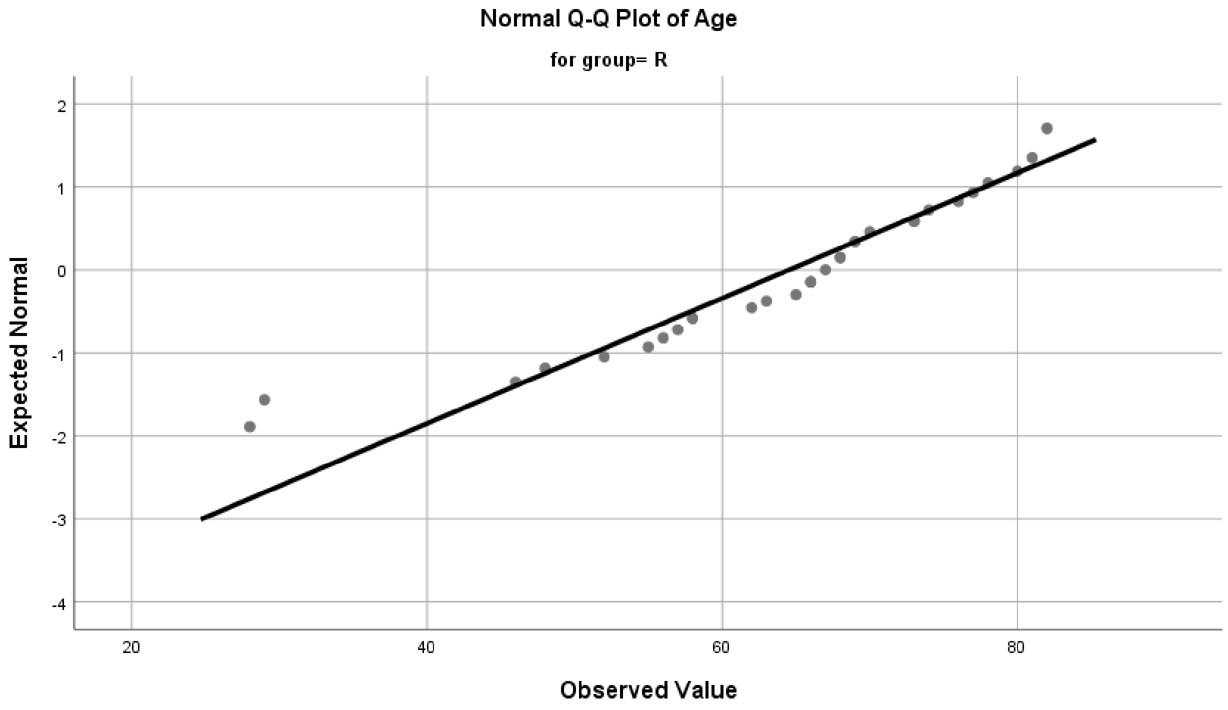
Normal Q-Q Plot of Age
for group= R
Expected Normal
Observed Value
2
1
0
-1
-2
-3
-4
20
40
60
80

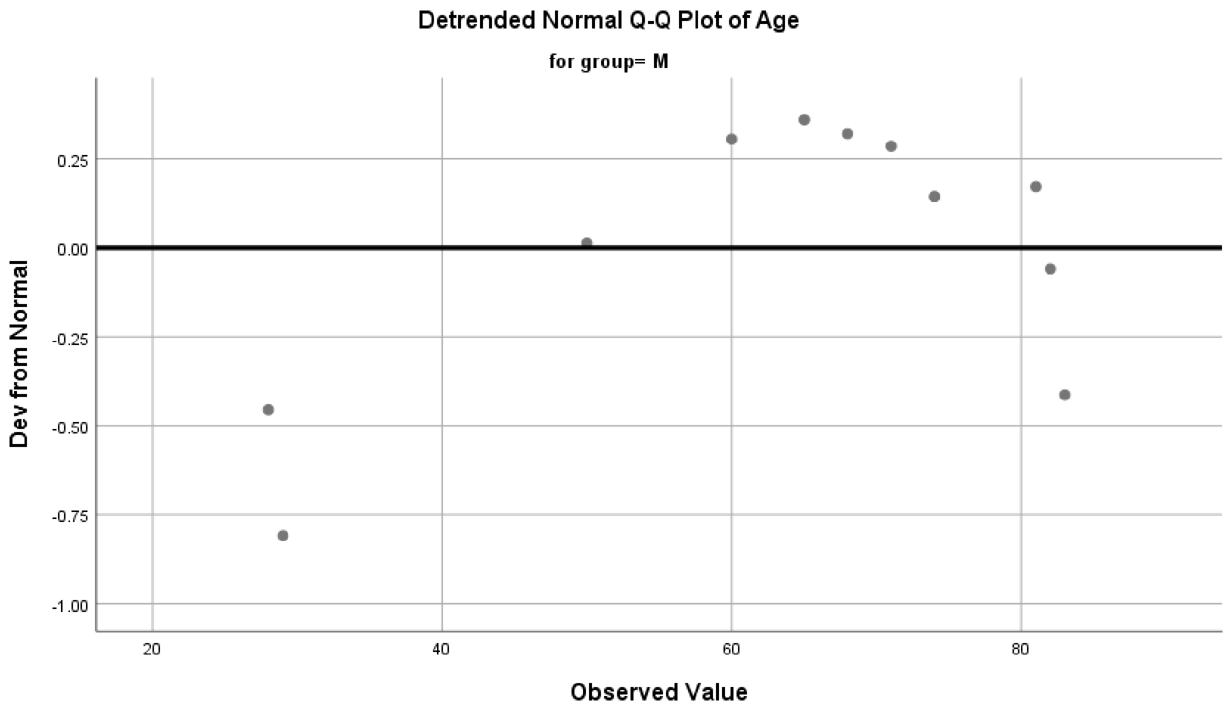
Detrended Normal Q-Q Plot of Age
for group= M
Dev from Normal
0.25
0.00
-0.25
-0.50
-0.75
-1.00
20
40
60
80
Observed Value

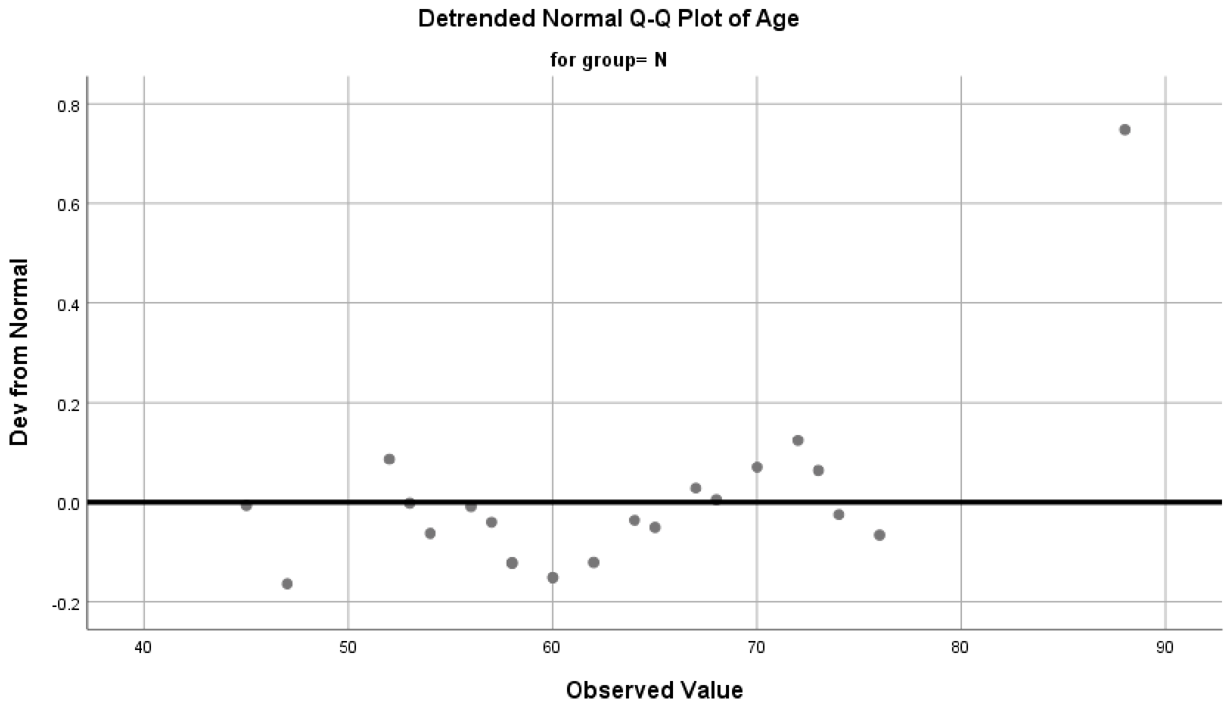
Detrended Normal Q-Q Plot of Age
for group= N
Dev from Normal
0.8
0.6
0.4
0.2
0.0
-0.2
40
50
60
70
80
90
Observed Value

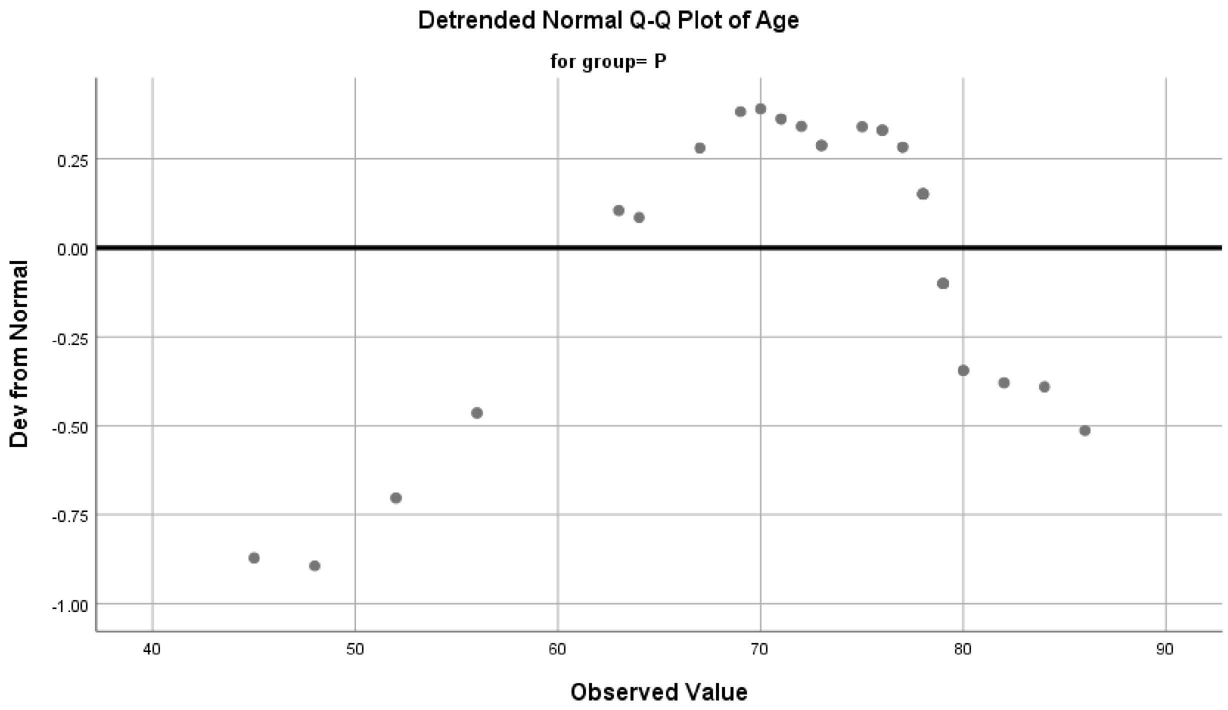

Detrended Normal Q-Q Plot of Age
for group= P
Dev from Normal
0.25
0.00
-0.25
-0.50
-0.75
-1.00
40
50
60
70
80
90
Observed Value

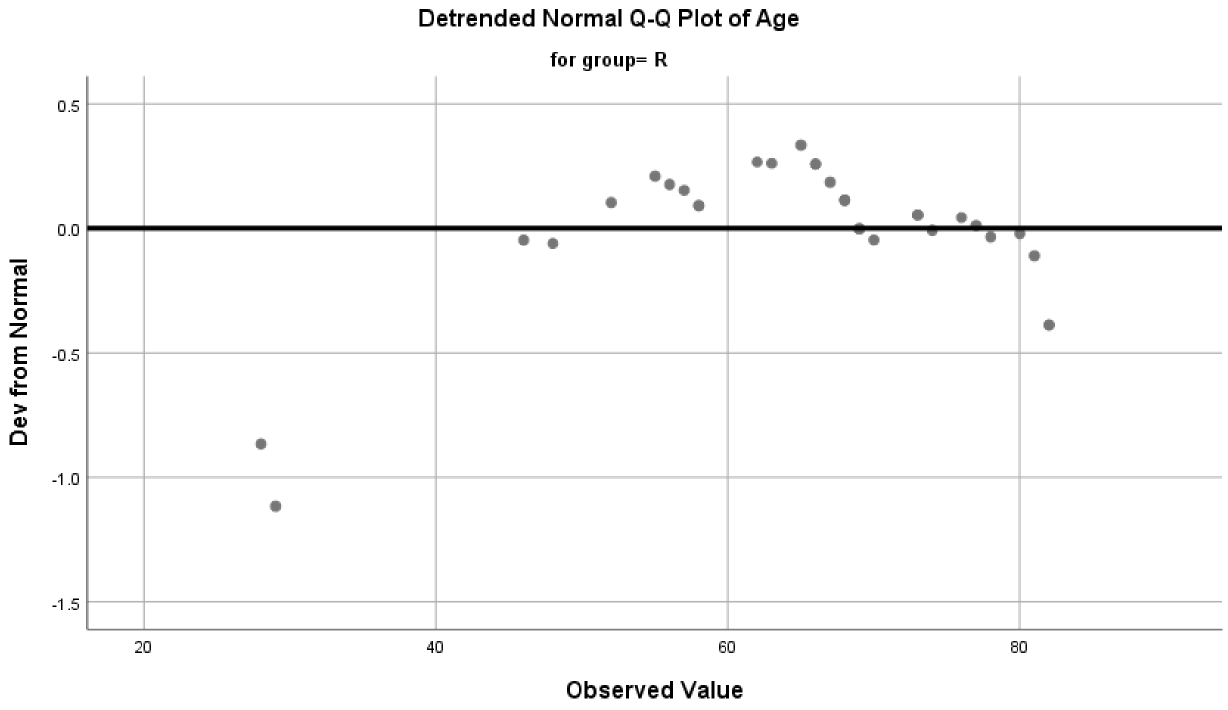

Detrended Normal Q-Q Plot of Age
for group= R
Dev from Normal
0.5
0.0
-0.5
-1.0
-1.5
20
40
60
80
Observed Value

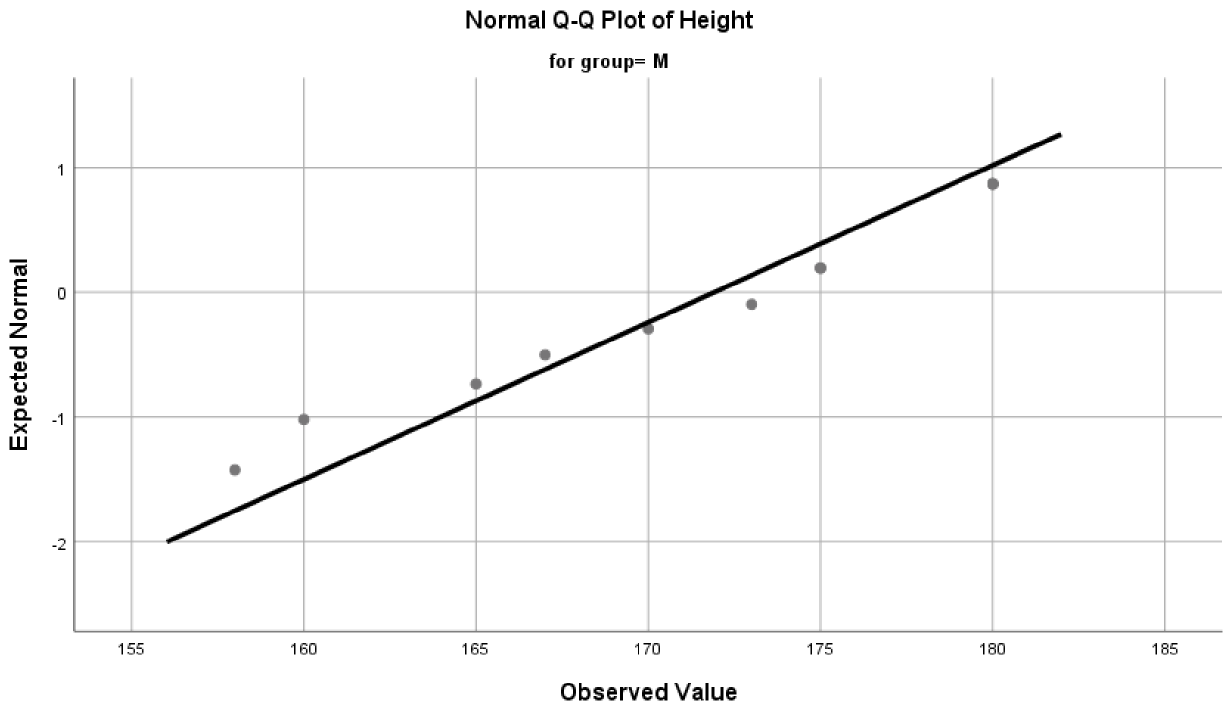

Normal Q-Q Plot of Height
for group= M
Expected Normal
Observed Value
1
0
-1
-2
155
160
165
170
175
180
185

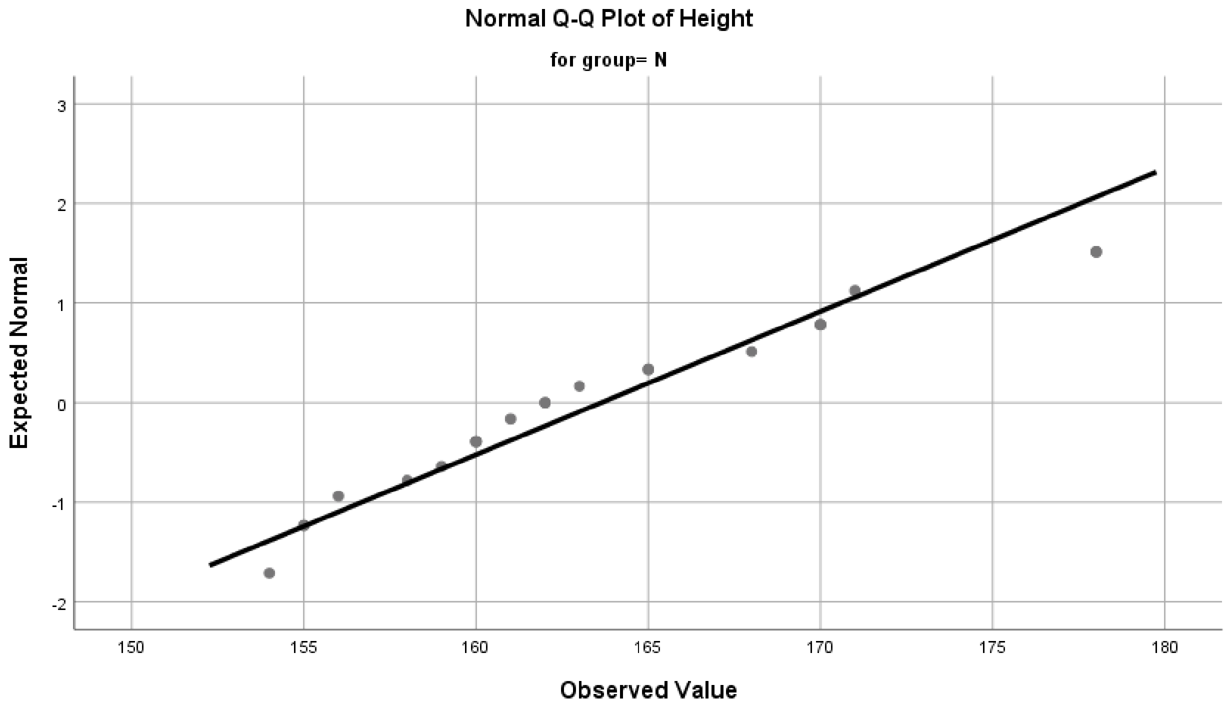

Normal Q-Q Plot of Height
for group= N
Expected Normal
Observed Value
3
2
1
0
-1
-2
150
155
160
165
170
175
180

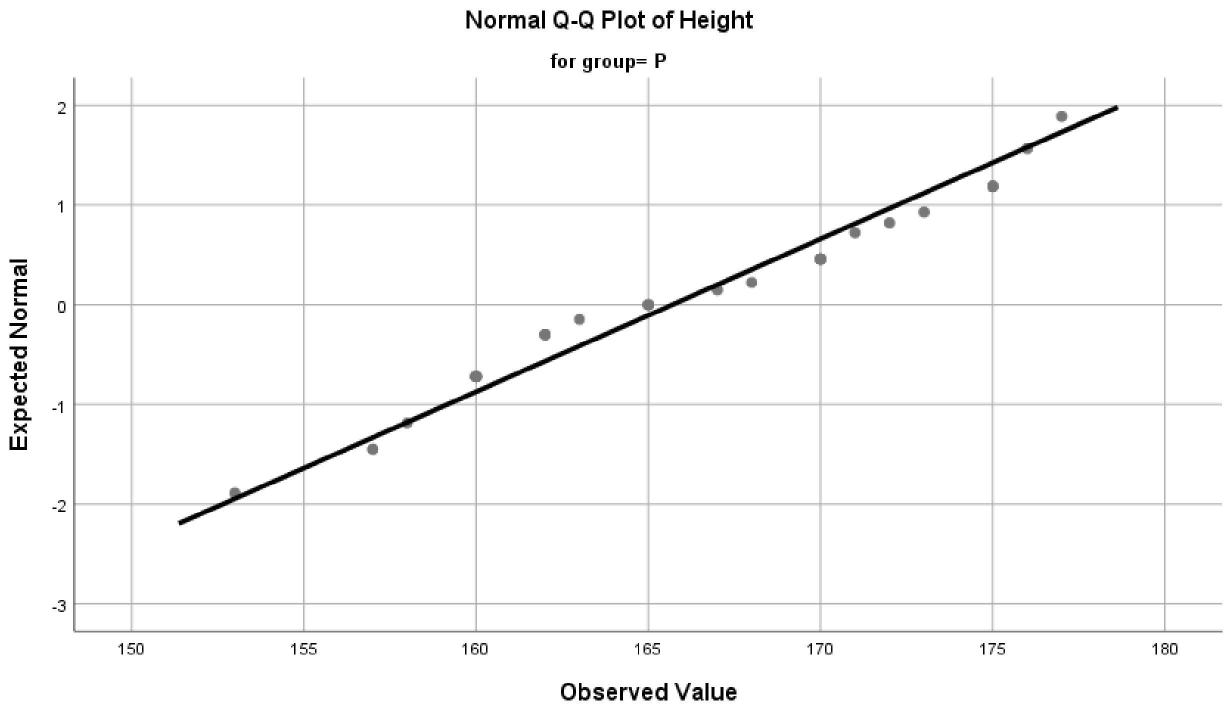
Normal Q-Q Plot of Height
for group= P
Expected Normal
Observed Value
2
1
0
-1
-2
-3
150
155
160
165
170
175
180

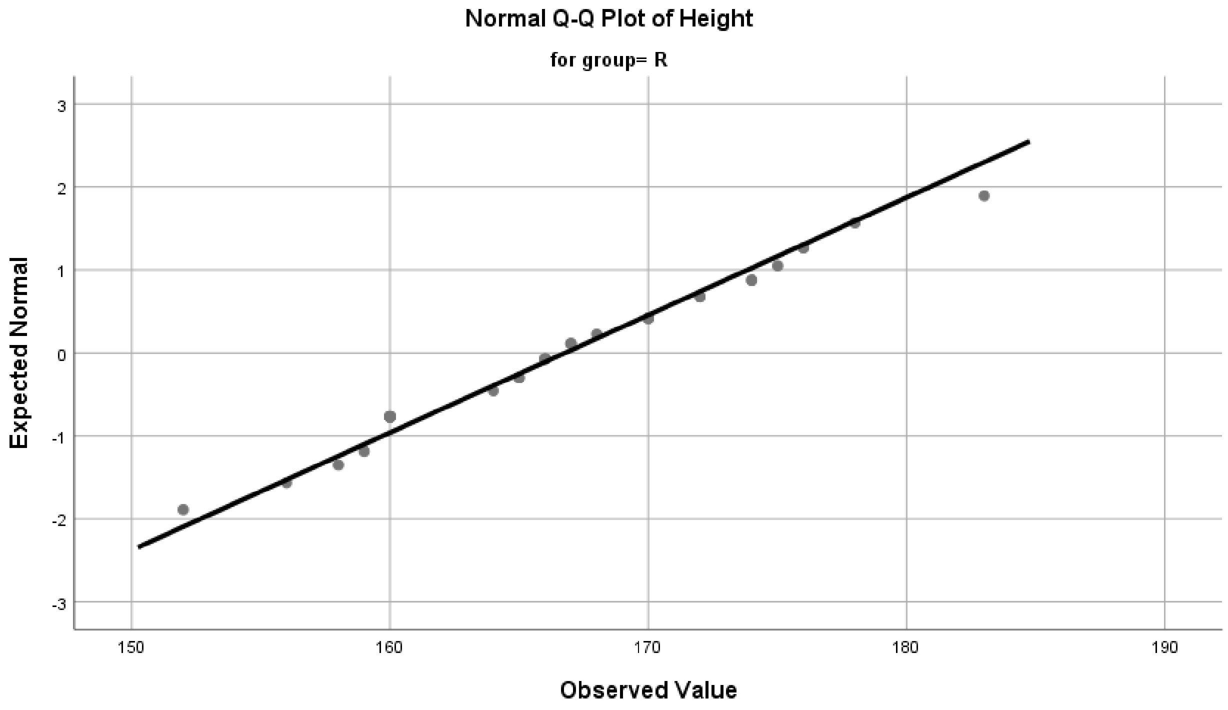
Normal Q-Q Plot of Height
for group= R
Expected Normal
Observed Value
3
2
1
0
-1
-2
-3
150
160
170
180
190

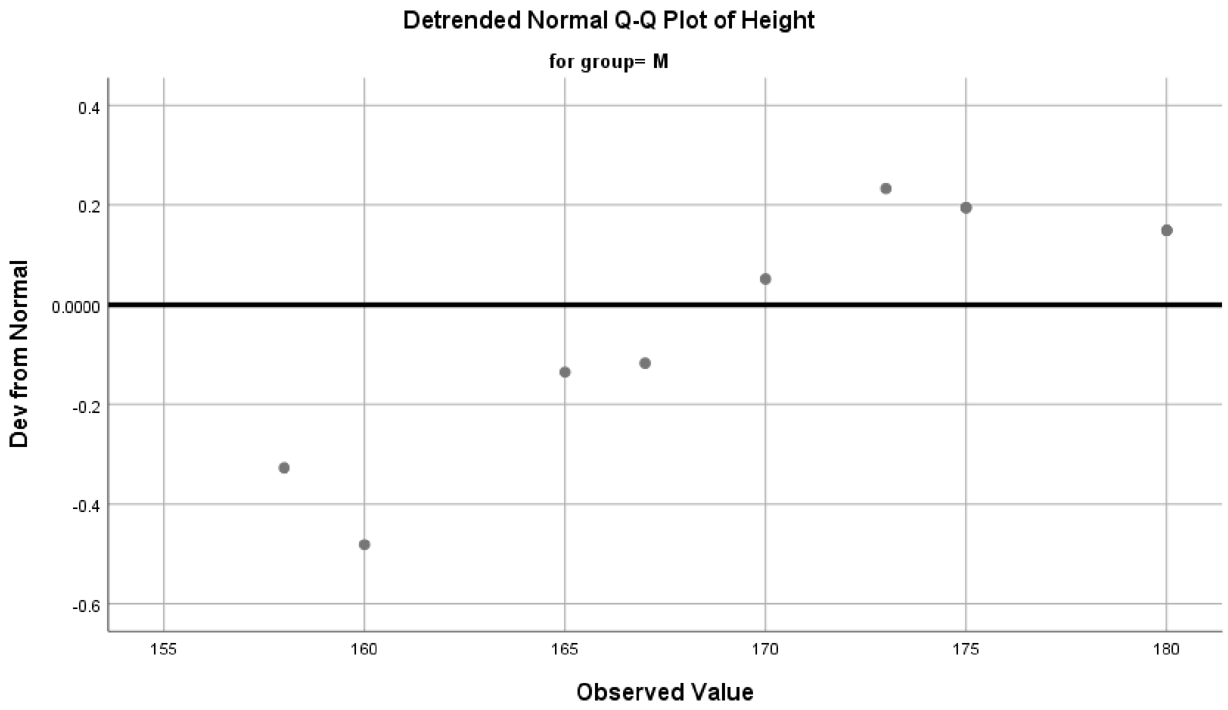

Detrended Normal Q-Q Plot of Height
for group= M
Dev from Normal
0.4
0.2
0.0000
-0.2
-0.4
-0.6
155
160
165
170
175
180
Observed Value

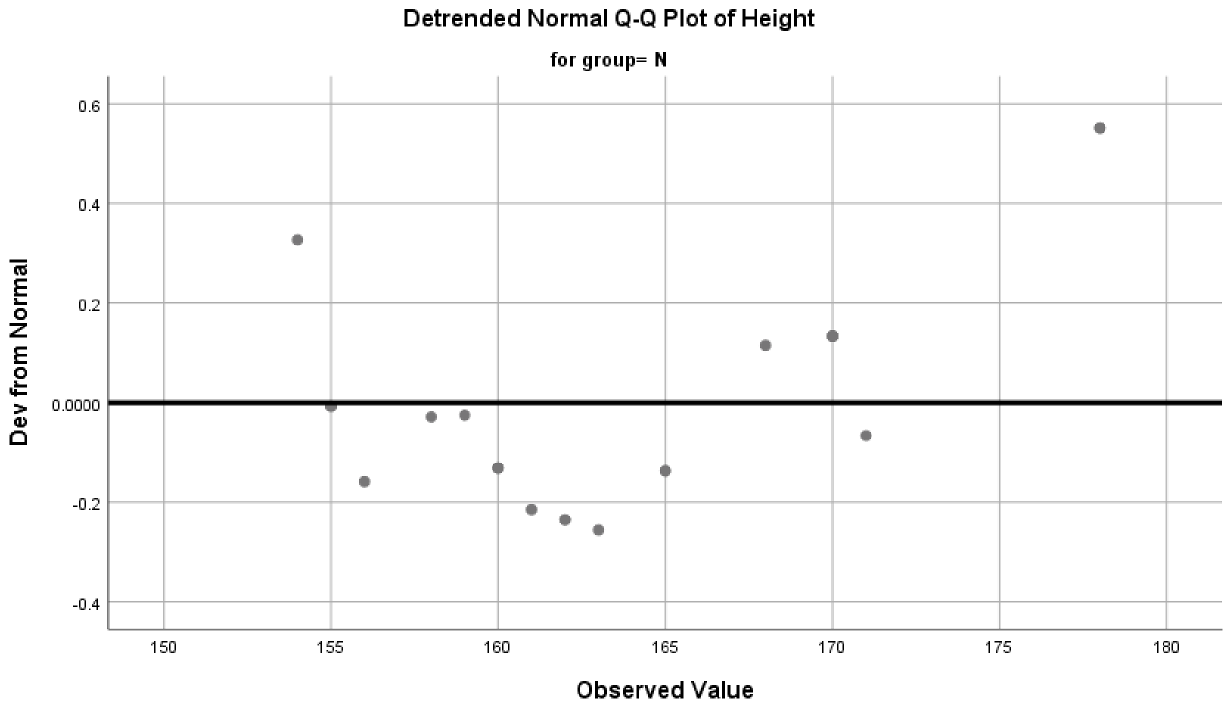

Detrended Normal Q-Q Plot of Height
for group= N
Dev from Normal
0.6
0.4
0.2
0.0000
-0.2
-0.4
150
155
160
165
170
175
180
Observed Value

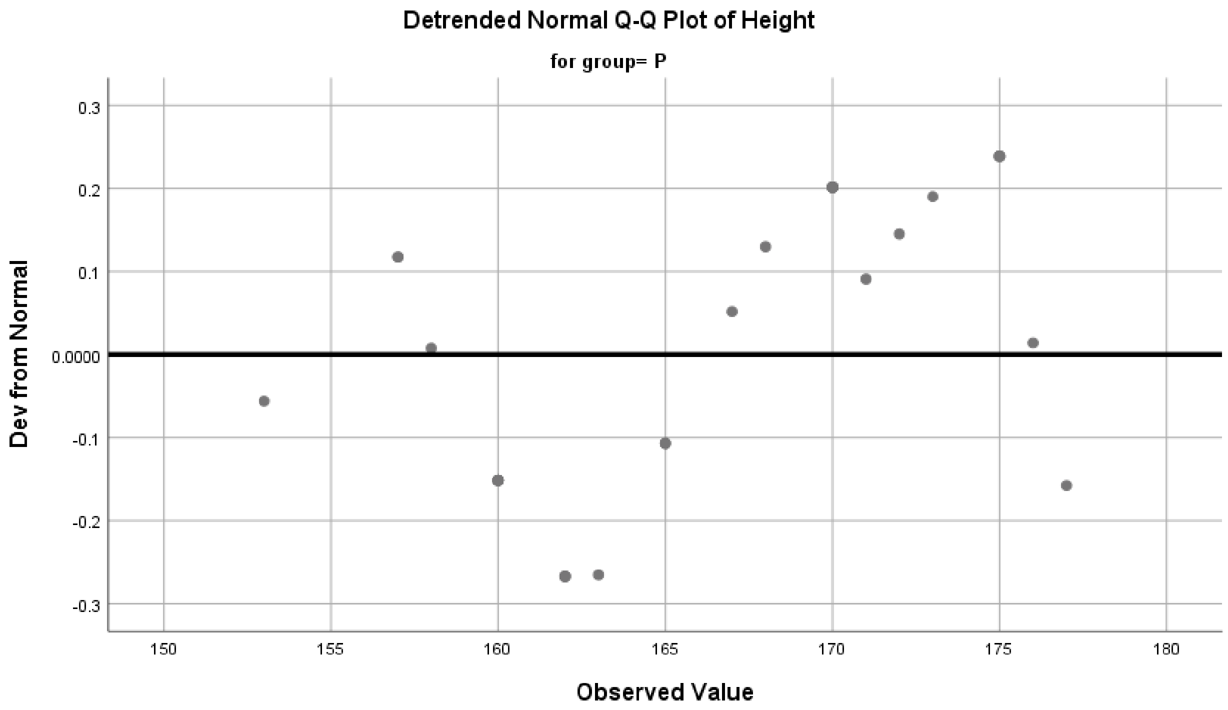

Detrended Normal Q-Q Plot of Height
for group= P
Dev from Normal
0.3
0.2
0.1
0.0000
-0.1
-0.2
-0.3
150
155
160
165
170
175
180
Observed Value

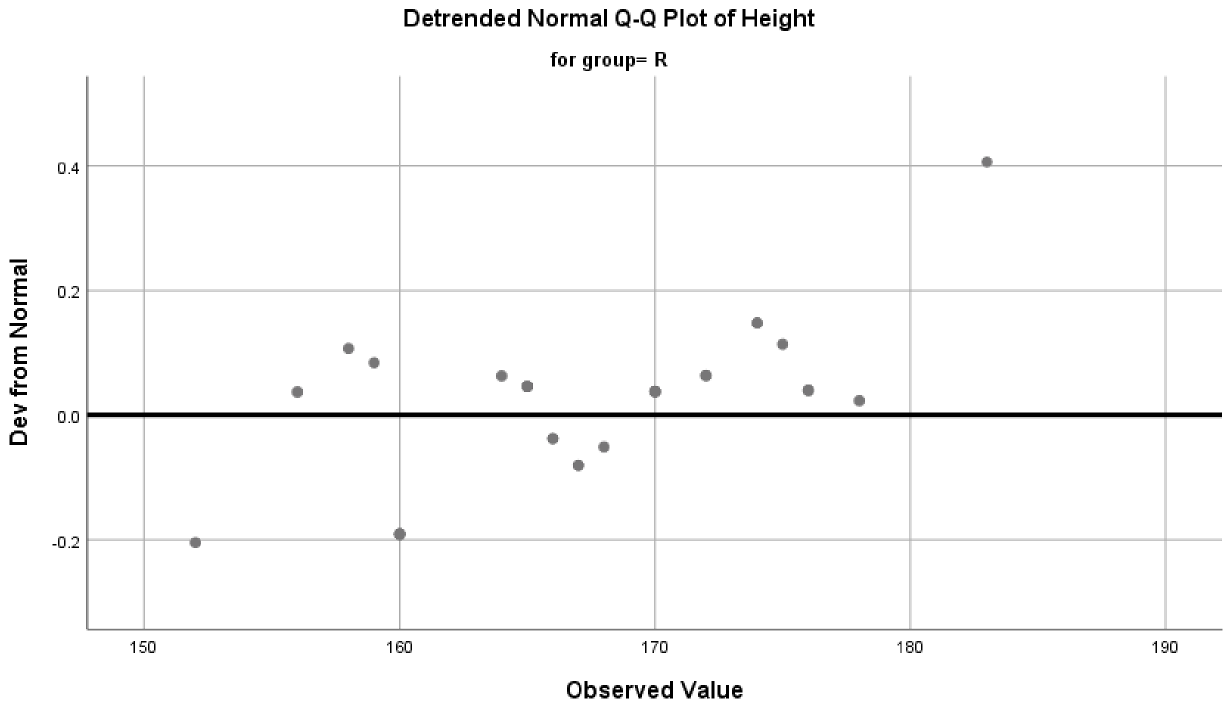

Detrended Normal Q-Q Plot of Height
for group= R
Dev from Normal
0.4
0.2
0.0
-0.2
150
160
170
180
190
Observed Value

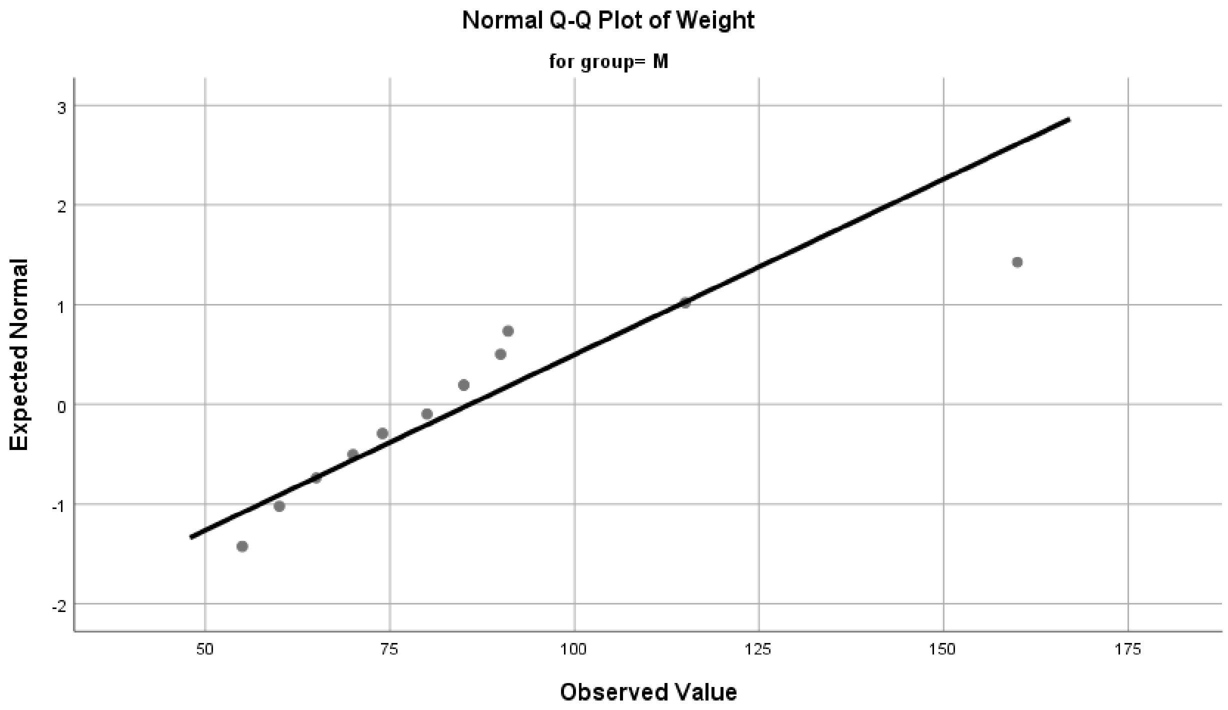
Normal Q-Q Plot of Weight
for group= M
Expected Normal
3
2
1
0
-1
-2
50
75
100
125
150
175
Observed Value

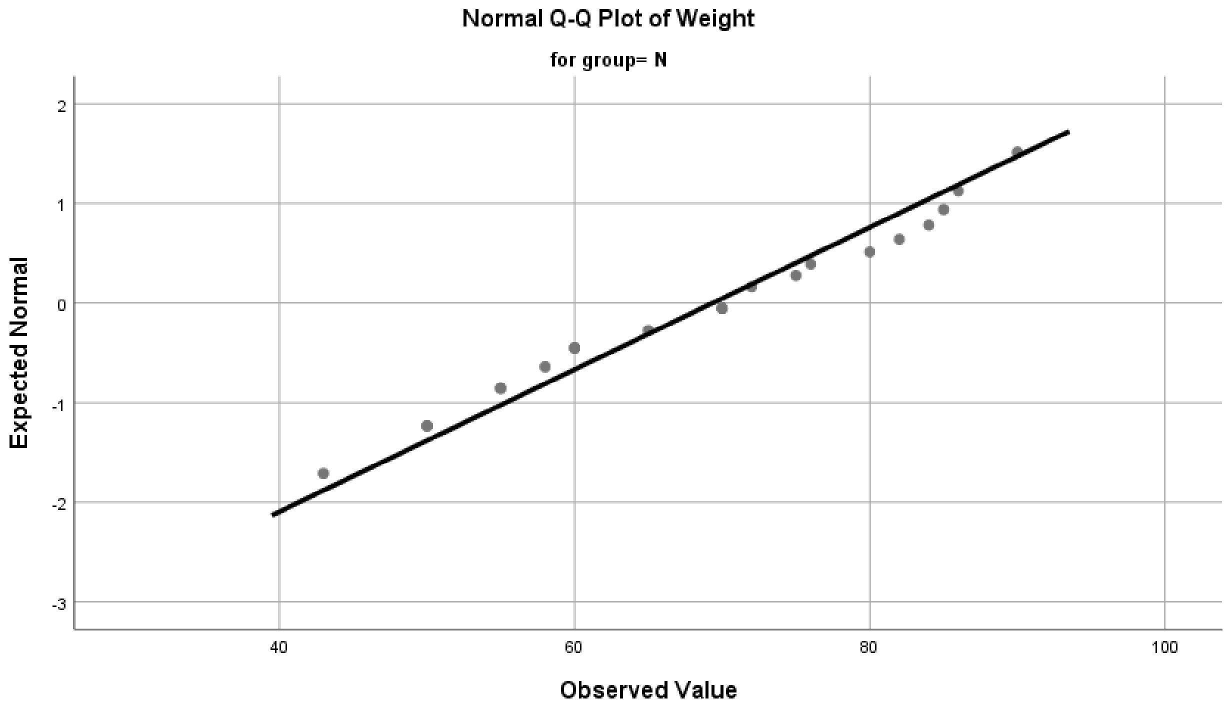
Normal Q-Q Plot of Weight
for group= N
Expected Normal
2
1
0
-1
-2
-3
40
60
80
100
Observed Value

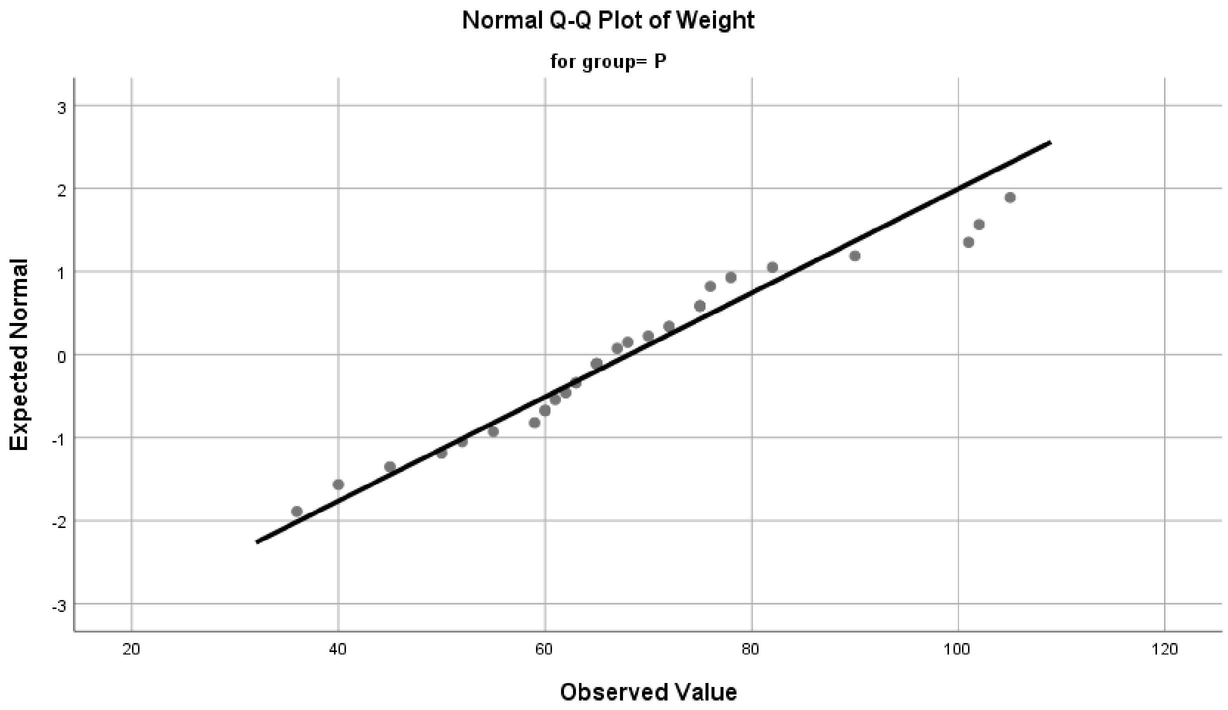
Normal Q-Q Plot of Weight
for group= P
Expected Normal
3
2
1
0
-1
-2
-3
20
40
60
80
100
120
Observed Value

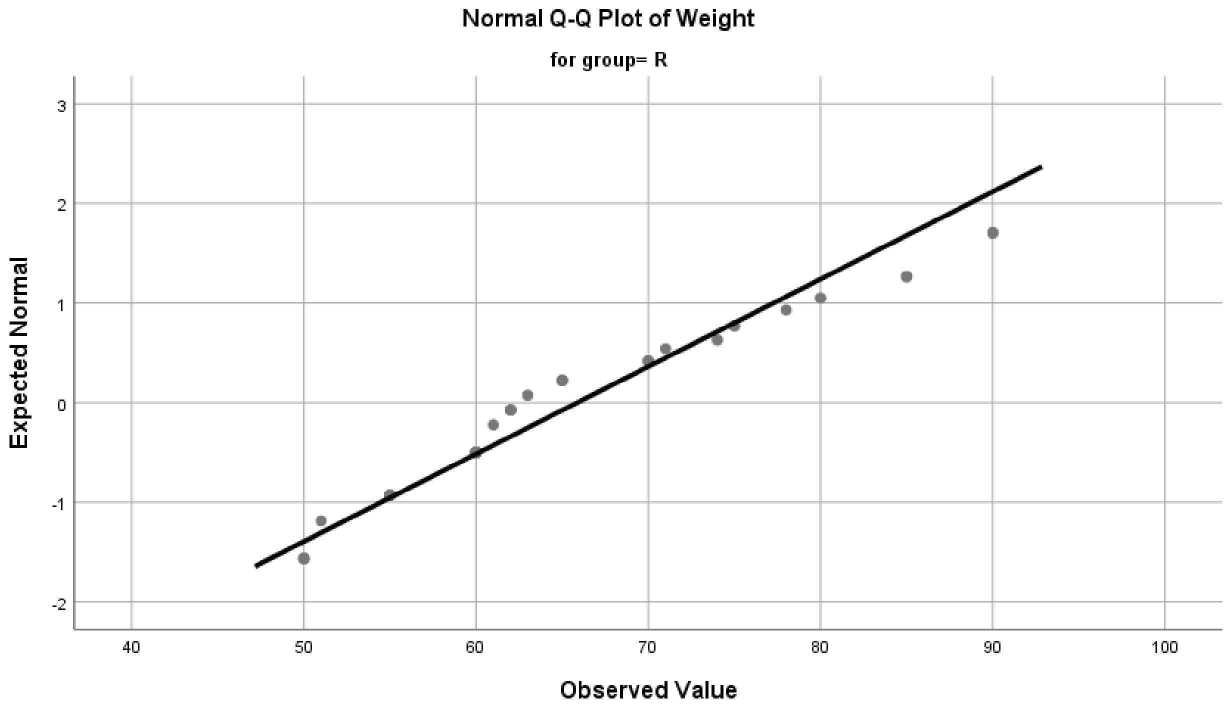
Normal Q-Q Plot of Weight
for group= R
Expected Normal
3
2
1
0
-1
-2
40
50
60
70
80
90
100
Observed Value

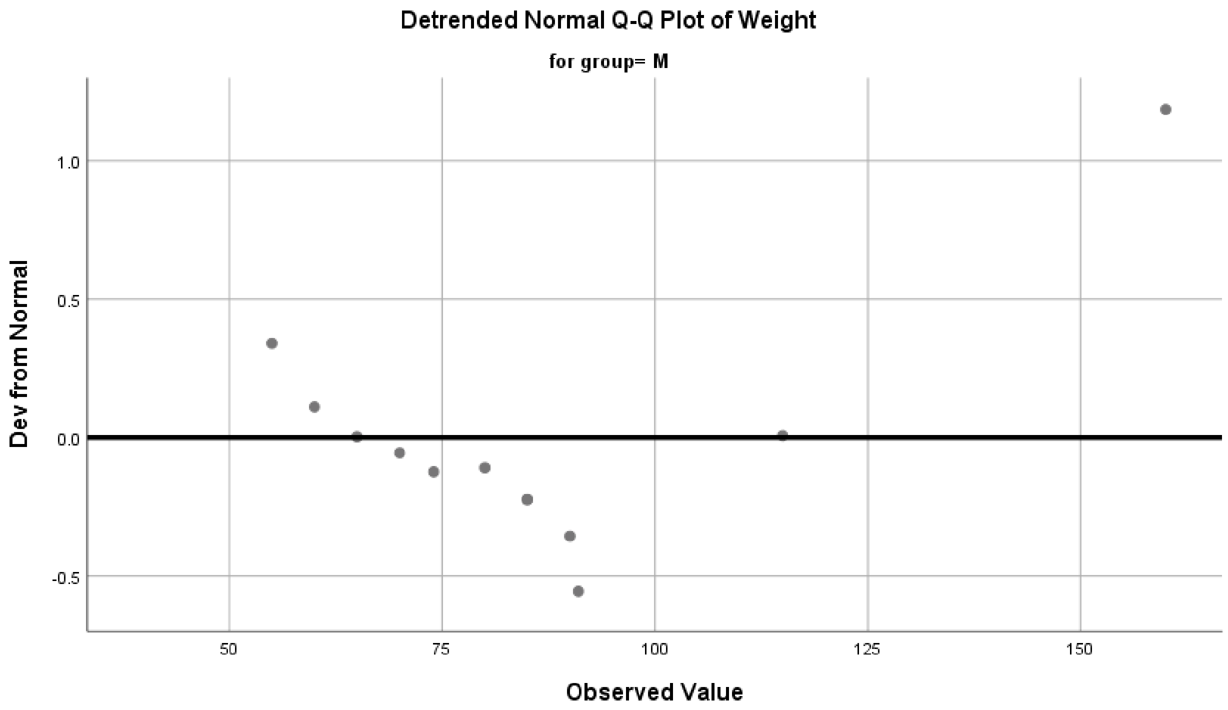

Detrended Normal Q-Q Plot of Weight
for group= M
Dev from Normal
1.0
0.5
0.0
-0.5
50
75
100
125
150
Observed Value

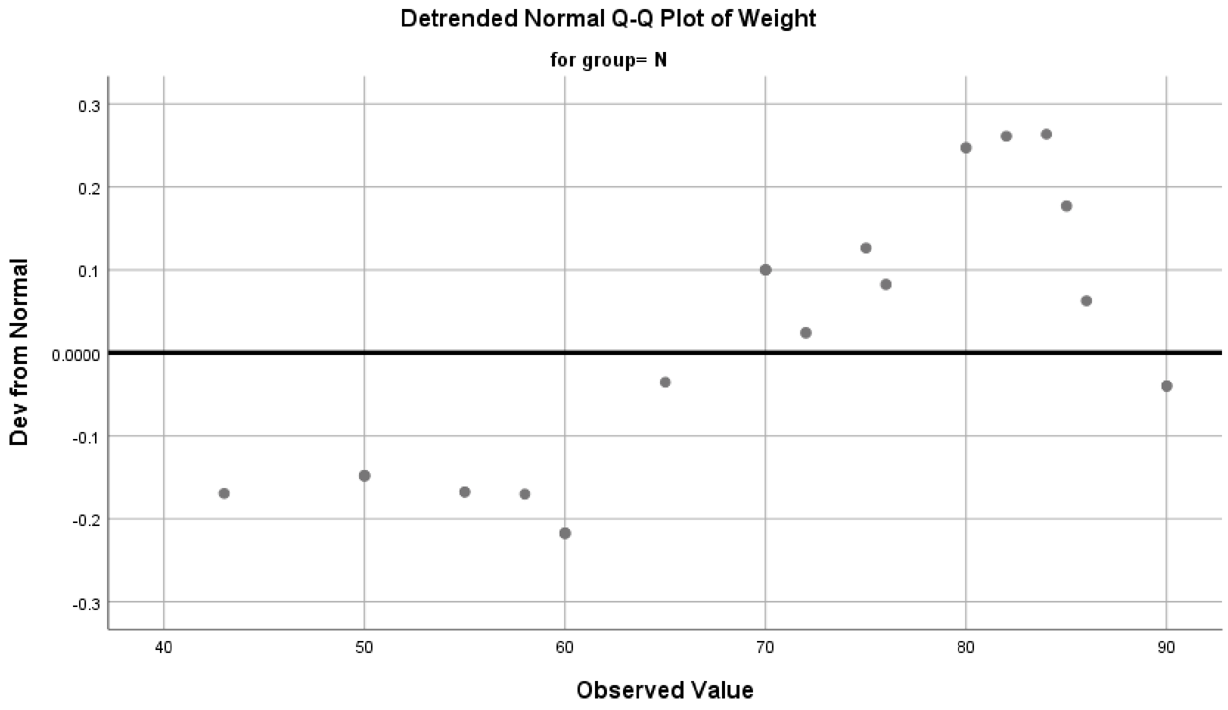

Detrended Normal Q-Q Plot of Weight
for group= N
Dev from Normal
0.3
0.2
0.1
0.0000
-0.1
-0.2
-0.3
40
50
60
70
80
90
Observed Value

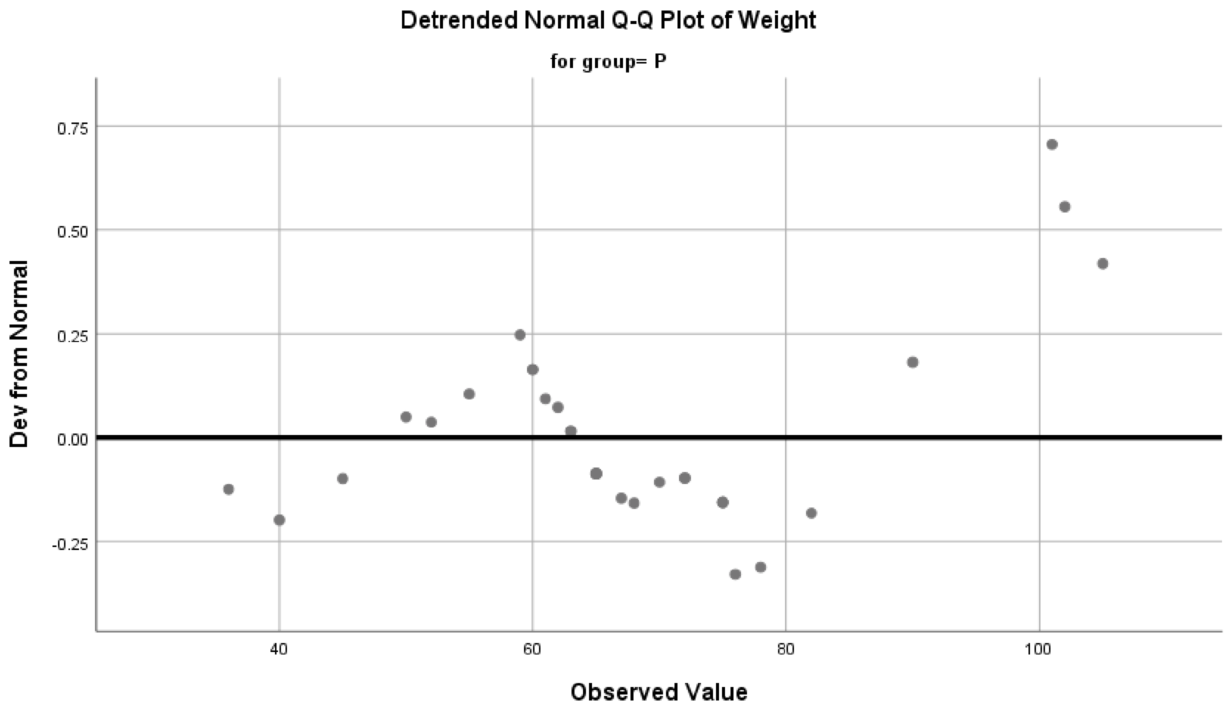
Detrended Normal Q-Q Plot of Weight
for group= P
Dev from Normal
0.75
0.50
0.25
0.00
-0.25
40
60
80
100
Observed Value

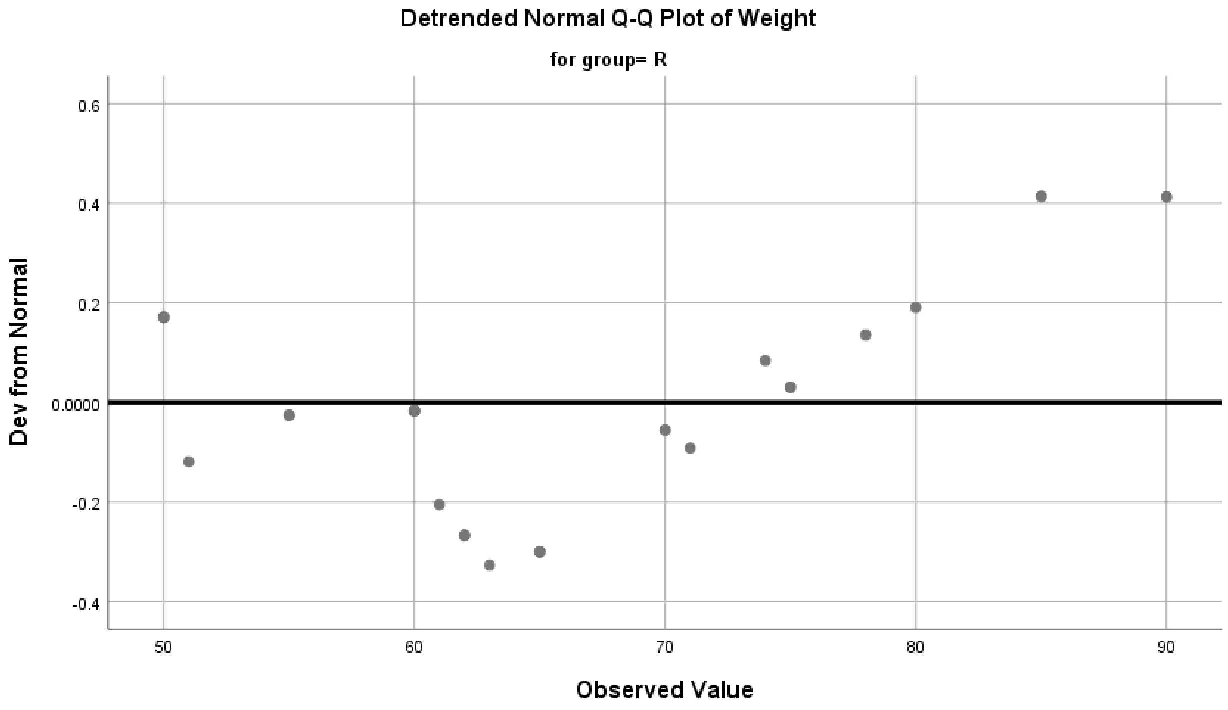
Detrended Normal Q-Q Plot of Weight
for group= R
Dev from Normal
0.6
0.4
0.2
0.0000
-0.2
-0.4
50
60
70
80
90
Observed Value

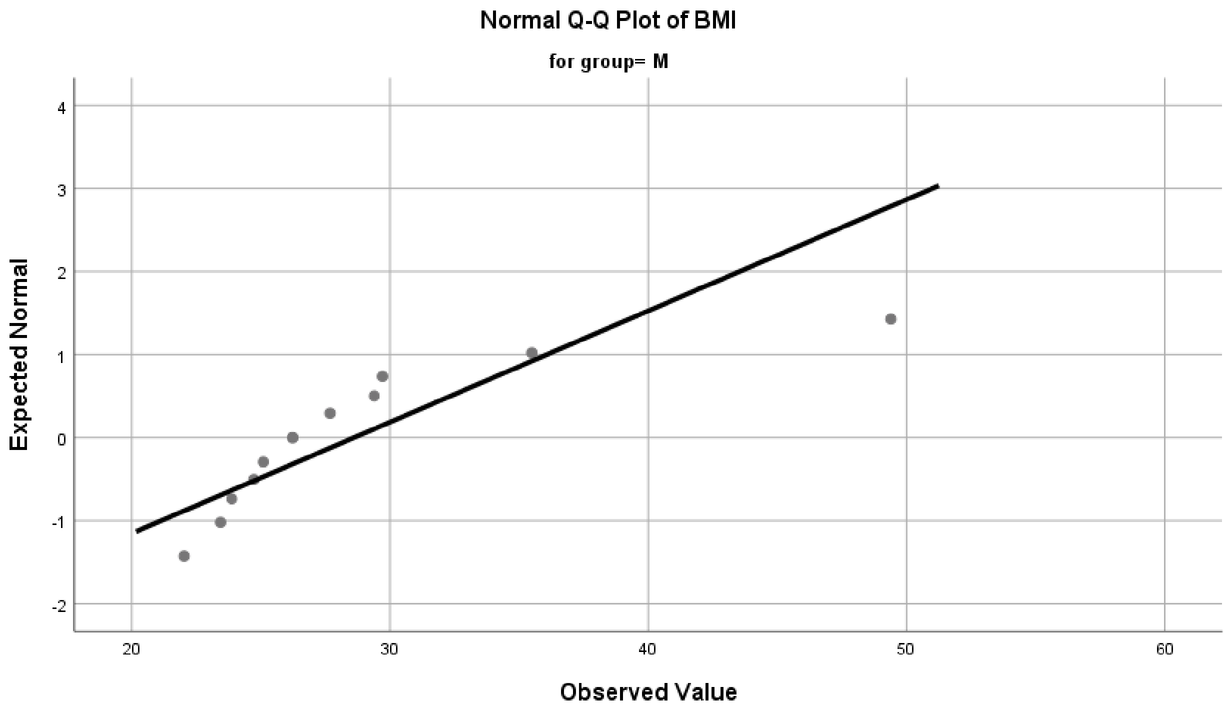
Normal Q-Q Plot of BMI
for group= M
Expected Normal
Observed Value
4
3
2
1
0
-1
-2
20
30
40
50
60

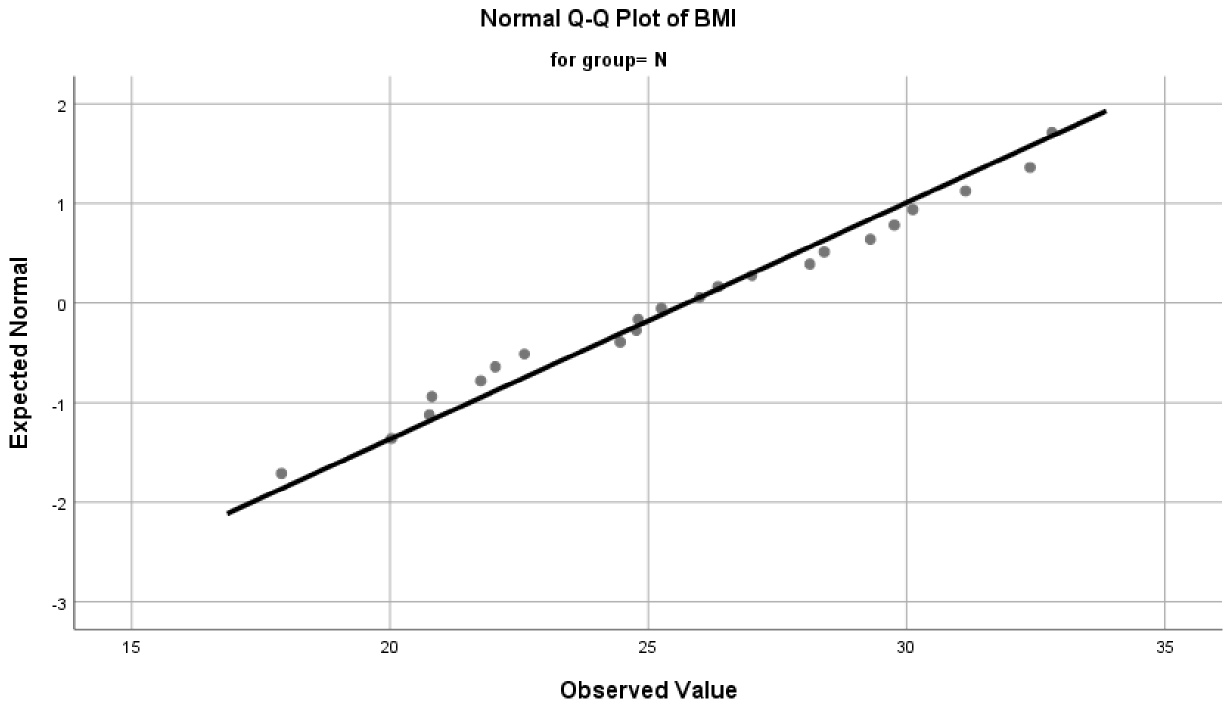
Normal Q-Q Plot of BMI
for group= N
Expected Normal
Observed Value
2
1
0
-1
-2
-3
15
20
25
30
35

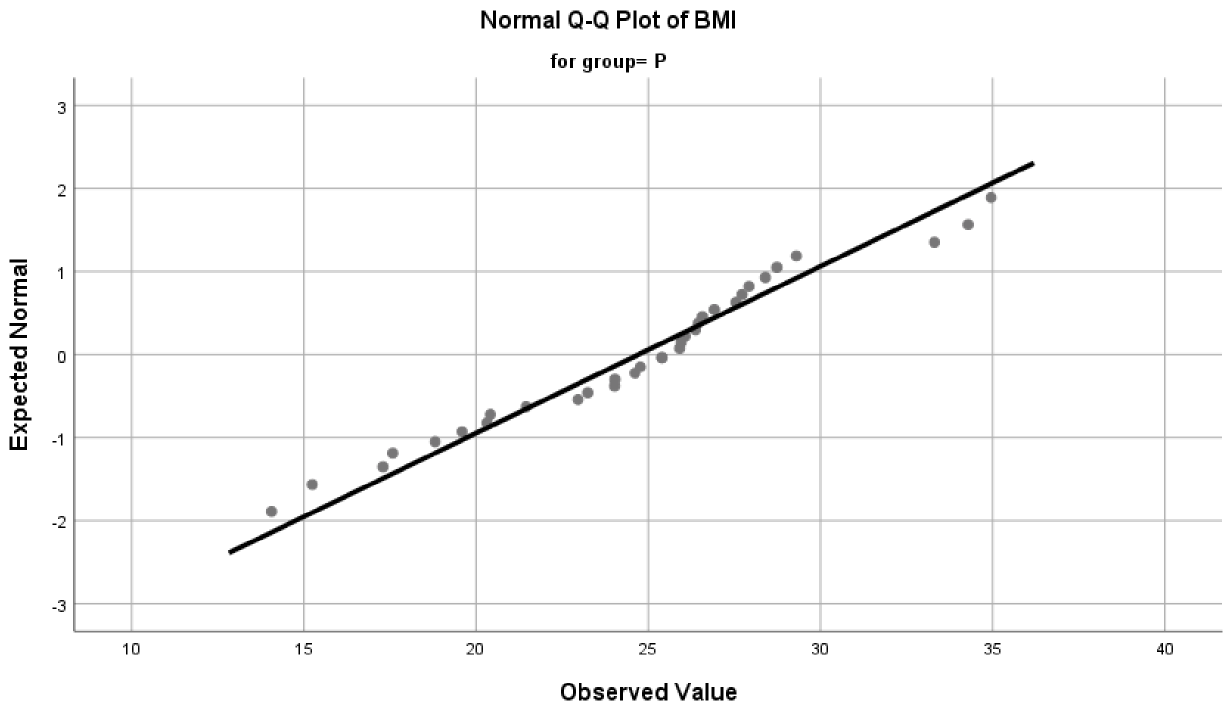
Normal Q-Q Plot of BMI
for group= P
Expected Normal
Observed Value
3
2
1
0
-1
-2
-3
10
15
20
25
30
35
40

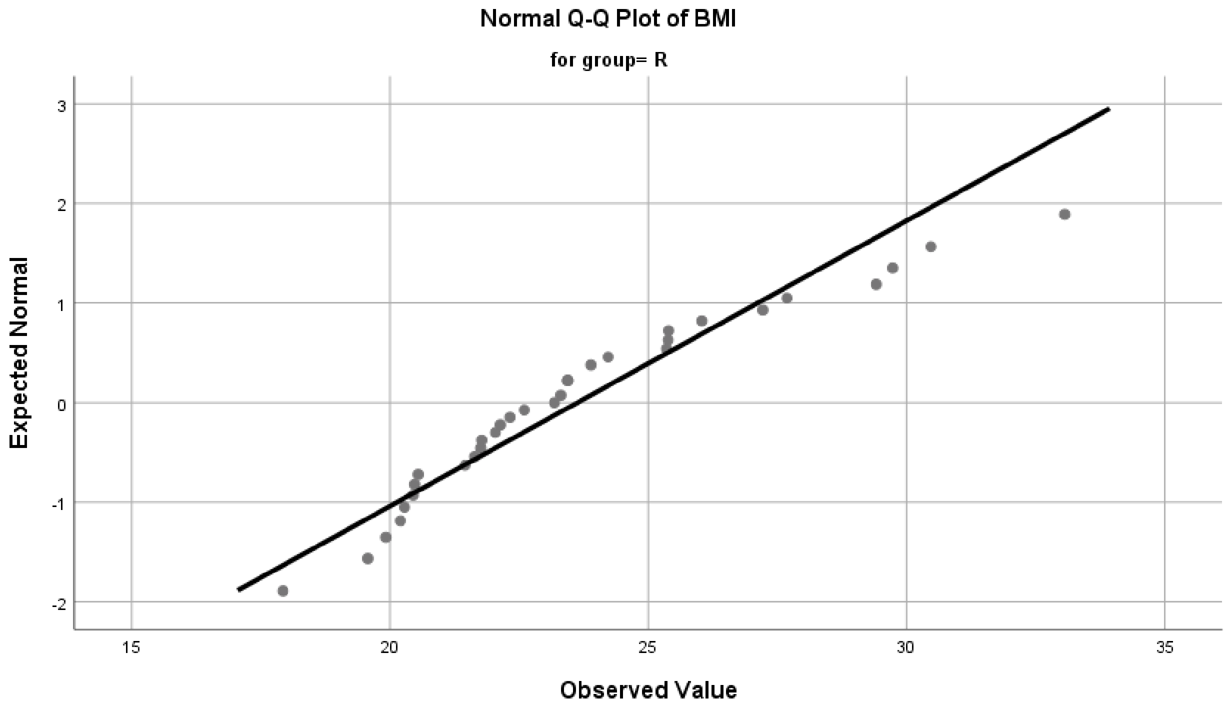
Normal Q-Q Plot of BMI
for group= R
Expected Normal
Observed Value
3
2
1
0
-1
-2
15
20
25
30
35

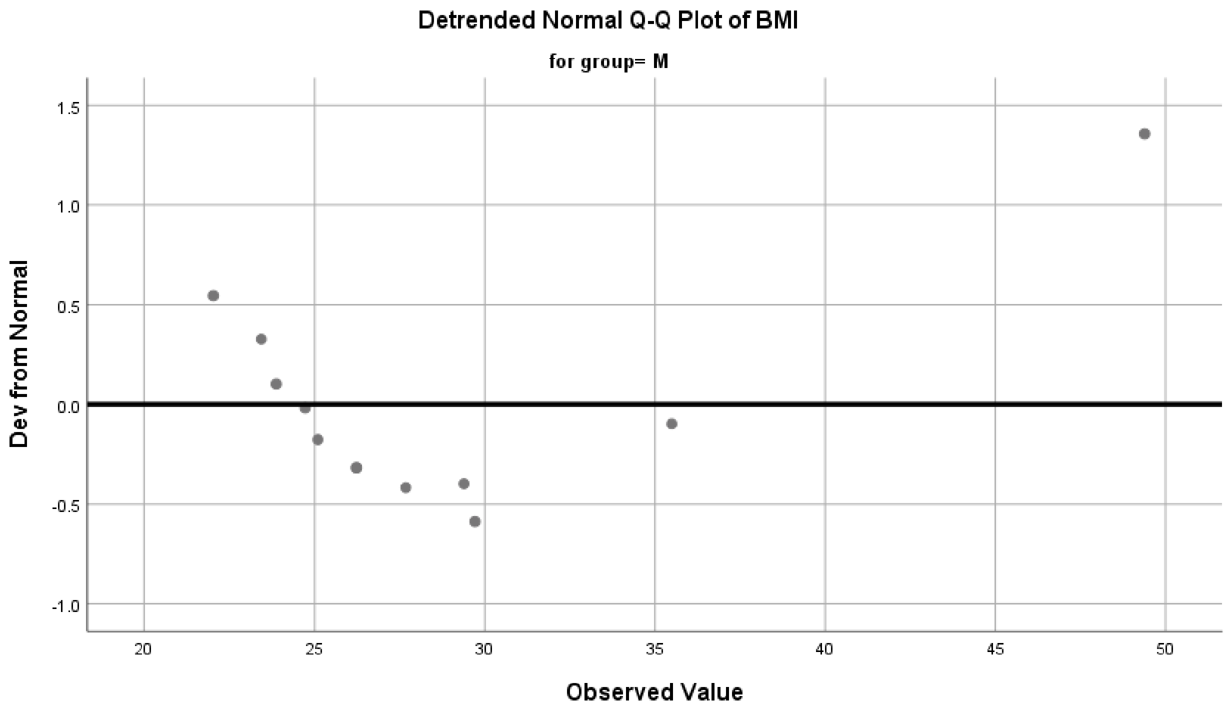

Detrended Normal Q-Q Plot of BMI
for group= M
Dev from Normal
1.5
1.0
0.5
0.0
-0.5
-1.0
20
25
30
35
40
45
50
Observed Value

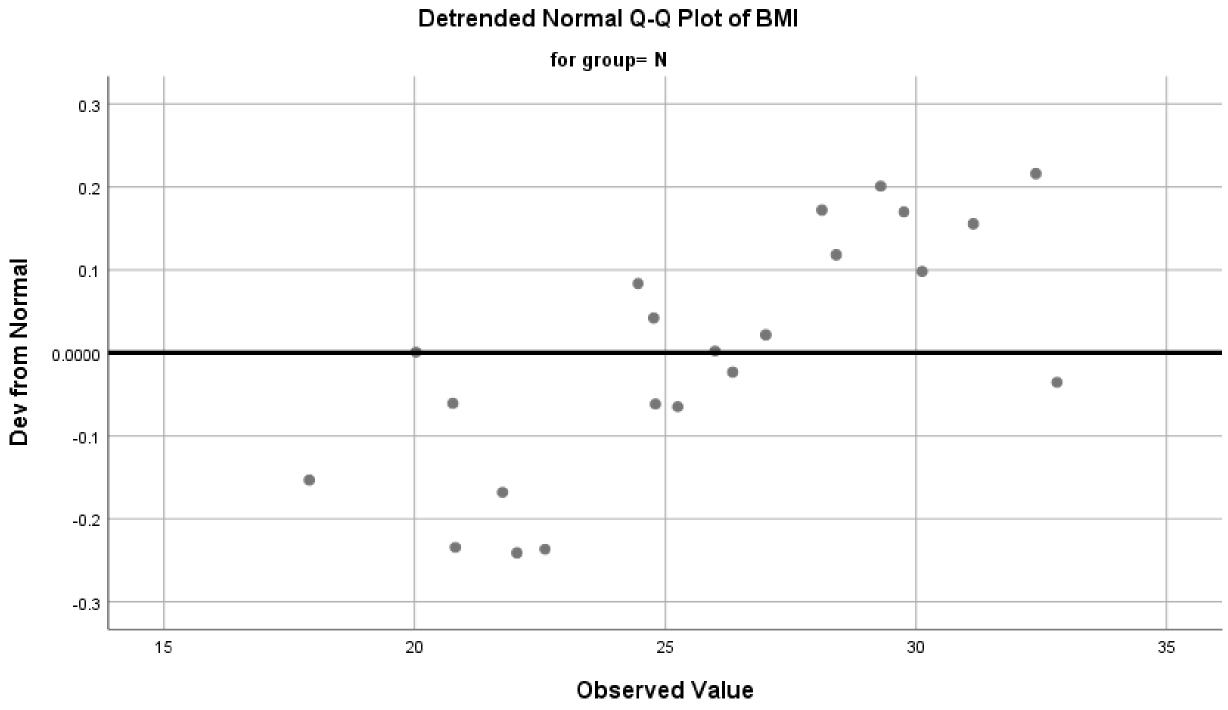

Detrended Normal Q-Q Plot of BMI
for group= N
Dev from Normal
0.3
0.2
0.1
0.0000
-0.1
-0.2
-0.3
15
20
25
30
35
Observed Value

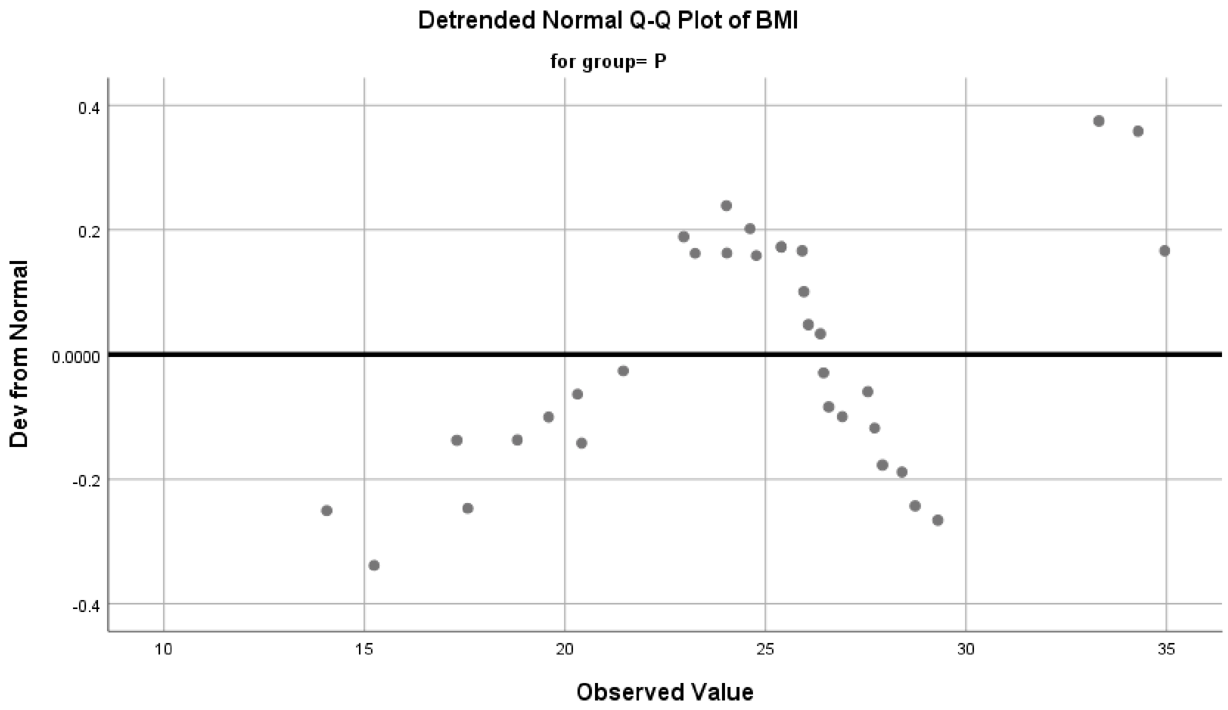
Detrended Normal Q-Q Plot of BMI
for group= P
Dev from Normal
0.4
0.2
0.0000
-0.2
-0.4
10
15
20
25
30
35
Observed Value

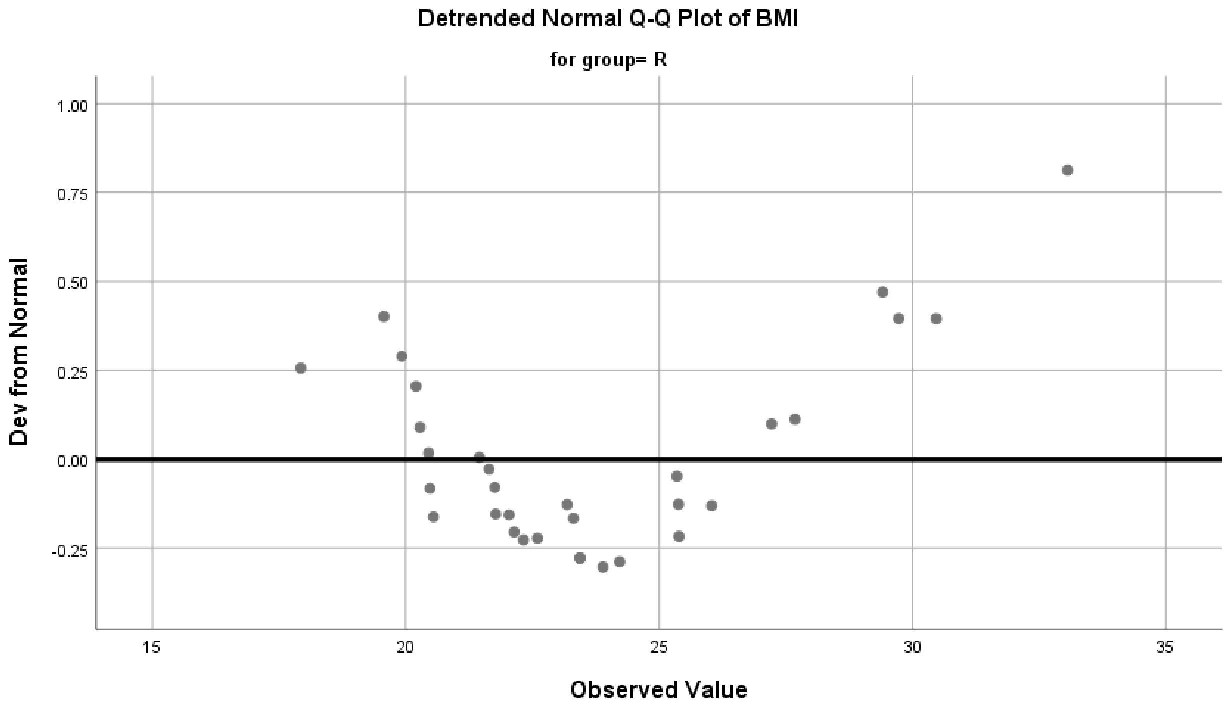
Detrended Normal Q-Q Plot of BMI
for group= R
Dev from Normal
1.00
0.75
0.50
0.25
0.00
-0.25
15
20
25
30
35
Observed Value

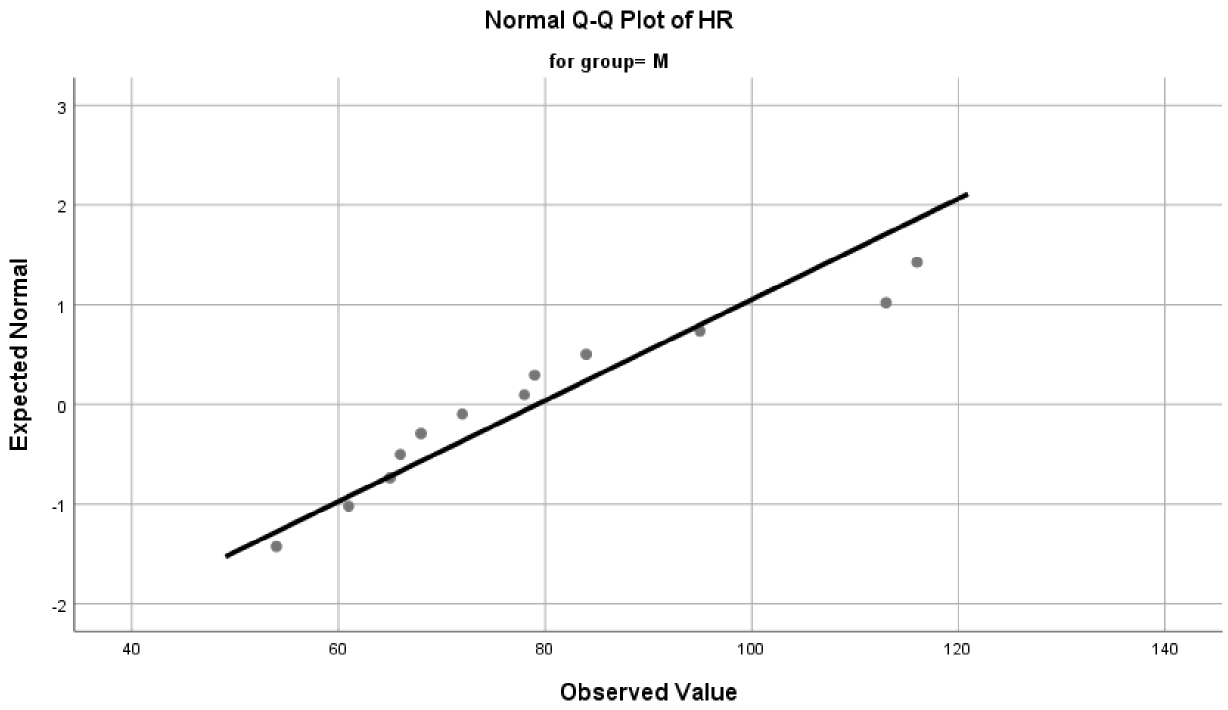
Normal Q-Q Plot of HR
for group= M
Expected Normal
Observed Value
3
2
1
0
-1
-2
40
60
80
100
120
140

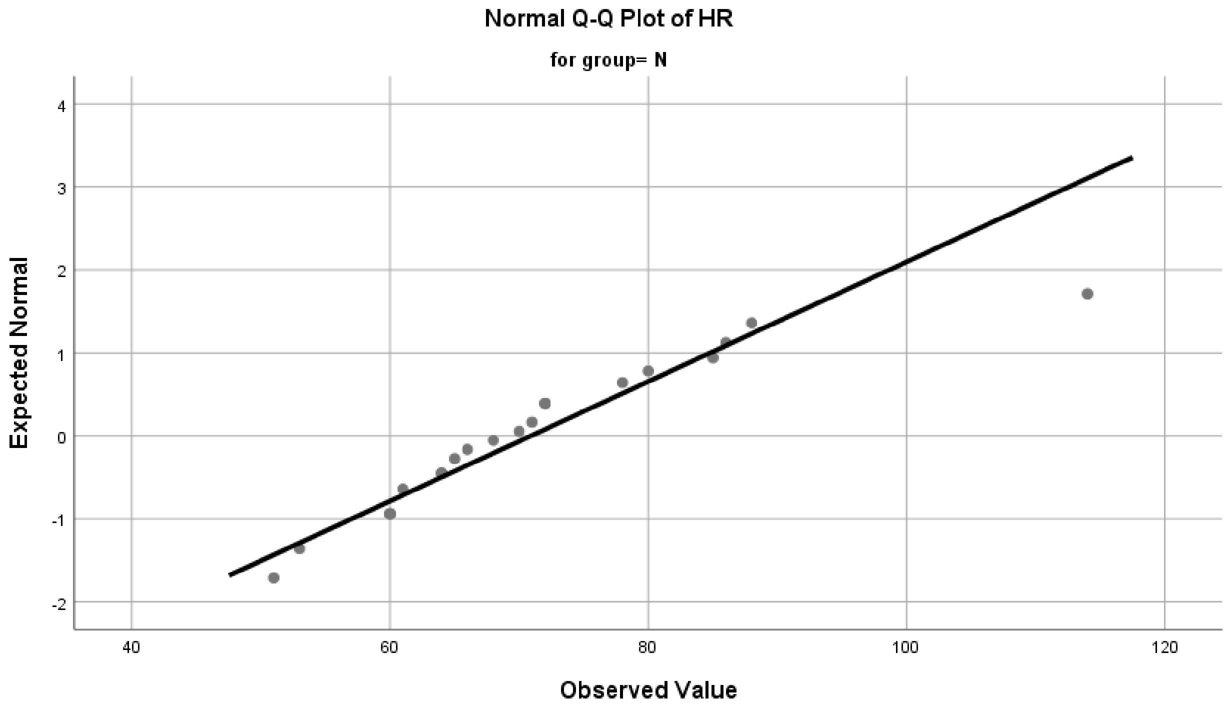
Normal Q-Q Plot of HR
for group= N
Expected Normal
Observed Value
4
3
2
1
0
-1
-2
40
60
80
100
120

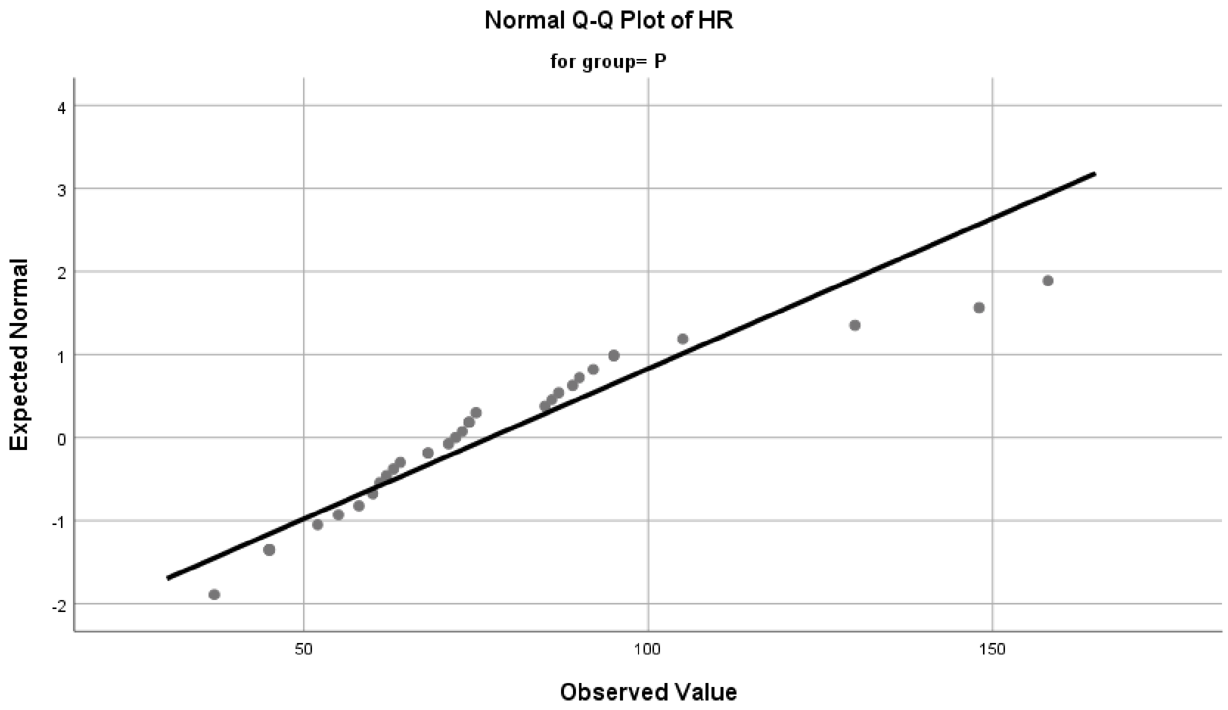

Normal Q-Q Plot of HR
for group= P
Expected Normal
Observed Value
4
3
2
1
0
-1
-2
50
100
150

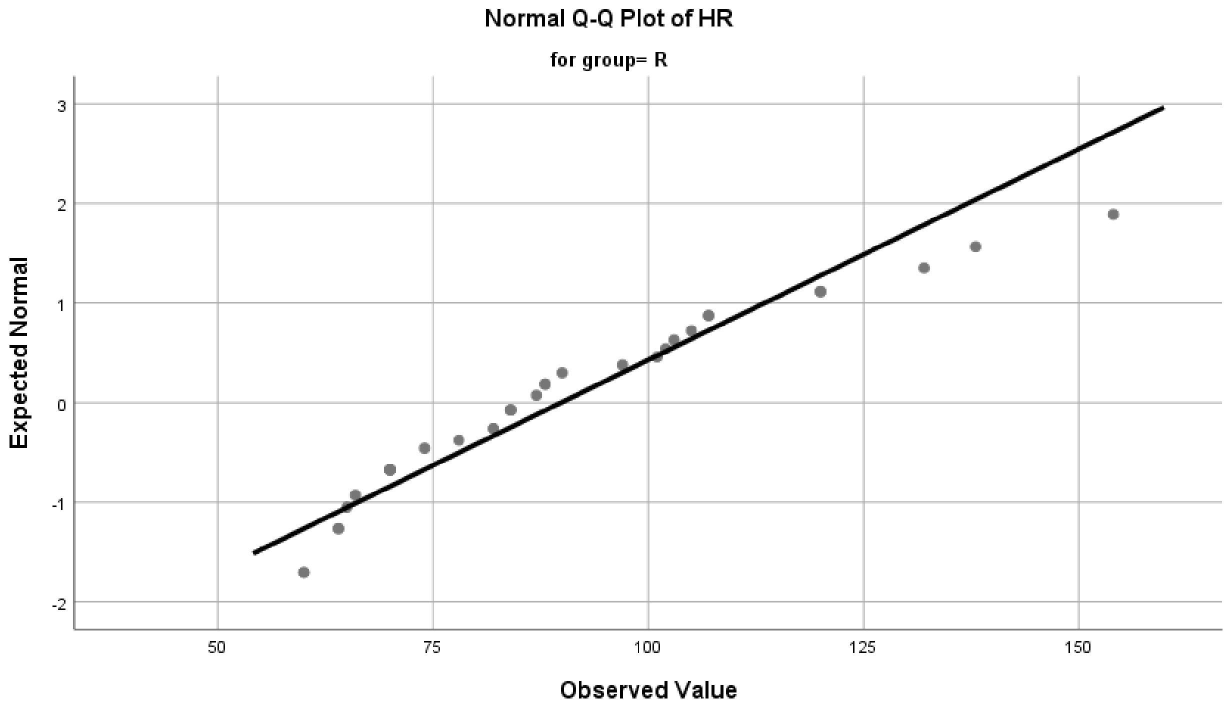

Normal Q-Q Plot of HR
for group= R
Expected Normal
Observed Value
3
2
1
0
-1
-2
50
75
100
125
150

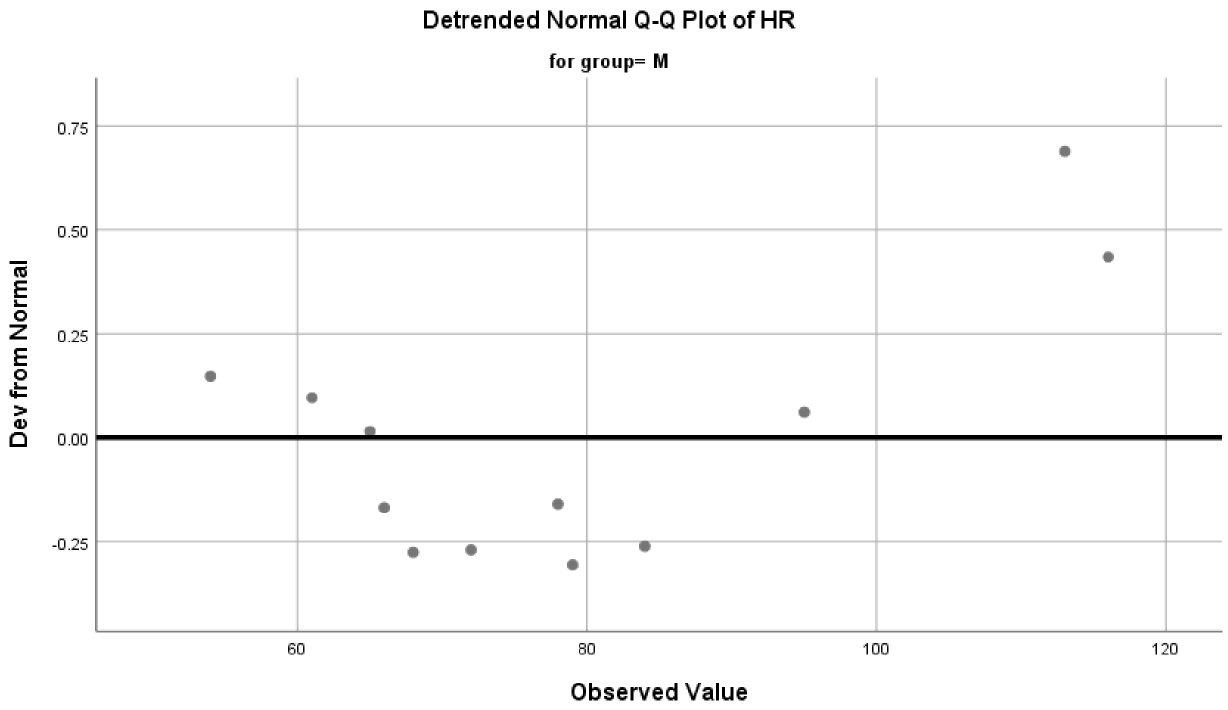
Detrended Normal Q-Q Plot of HR
for group= M
Dev from Normal
0.75
0.50
0.25
0.00
-0.25
60
80
100
120
Observed Value

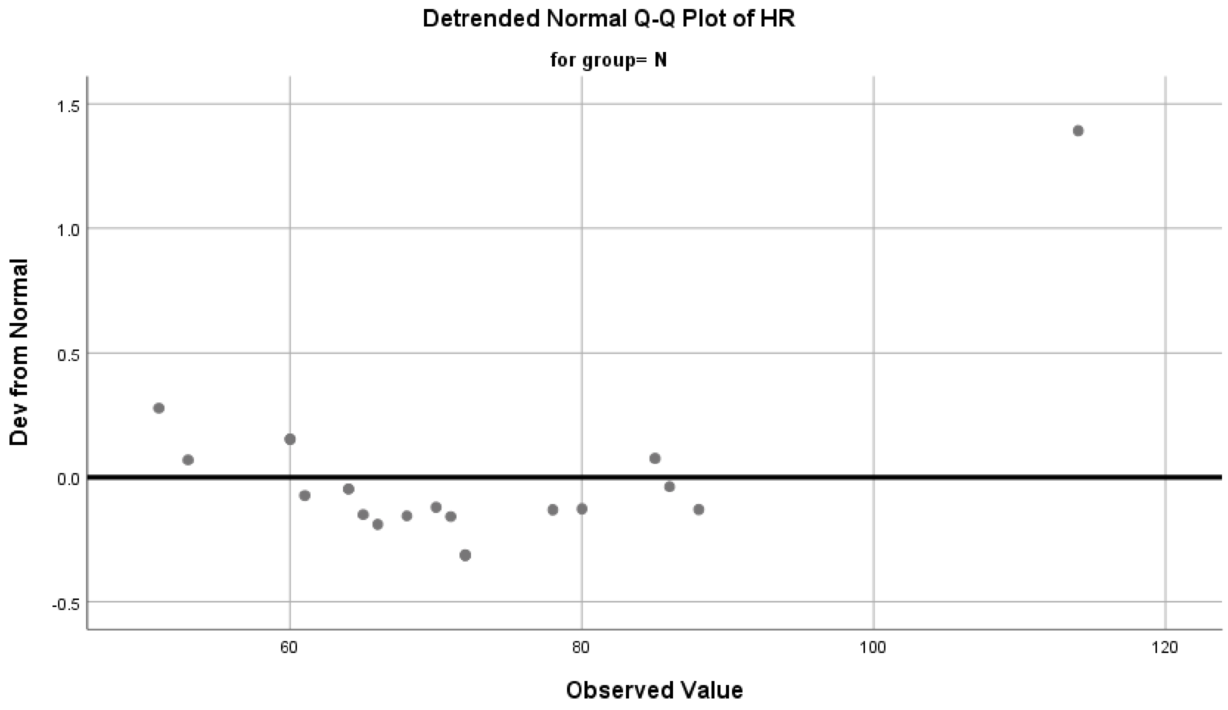
Detrended Normal Q-Q Plot of HR
for group= N
Dev from Normal
1.5
1.0
0.5
0.0
-0.5
60
80
100
120
Observed Value

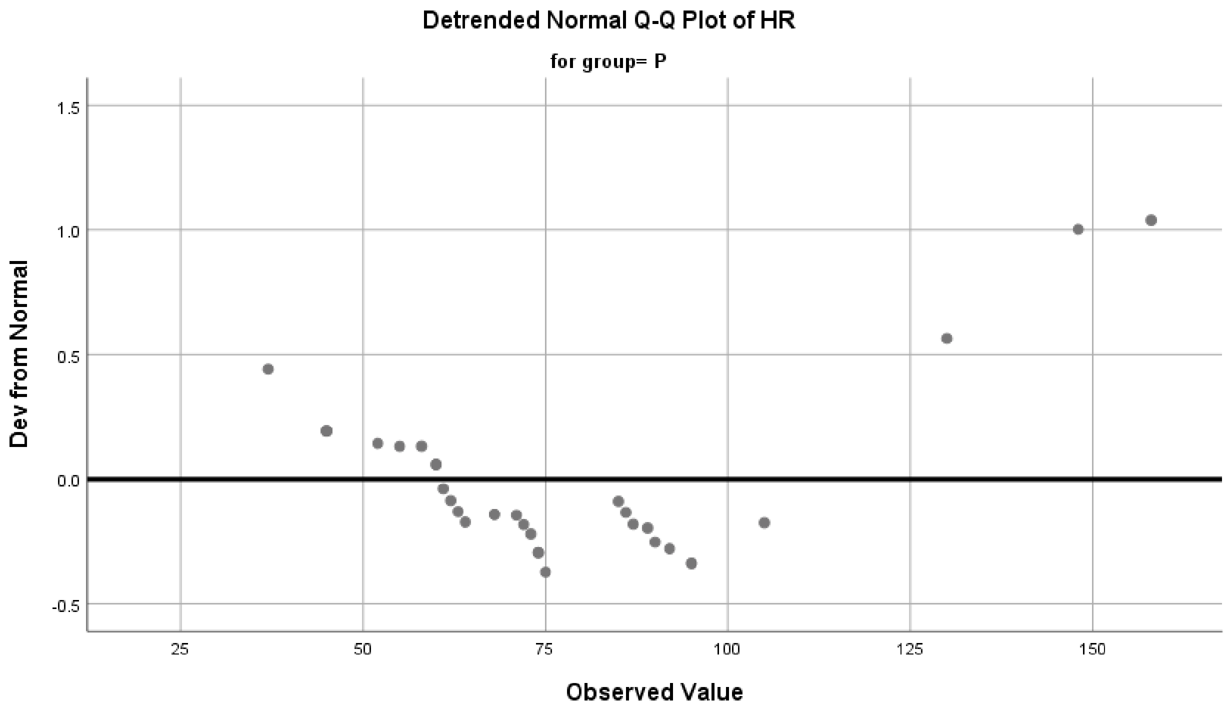

Detrended Normal Q-Q Plot of HR
for group= P
Dev from Normal
Observed Value
1.5
1.0
0.5
0.0
-0.5
25
50
75
100
125
150

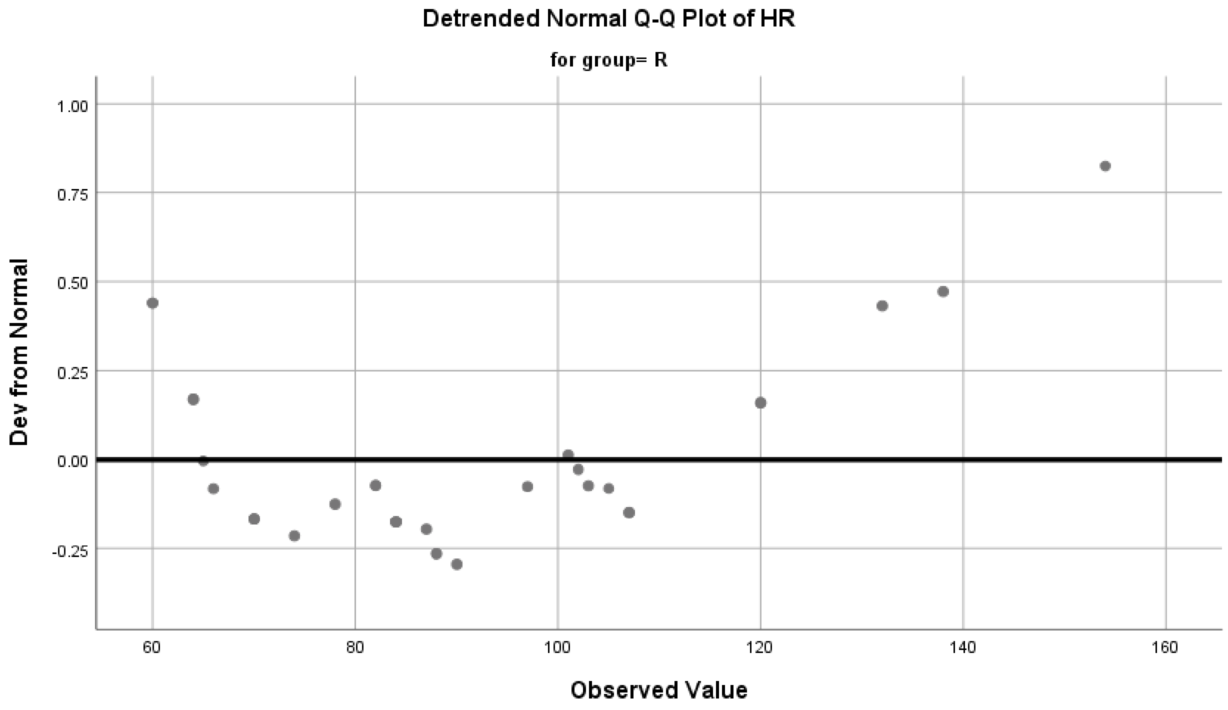

Detrended Normal Q-Q Plot of HR
for group= R
Dev from Normal
Observed Value
1.00
0.75
0.50
0.25
0.00
-0.25
60
80
100
120
140
160

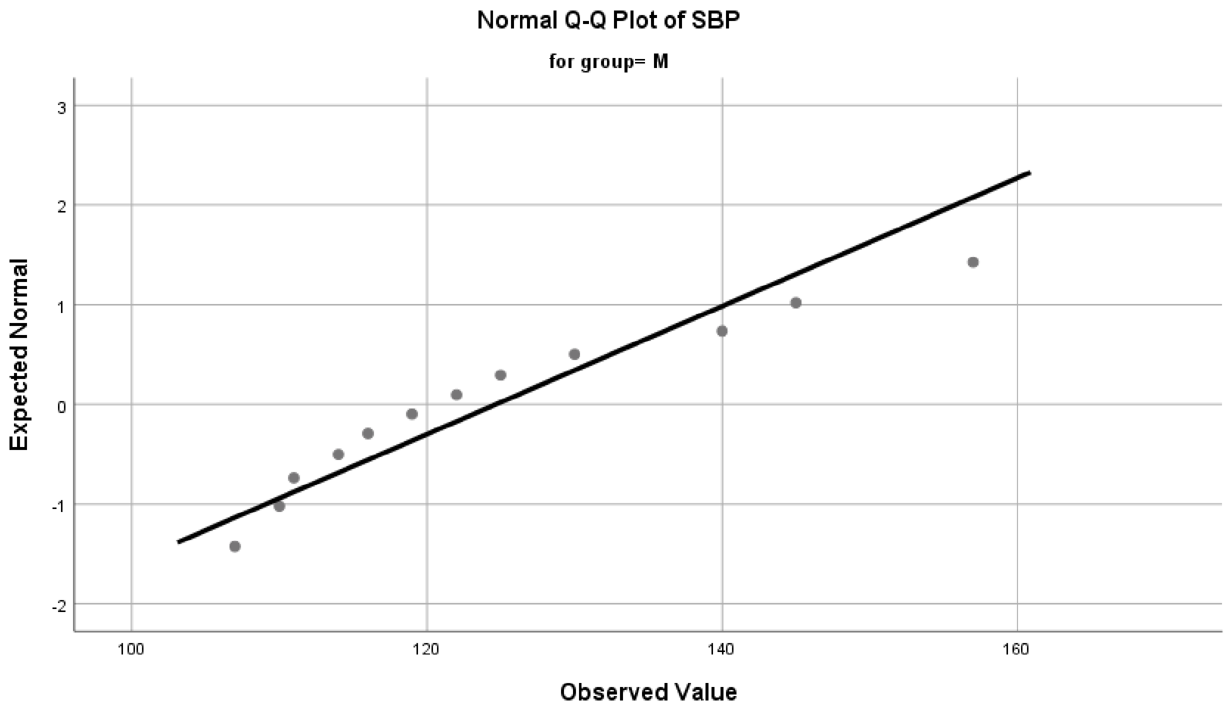
Normal Q-Q Plot of SBP
for group= M
Expected Normal
Observed Value
3
2
1
0
-1
-2
100
120
140
160

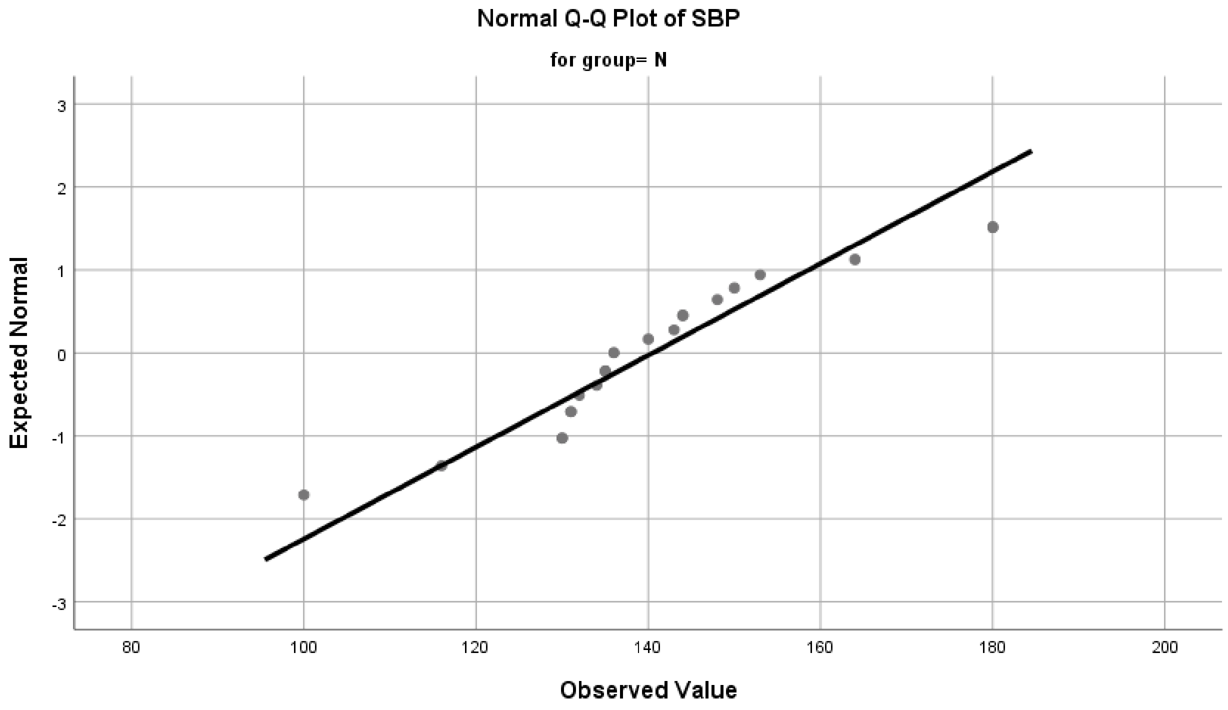
Normal Q-Q Plot of SBP
for group= N
Expected Normal
Observed Value
3
2
1
0
-1
-2
-3
80
100
120
140
160
180
200

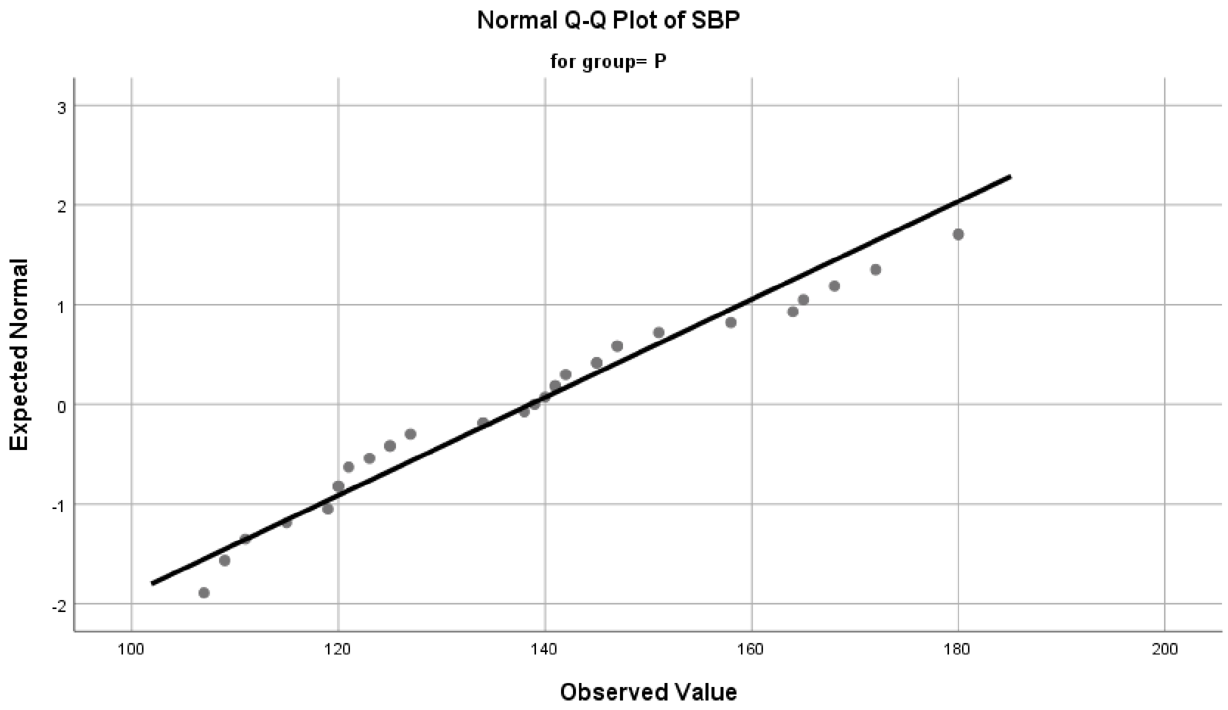

Normal Q-Q Plot of SBP
for group= P
Expected Normal
Observed Value
3
2
1
0
-1
-2
100
120
140
160
180
200

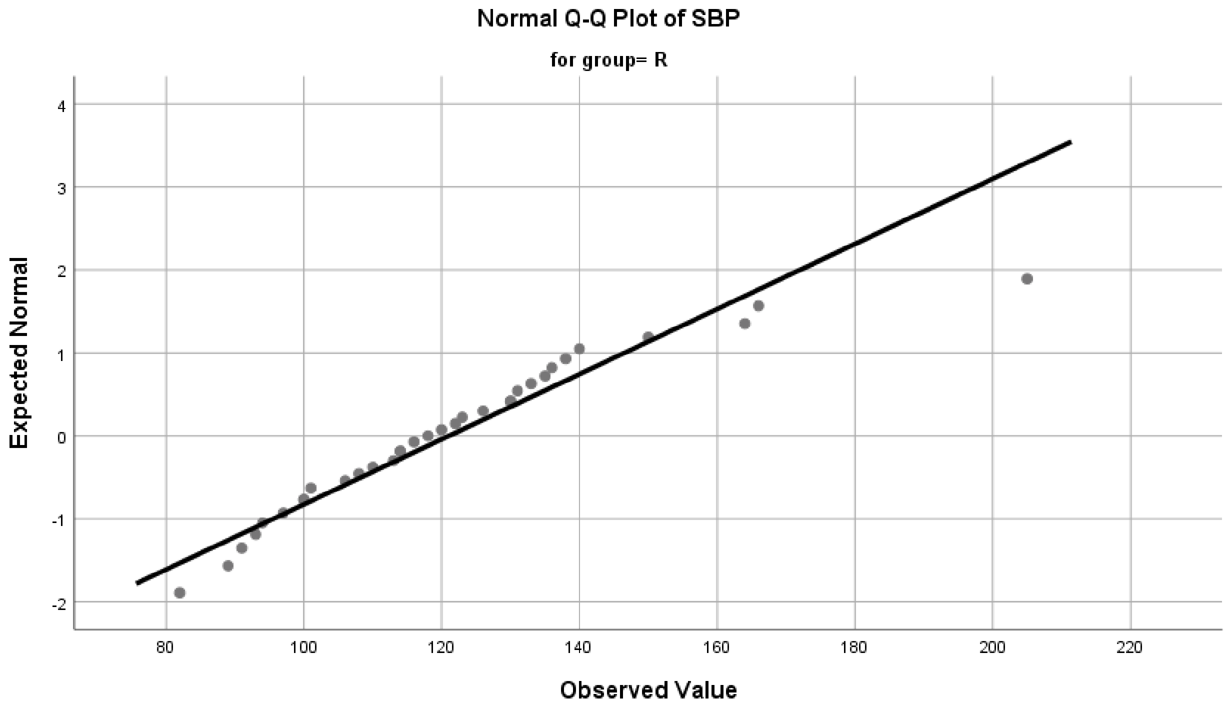

Normal Q-Q Plot of SBP
for group= R
Expected Normal
Observed Value
4
3
2
1
0
-1
-2
80
100
120
140
160
180
200
220

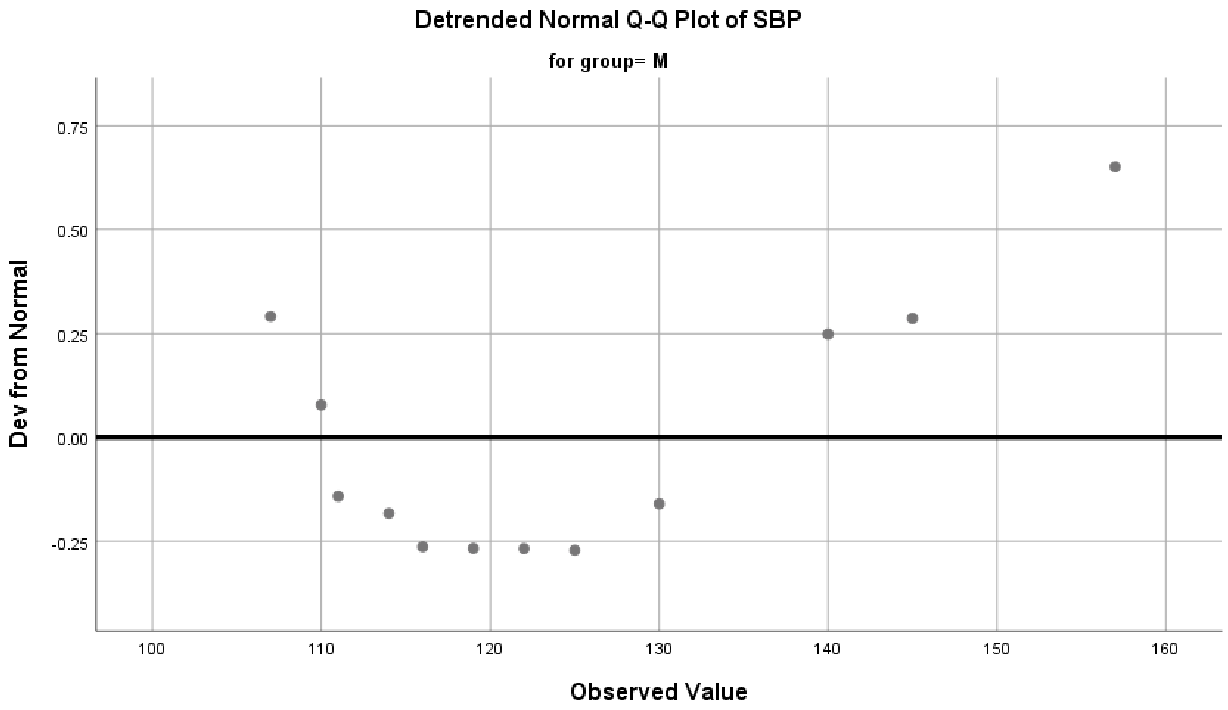

Detrended Normal Q-Q Plot of SBP
for group= M
Dev from Normal
0.75
0.50
0.25
0.00
-0.25
100
110
120
130
140
150
160
Observed Value

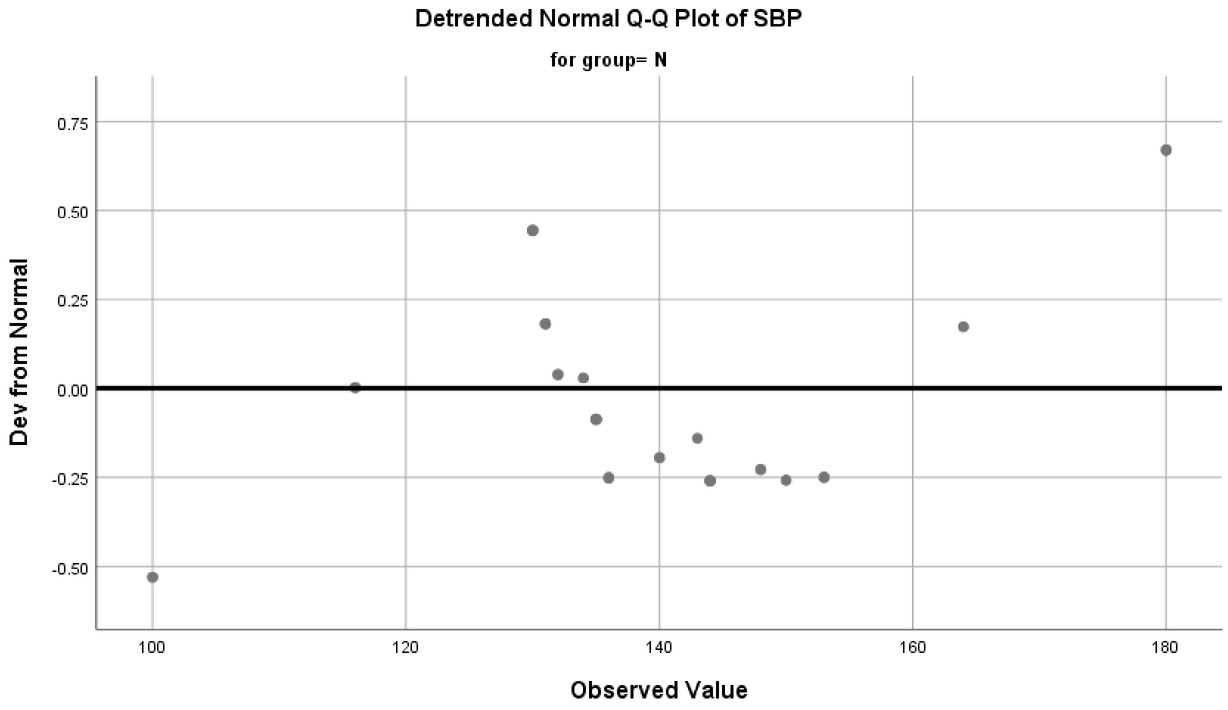

Detrended Normal Q-Q Plot of SBP
for group= N
Dev from Normal
0.75
0.50
0.25
0.00
-0.25
-0.50
100
120
140
160
180
Observed Value

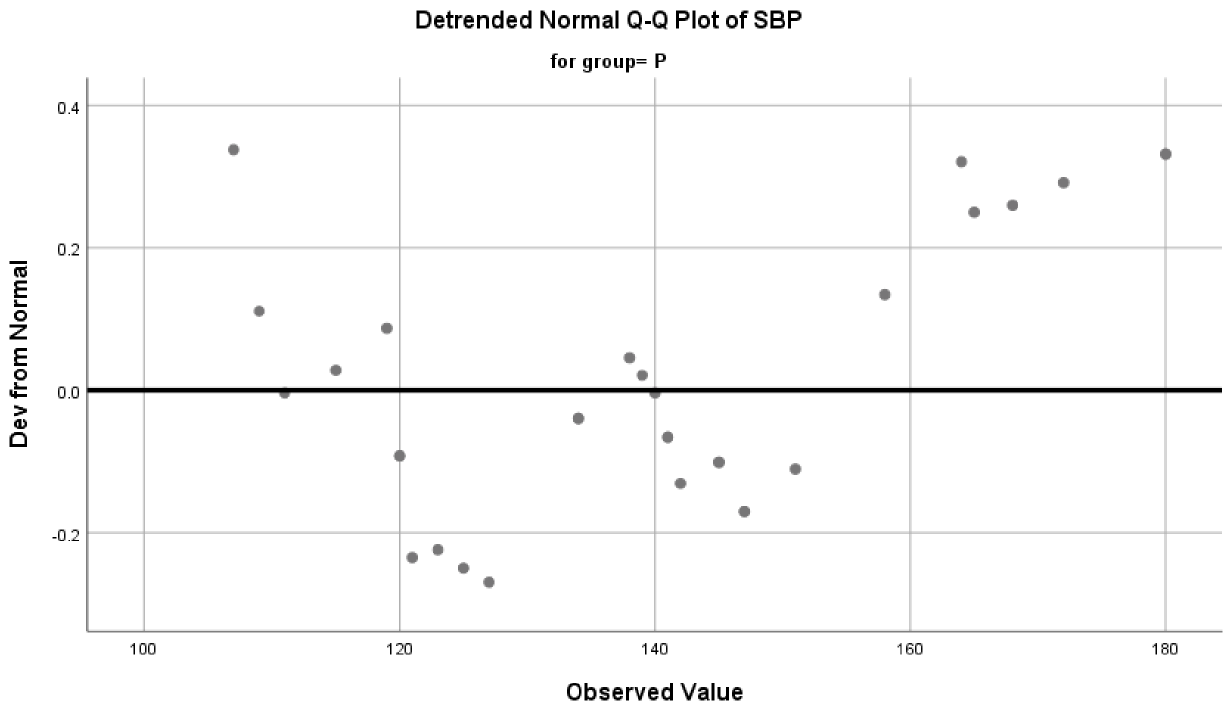
Detrended Normal Q-Q Plot of SBP
for group= P
Dev from Normal
0.4
0.2
0.0
-0.2
100
120
140
160
180
Observed Value

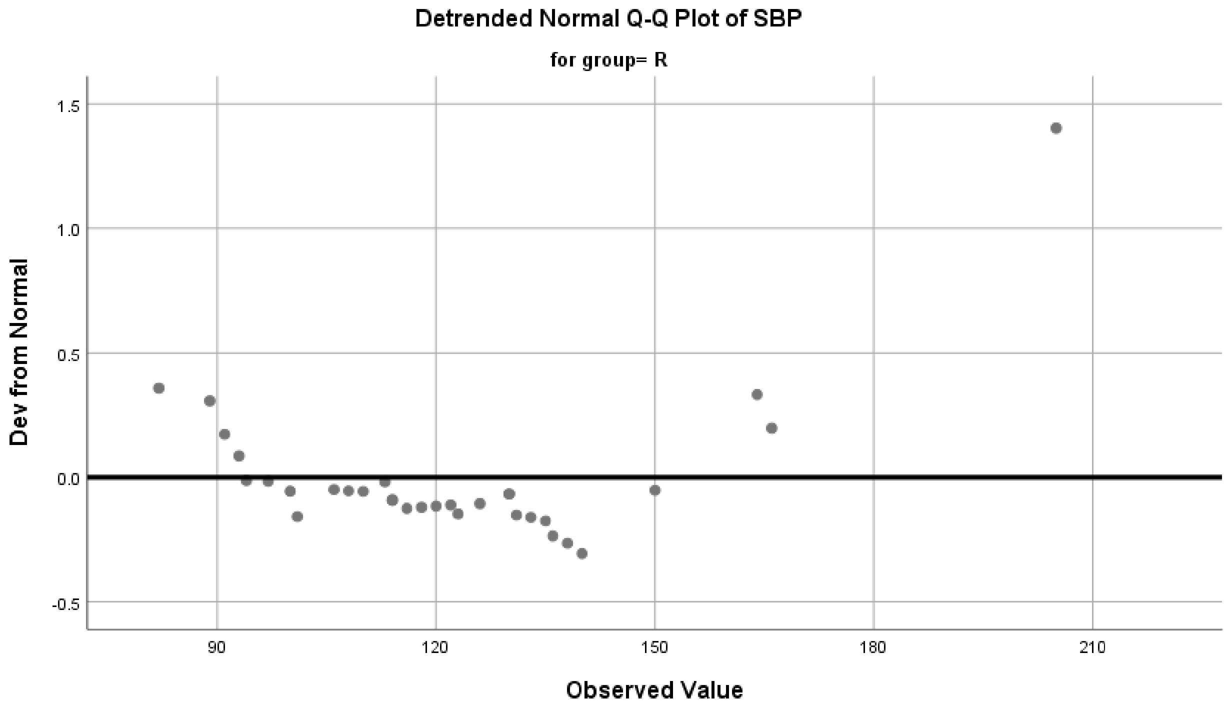
Detrended Normal Q-Q Plot of SBP
for group= R
Dev from Normal
1.5
1.0
0.5
0.0
-0.5
90
120
150
180
210
Observed Value

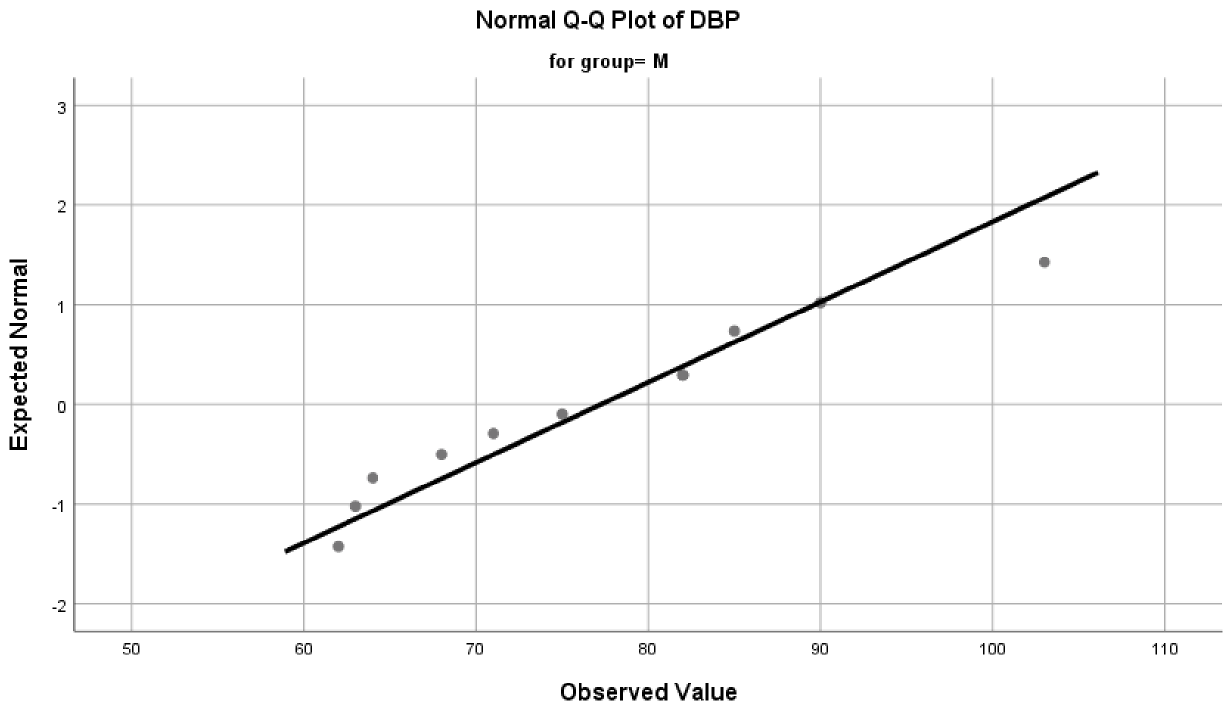
Normal Q-Q Plot of DBP
for group= M
Expected Normal
Observed Value
3
2
1
0
-1
-2
50
60
70
80
90
100
110

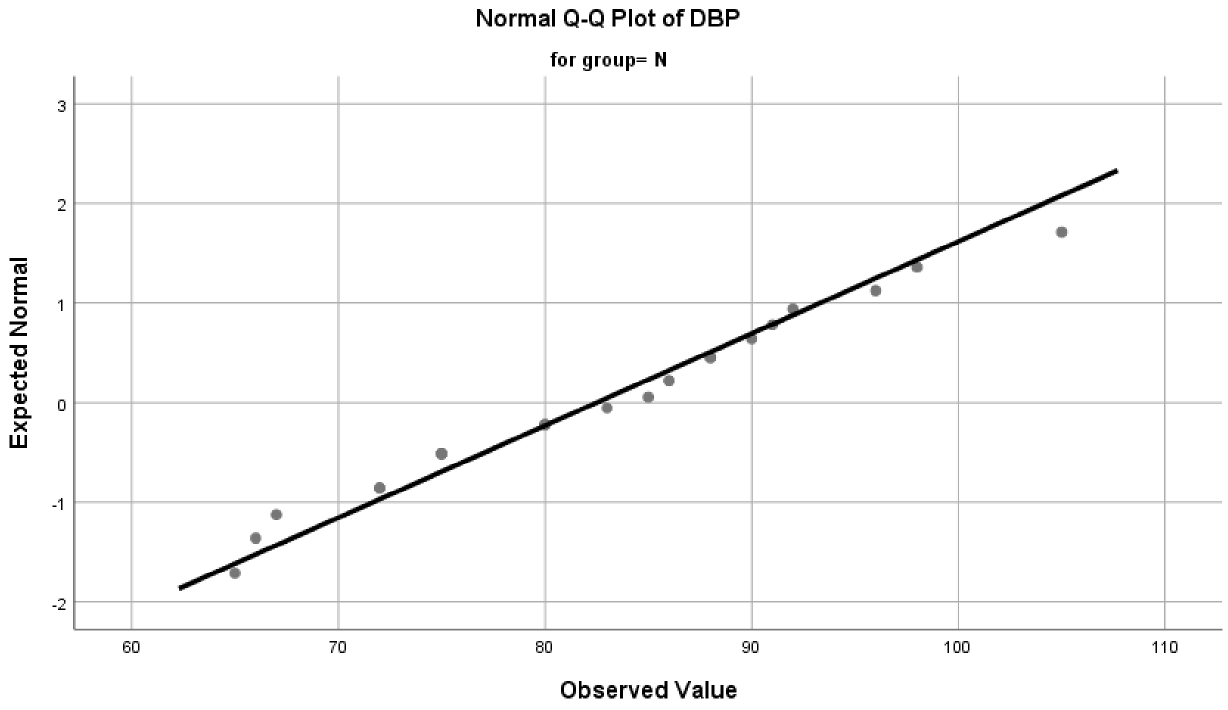
Normal Q-Q Plot of DBP
for group= N
Expected Normal
Observed Value
3
2
1
0
-1
-2
60
70
80
90
100
110

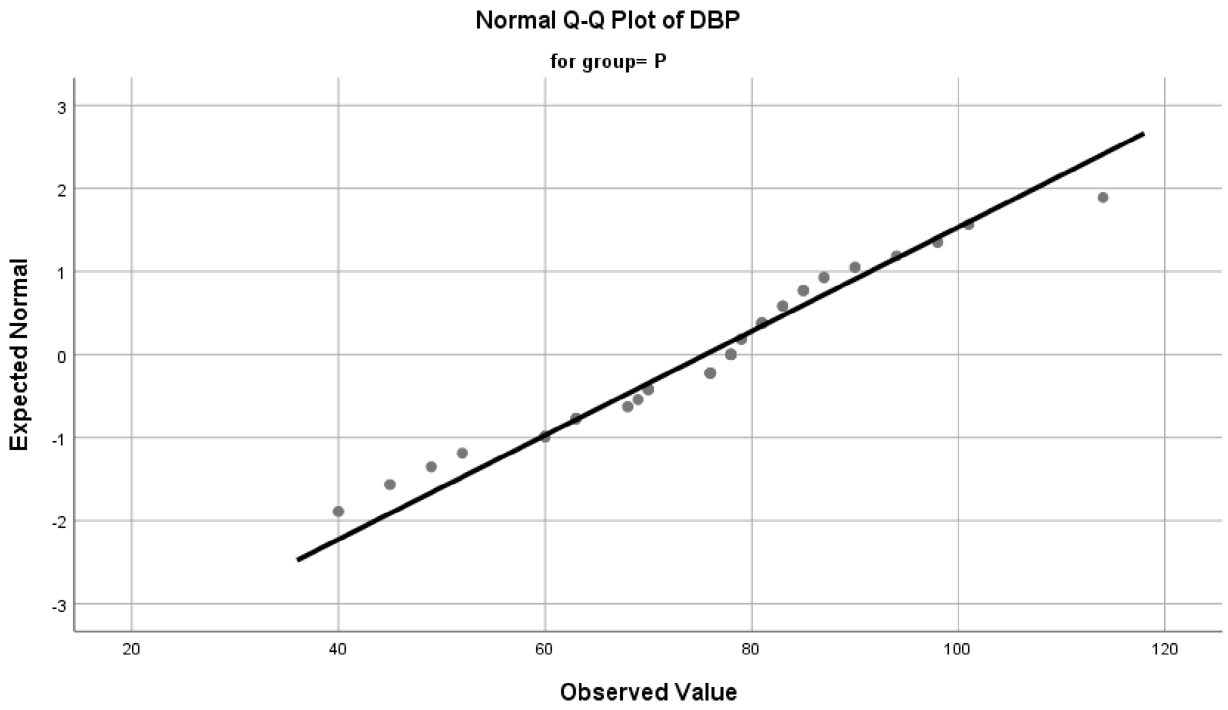
Normal Q-Q Plot of DBP
for group= P
Expected Normal
Observed Value
3
2
1
0
-1
-2
-3
20
40
60
80
100
120

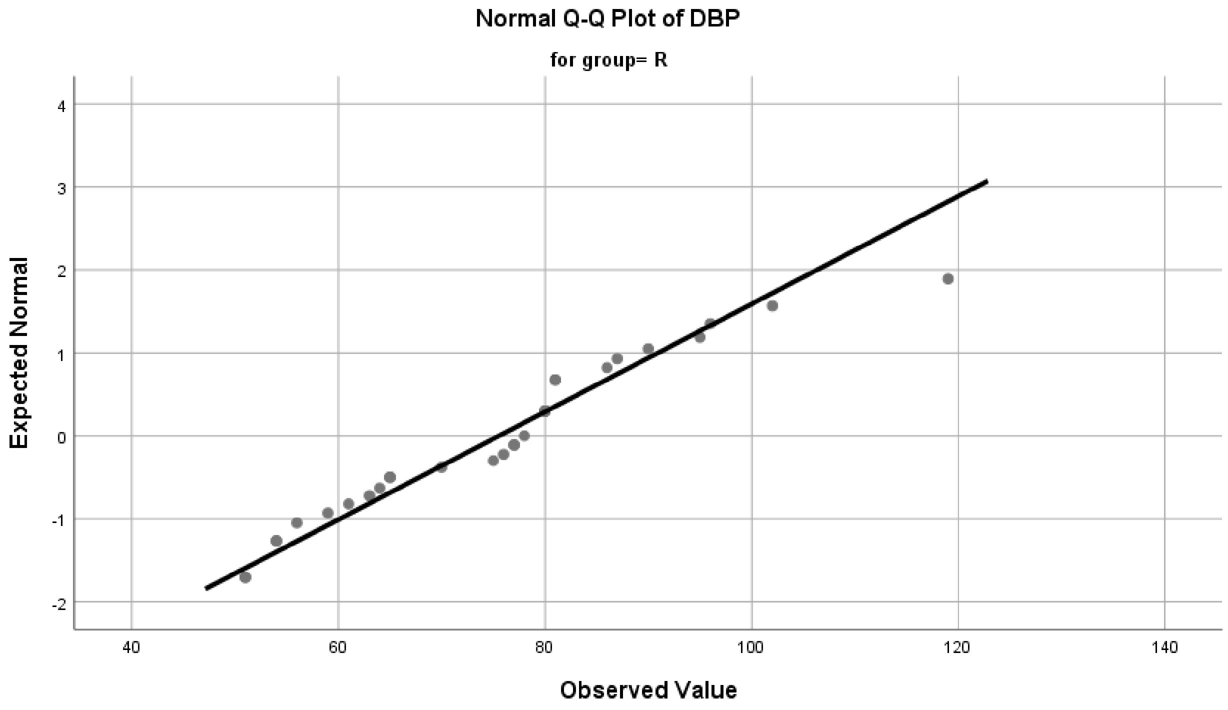
Normal Q-Q Plot of DBP
for group= R
Expected Normal
Observed Value
4
3
2
1
0
-1
-2
40
60
80
100
120
140

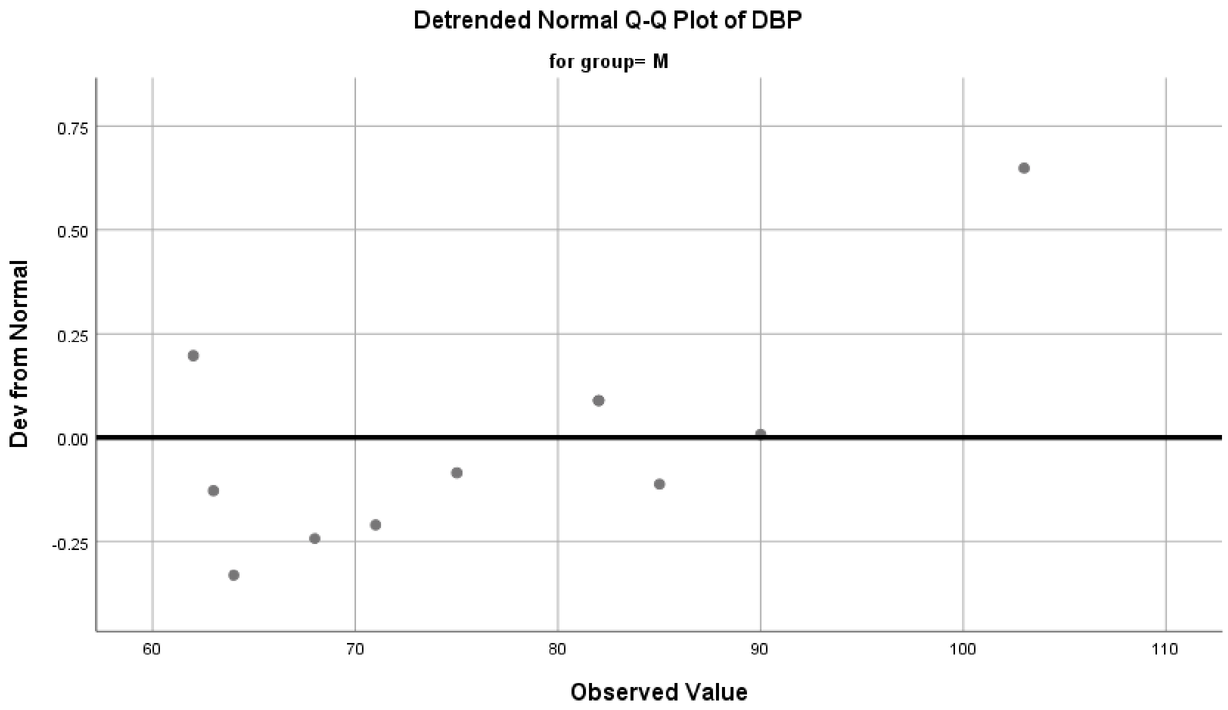

Detrended Normal Q-Q Plot of DBP
for group= M
Dev from Normal
0.75
0.50
0.25
0.00
-0.25
60
70
80
90
100
110
Observed Value

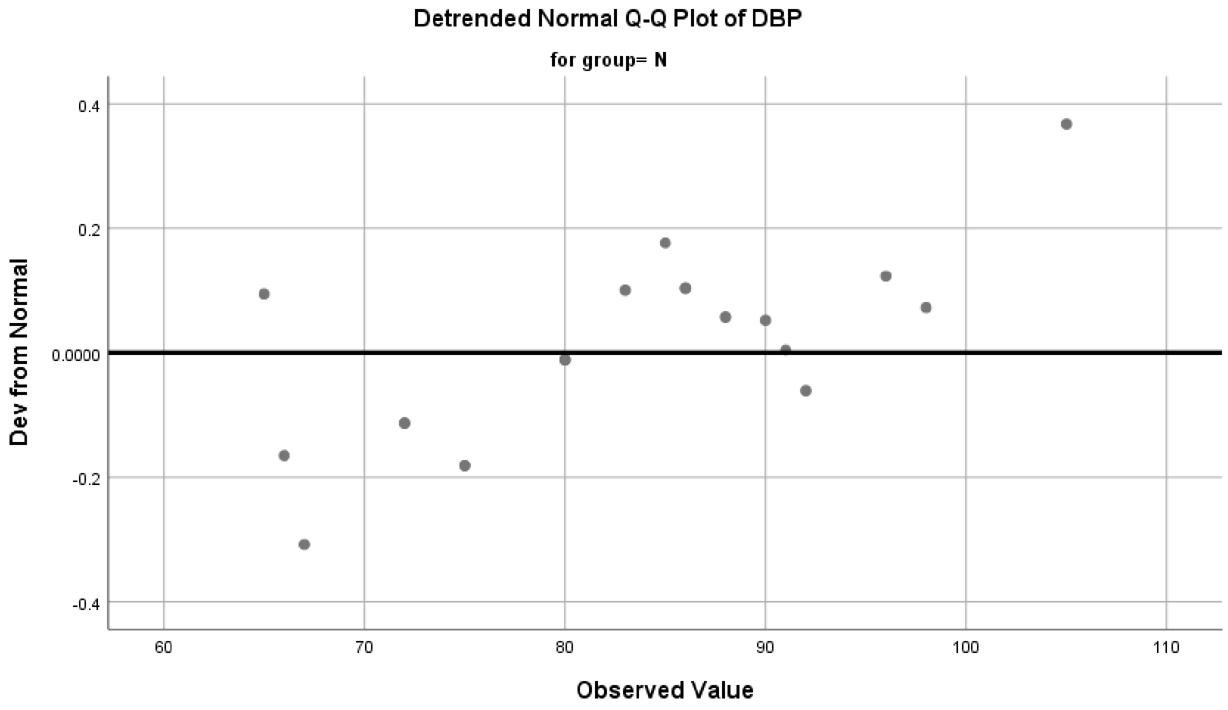

Detrended Normal Q-Q Plot of DBP
for group= N
Dev from Normal
0.4
0.2
0.0000
-0.2
-0.4
60
70
80
90
100
110
Observed Value

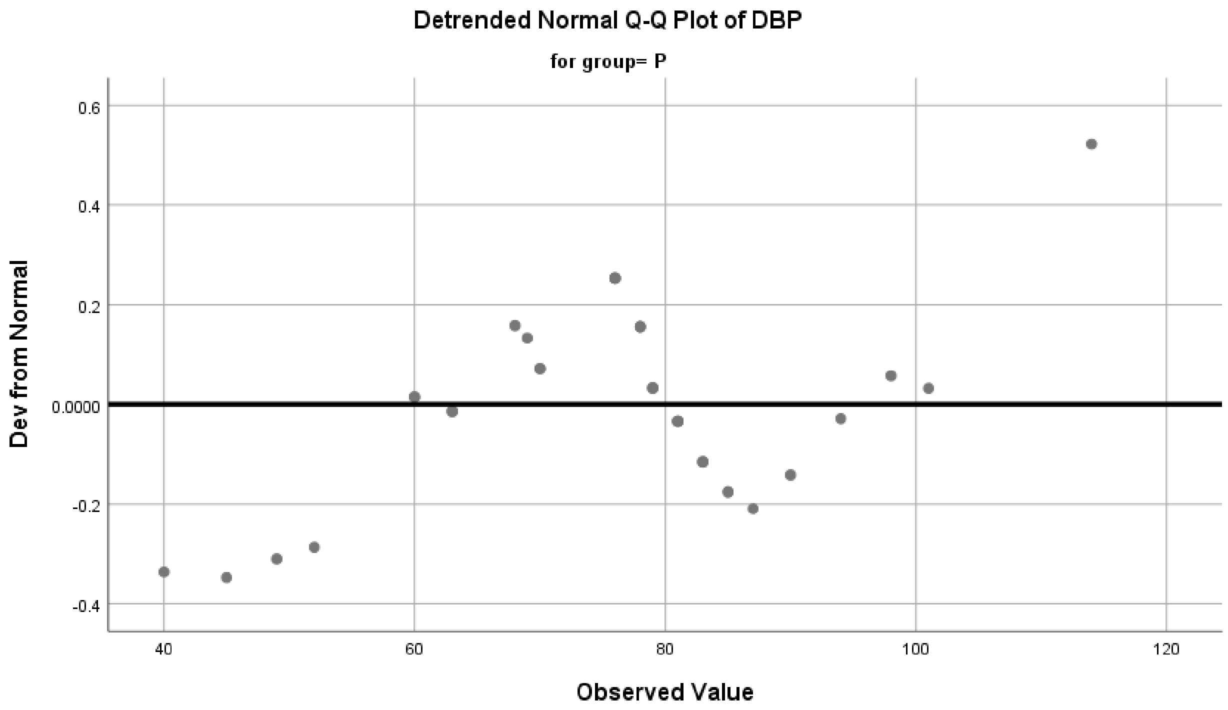
Detrended Normal Q-Q Plot of DBP
for group= P
Dev from Normal
Observed Value
0.6
0.4
0.2
0.0000
-0.2
-0.4
40
60
80
100
120

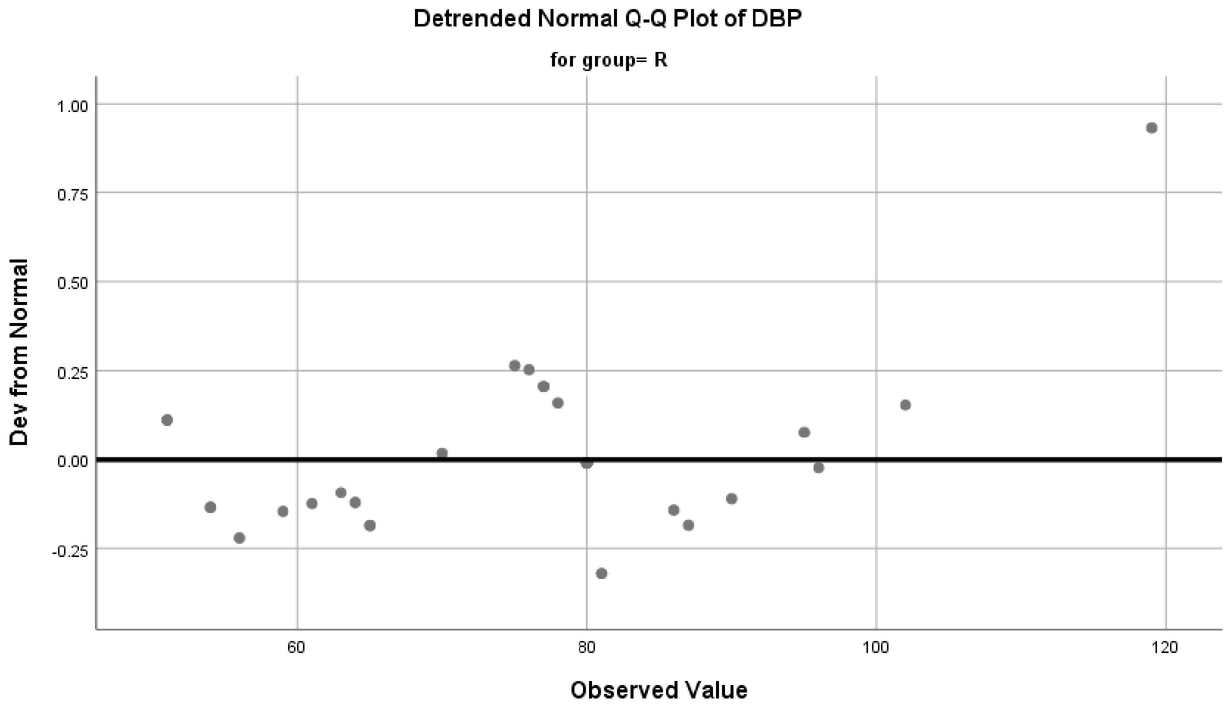
Detrended Normal Q-Q Plot of DBP
for group= R
Dev from Normal
Observed Value
1.00
0.75
0.50
0.25
0.00
-0.25
60
80
100
120

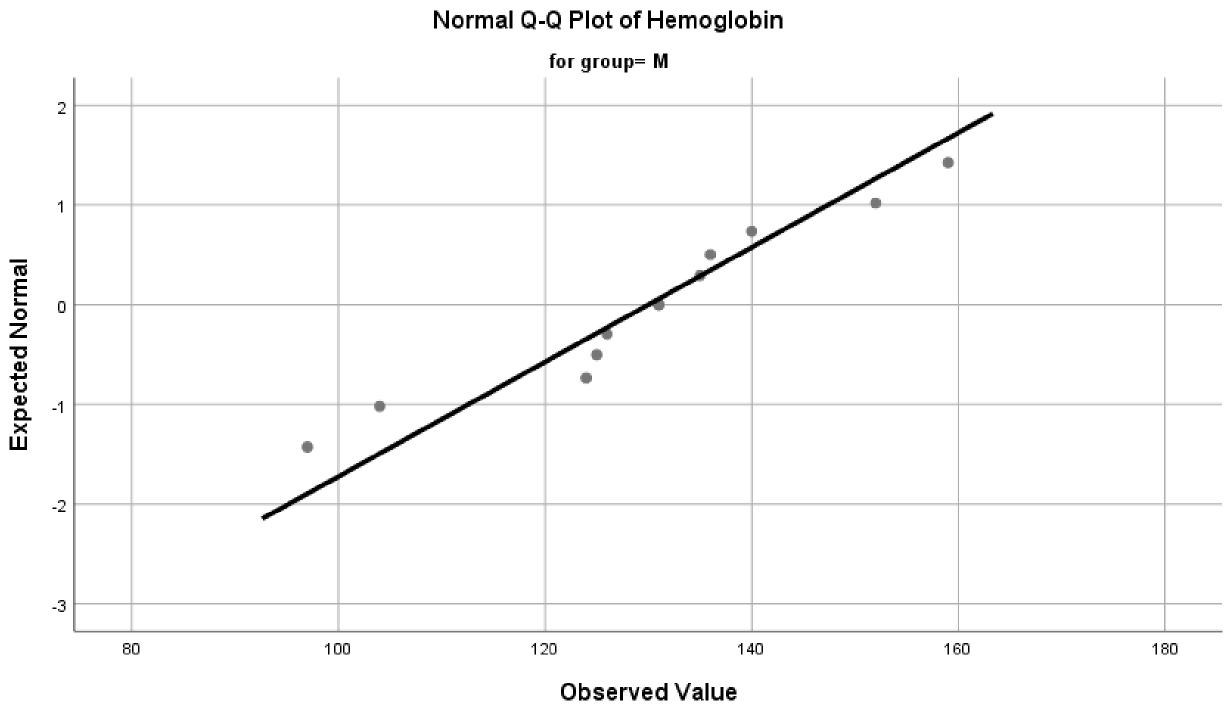
Normal Q-Q Plot of Hemoglobin
for group= M
Expected Normal
Observed Value
2
1
0
-1
-2
-3
80
100
120
140
160
180

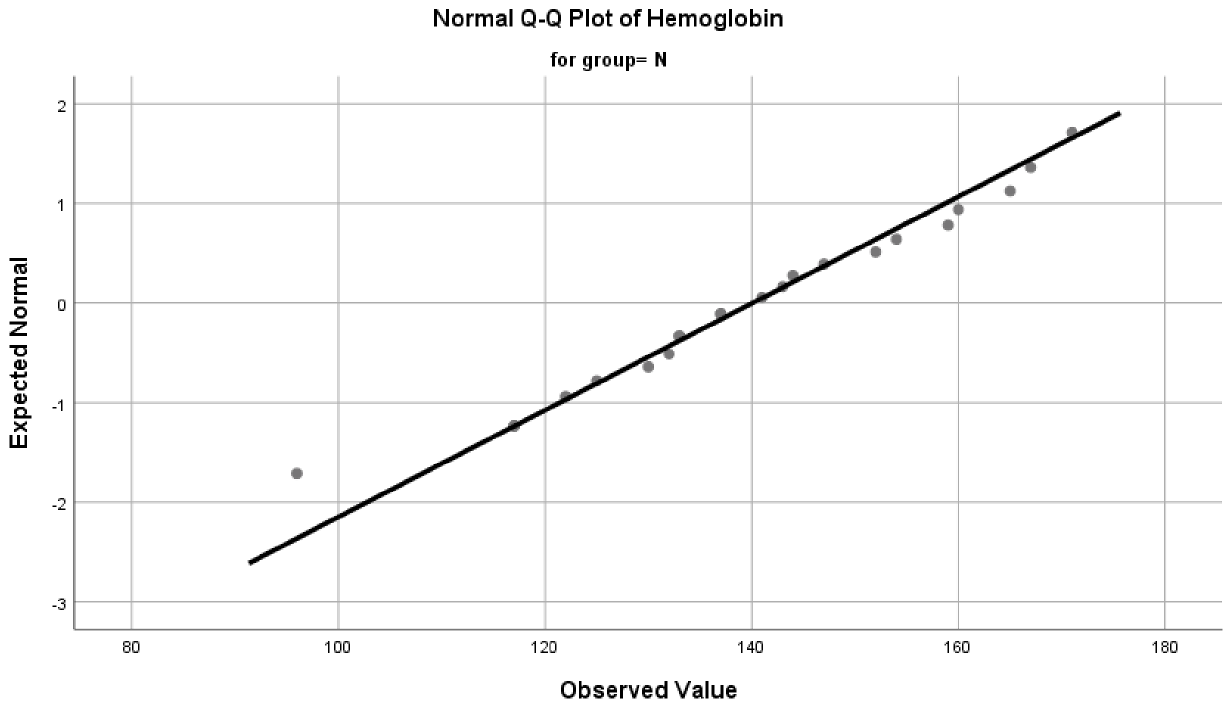
Normal Q-Q Plot of Hemoglobin
for group= N
Expected Normal
Observed Value
2
1
0
-1
-2
-3
80
100
120
140
160
180

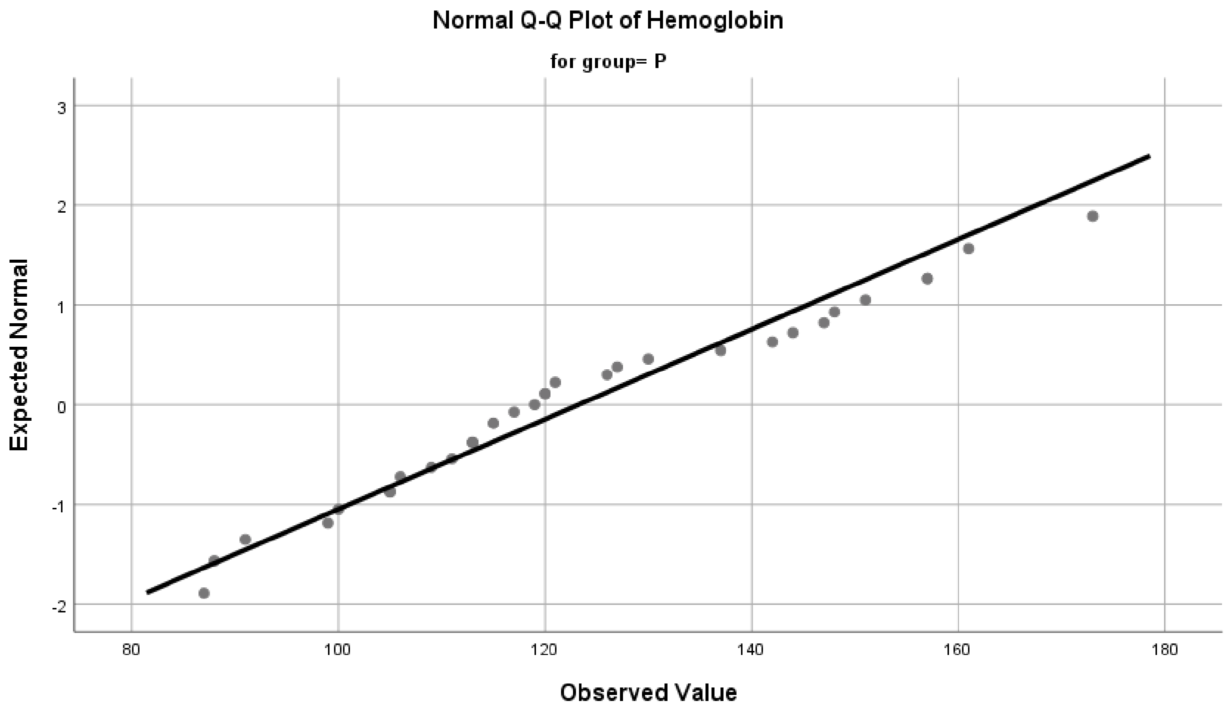
Normal Q-Q Plot of Hemoglobin
for group= P
Expected Normal
Observed Value
3
2
1
0
-1
-2
80
100
120
140
160
180

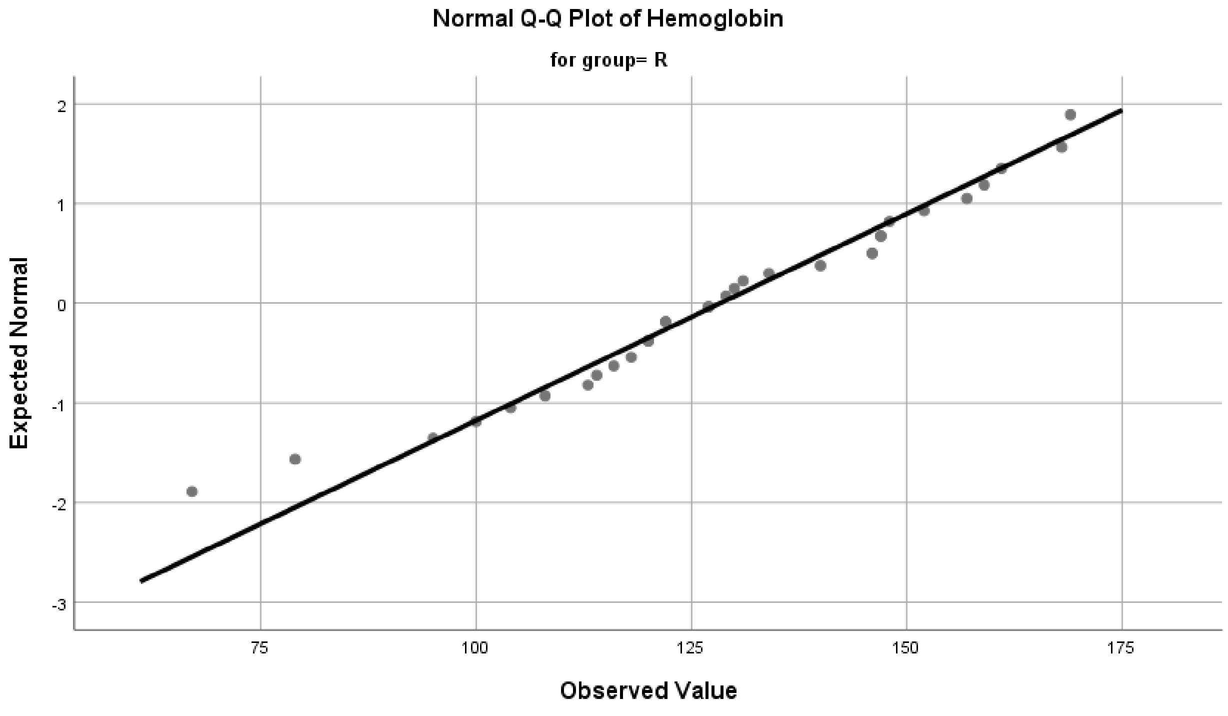
Normal Q-Q Plot of Hemoglobin
for group= R
Expected Normal
Observed Value
2
1
0
-1
-2
-3
75
100
125
150
175

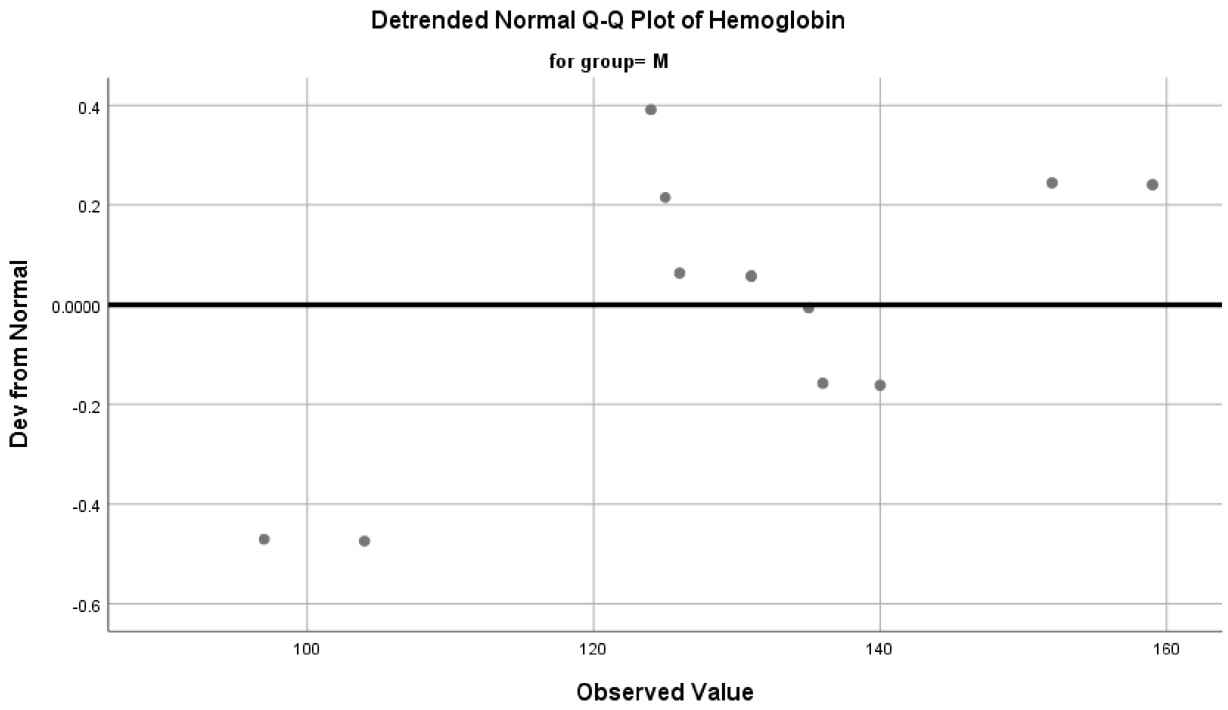

Detrended Normal Q-Q Plot of Hemoglobin
for group= M
Dev from Normal
0.4
0.2
0.0000
-0.2
-0.4
-0.6
100
120
140
160
Observed Value

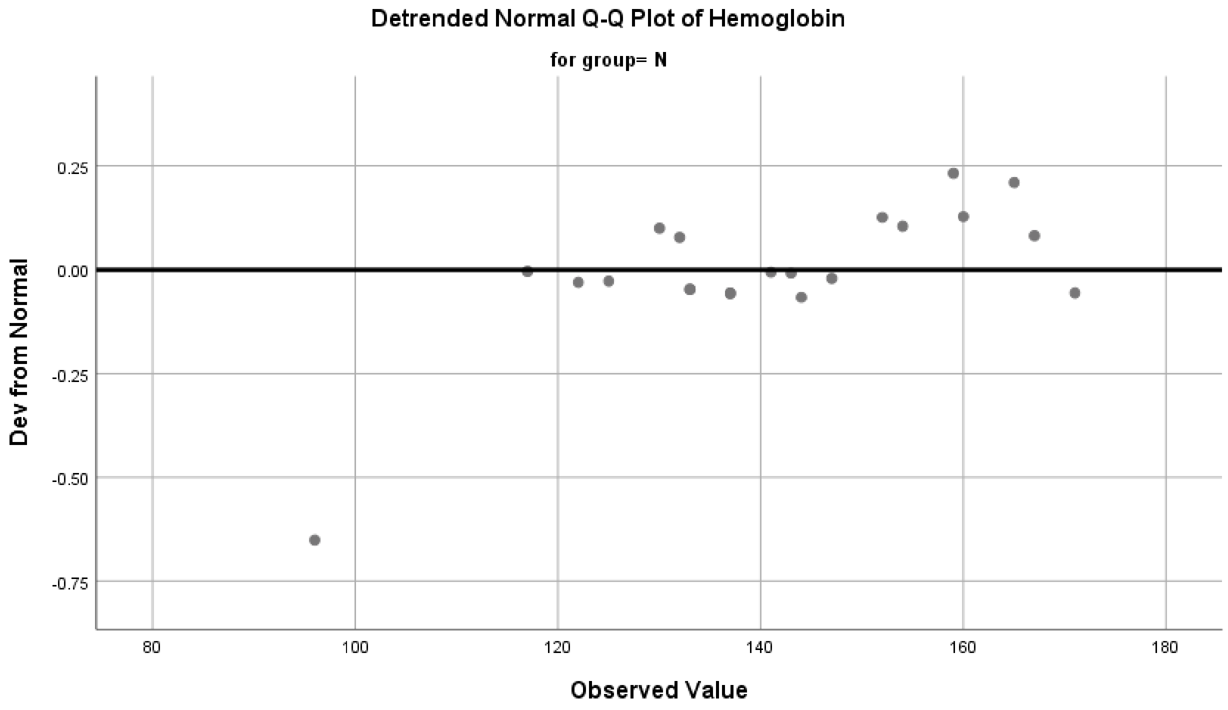

Detrended Normal Q-Q Plot of Hemoglobin
for group= N
Dev from Normal
0.25
0.00
-0.25
-0.50
-0.75
80
100
120
140
160
180
Observed Value

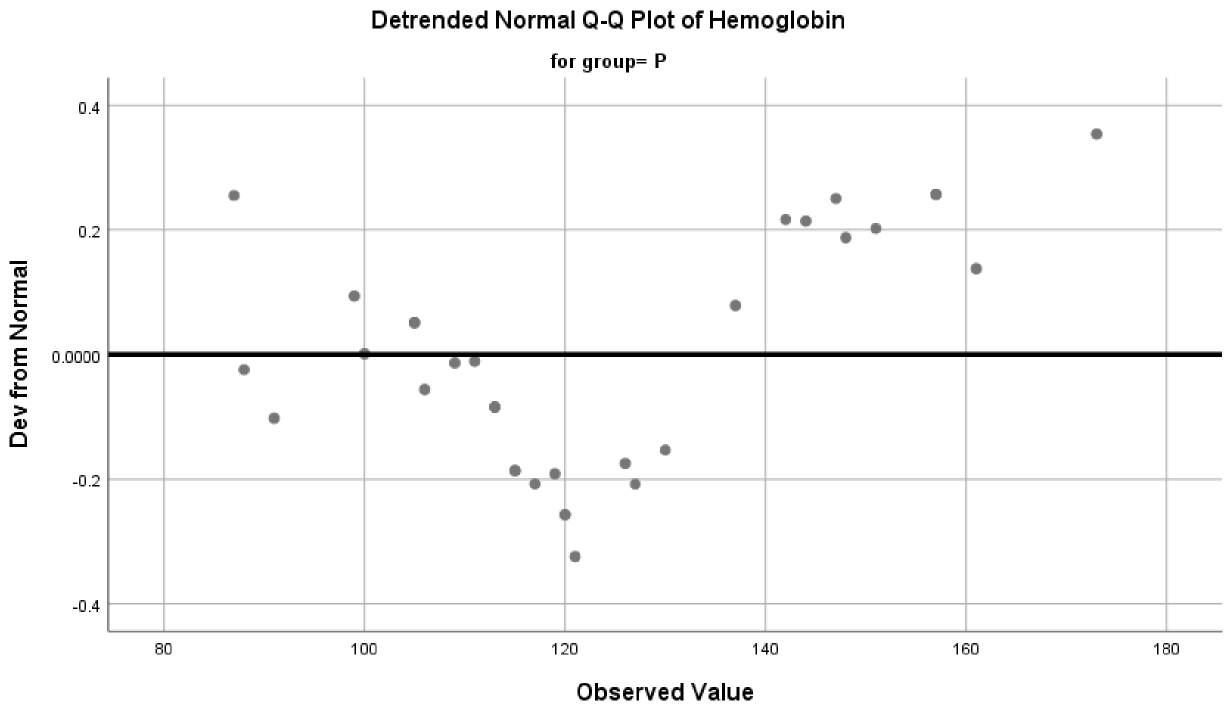
Detrended Normal Q-Q Plot of Hemoglobin
for group= P
Dev from Normal
0.4
0.2
0.0000
-0.2
-0.4
80
100
120
140
160
180
Observed Value

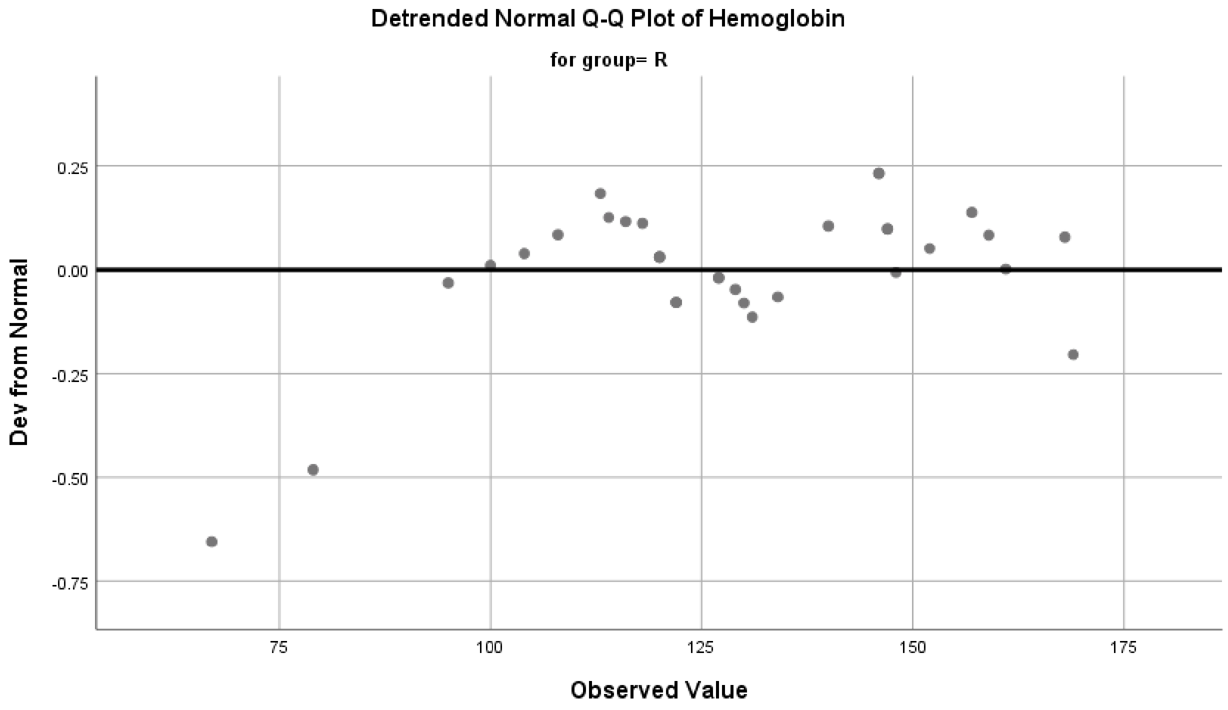
Detrended Normal Q-Q Plot of Hemoglobin
for group= R
Dev from Normal
0.25
0.00
-0.25
-0.50
-0.75
75
100
125
150
175
Observed Value

Normal Q-Q Plot of CRP

for group= M

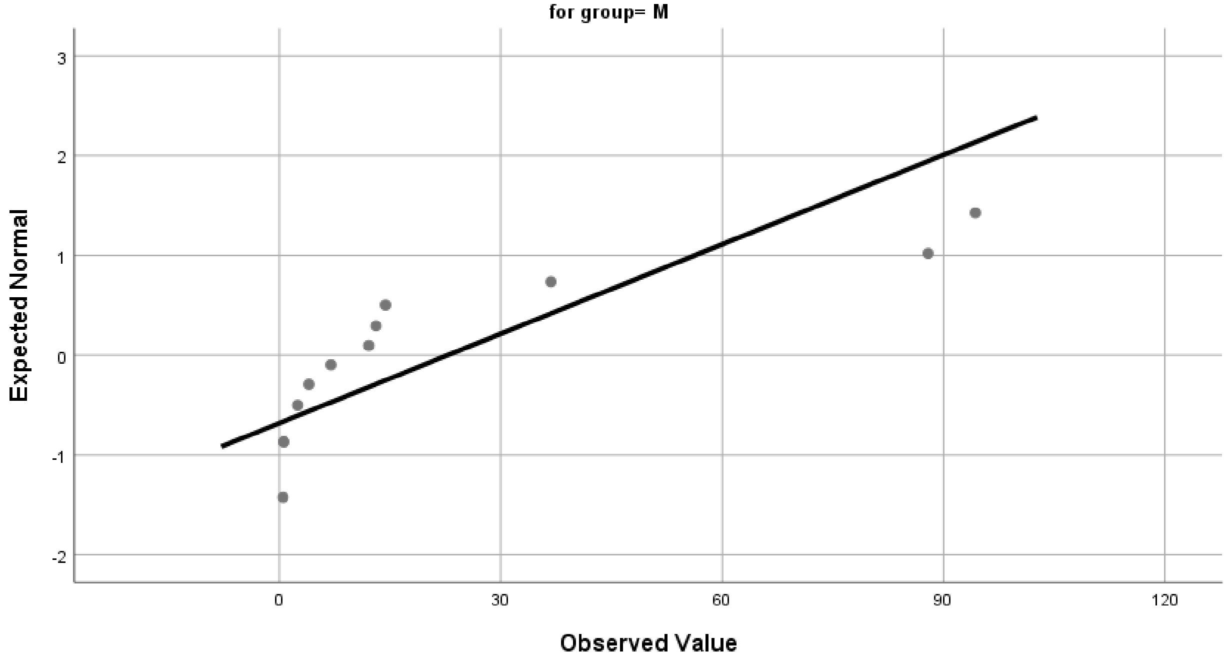

Normal Q-Q Plot of CRP

for group= N

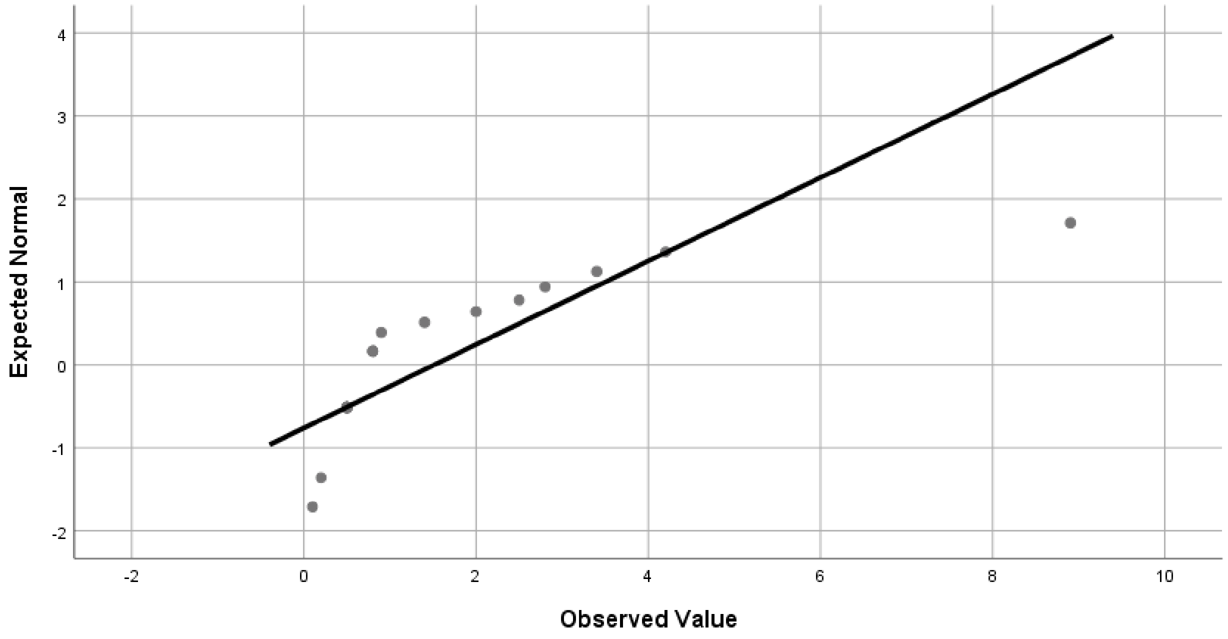

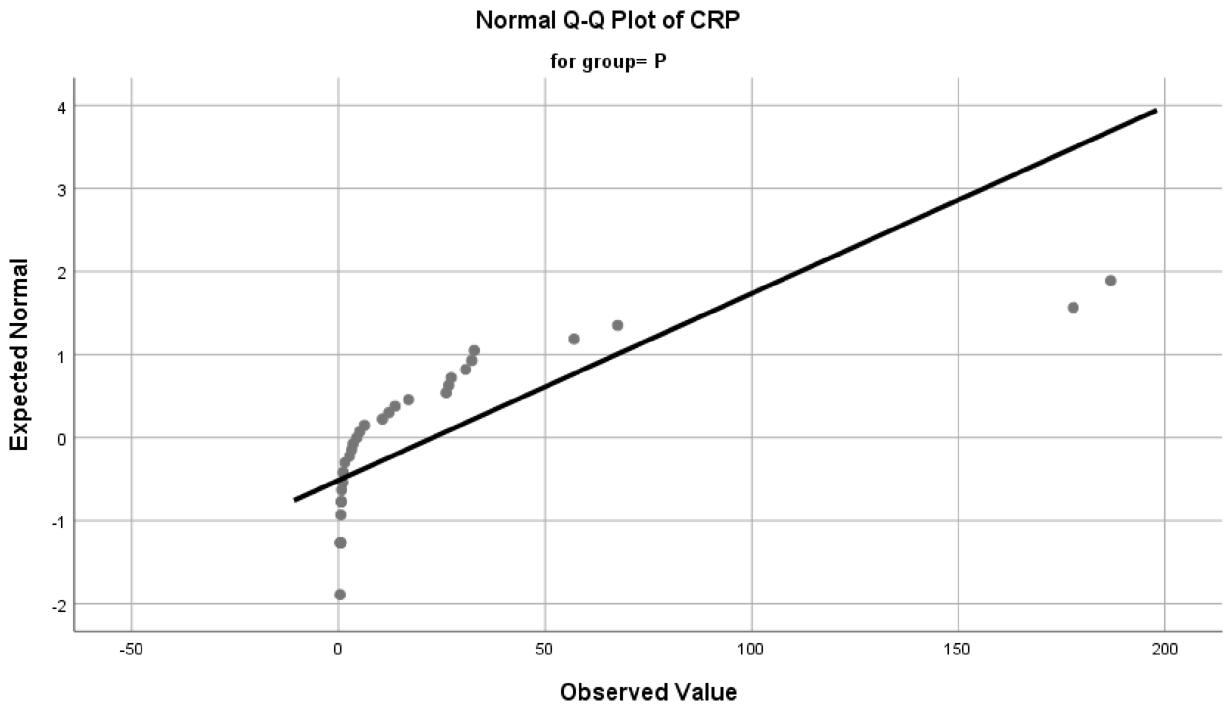
Normal Q-Q Plot of CRP
for group= P
Expected Normal
4
3
2
1
0
-1
-2
-50
0
50
100
150
200
Observed Value

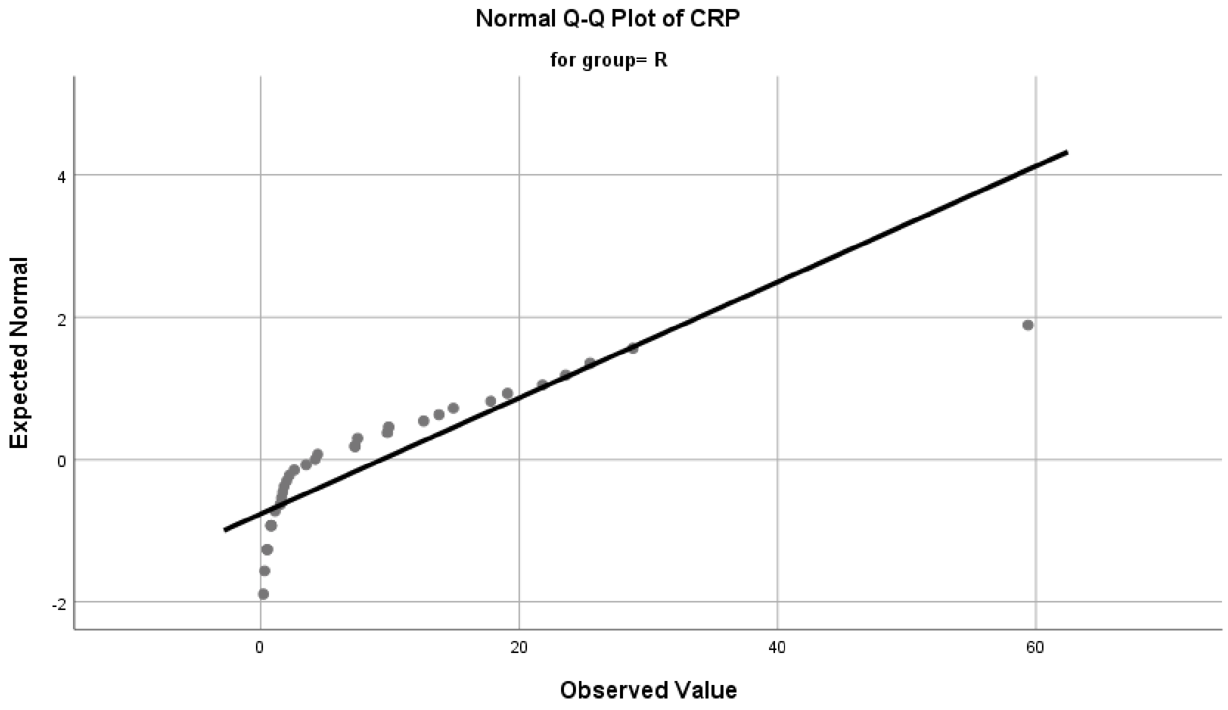
Normal Q-Q Plot of CRP
for group= R
Expected Normal
4
2
0
-2
0
20
40
60
Observed Value

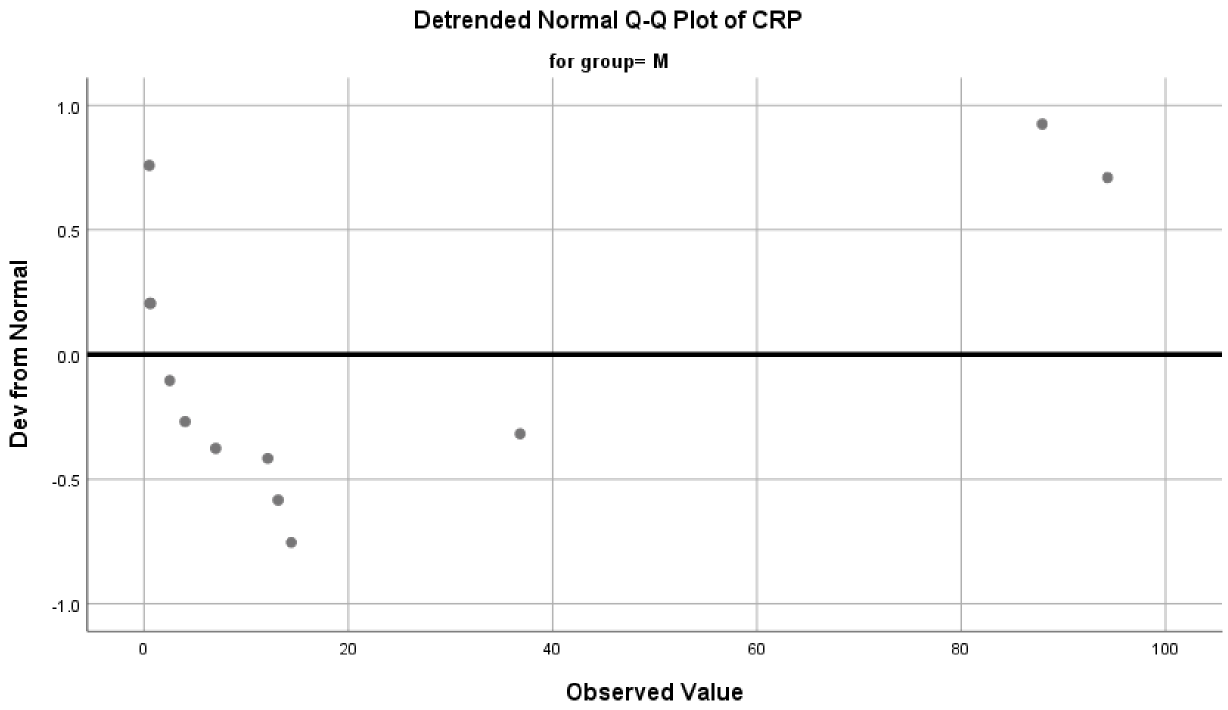
Detrended Normal Q-Q Plot of CRP
for group= M
Dev from Normal
1.0
0.5
0.0
-0.5
-1.0
0
20
40
60
80
100
Observed Value

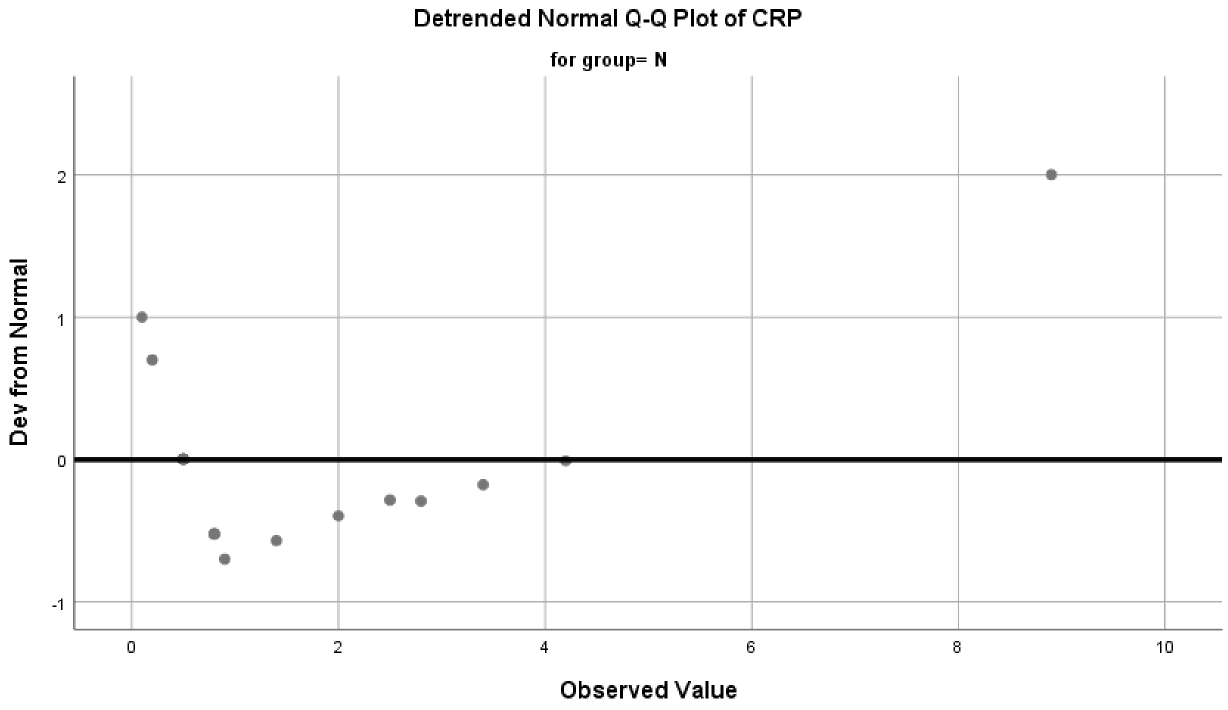
Detrended Normal Q-Q Plot of CRP
for group= N
Dev from Normal
2
1
0
-1
0
2
4
6
8
10
Observed Value

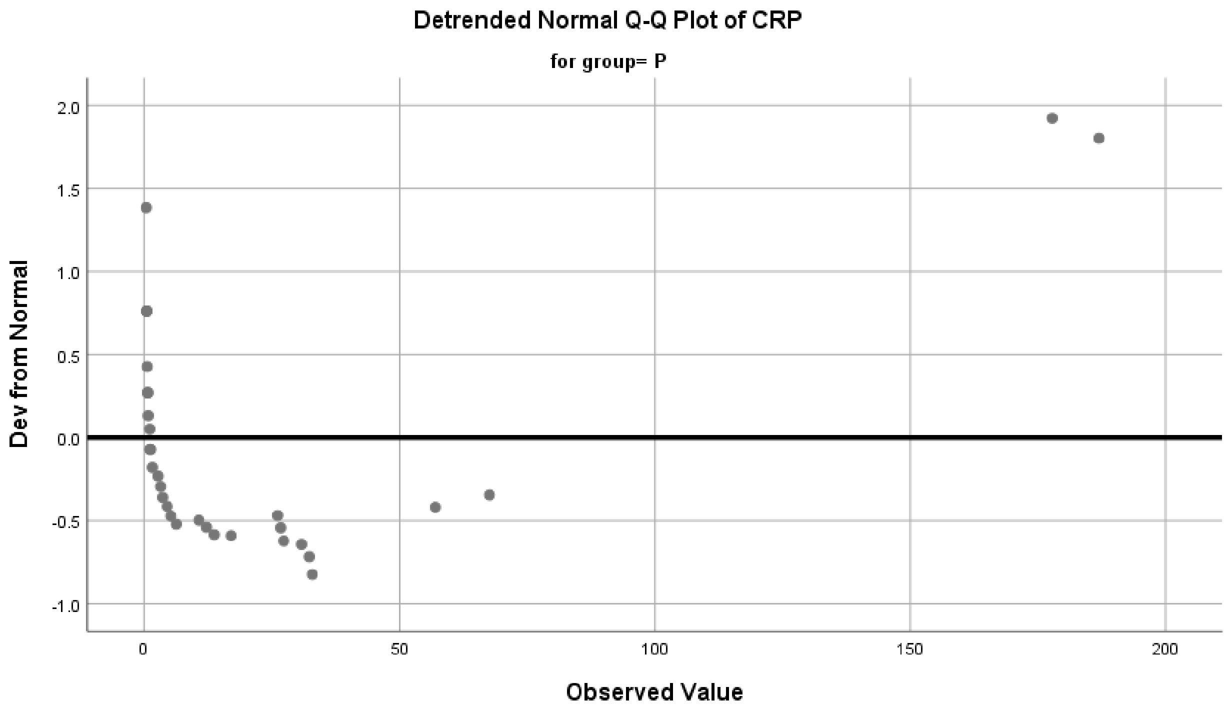
Detrended Normal Q-Q Plot of CRP
for group= P
Dev from Normal
Observed Value
2.0
1.5
1.0
0.5
0.0
-0.5
-1.0
0
50
100
150
200

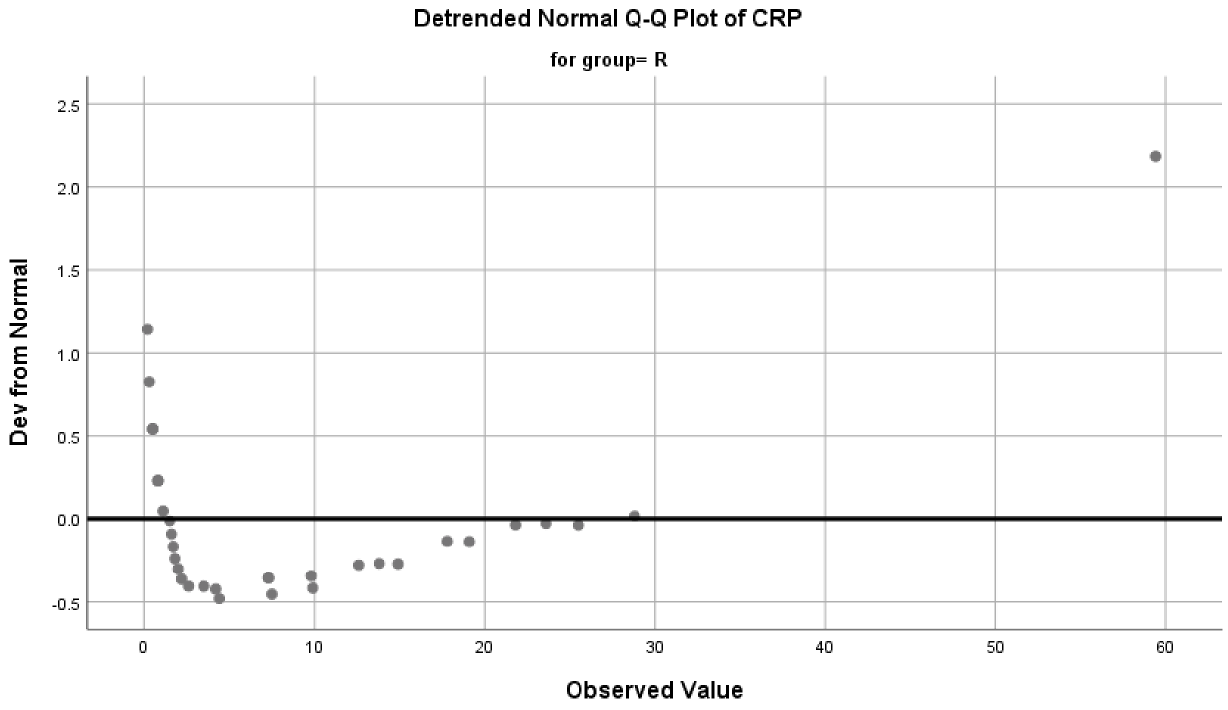
Detrended Normal Q-Q Plot of CRP
for group= R
Dev from Normal
Observed Value
2.5
2.0
1.5
1.0
0.5
0.0
-0.5
0
10
20
30
40
50
60

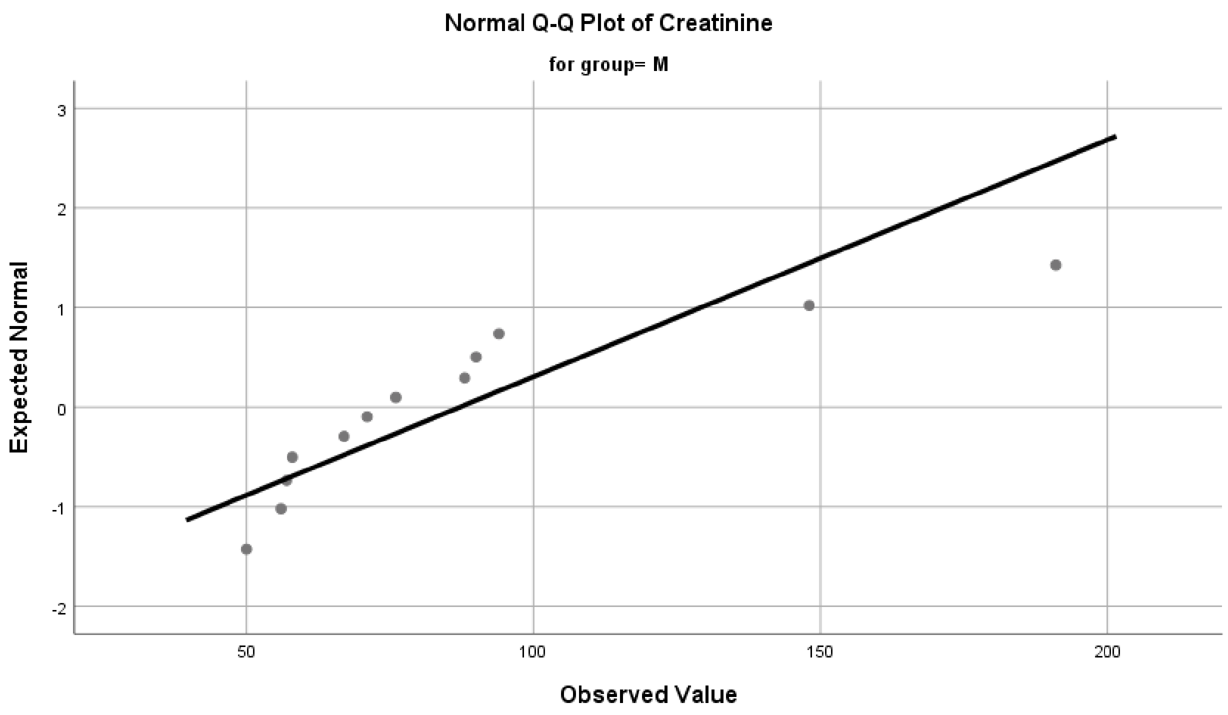
Normal Q-Q Plot of Creatinine
for group= M
Expected Normal
3
2
1
0
-1
-2
50
100
150
200
Observed Value

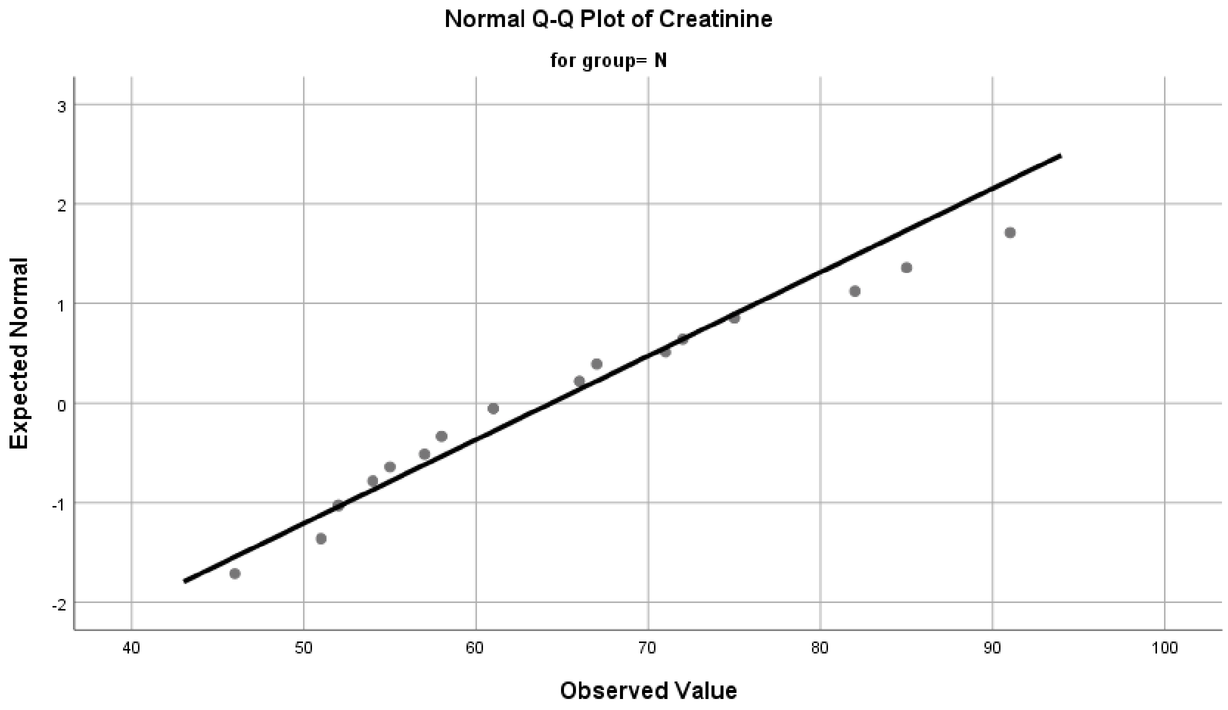
Normal Q-Q Plot of Creatinine
for group= N
Expected Normal
Observed Value
3
2
1
0
-1
-2
40
50
60
70
80
90
100

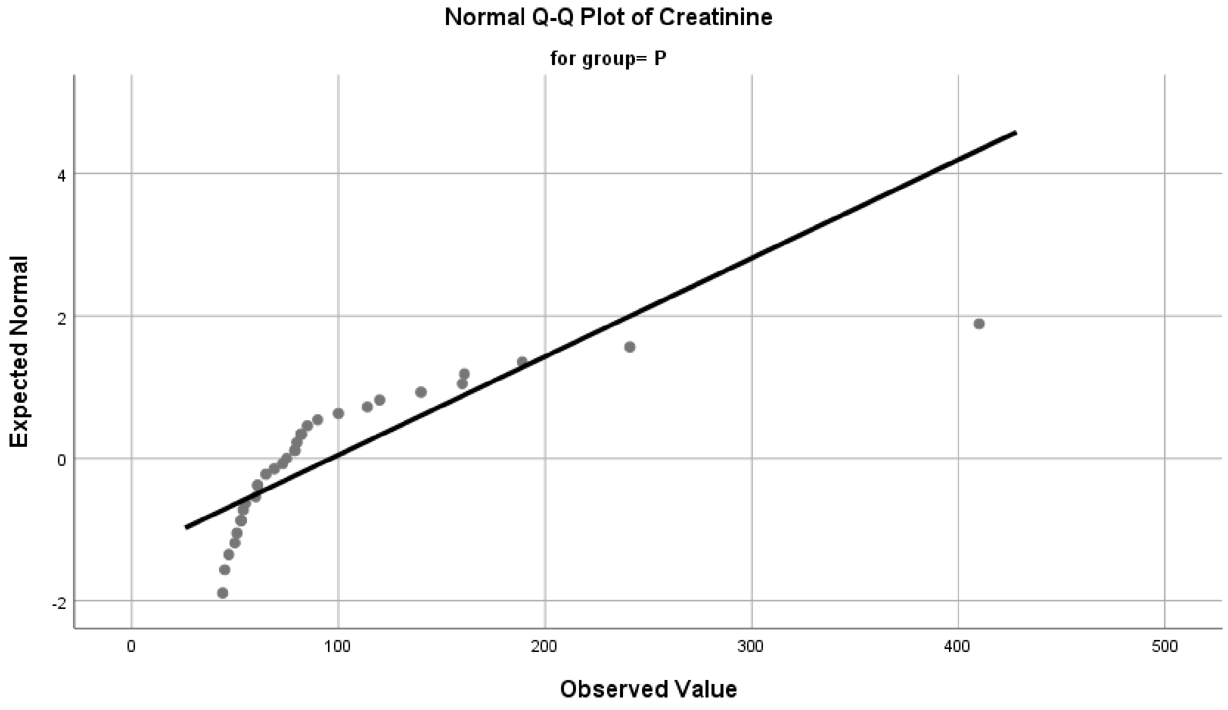
Normal Q-Q Plot of Creatinine
for group= P
Expected Normal
Observed Value
4
2
0
-2
0
100
200
300
400
500

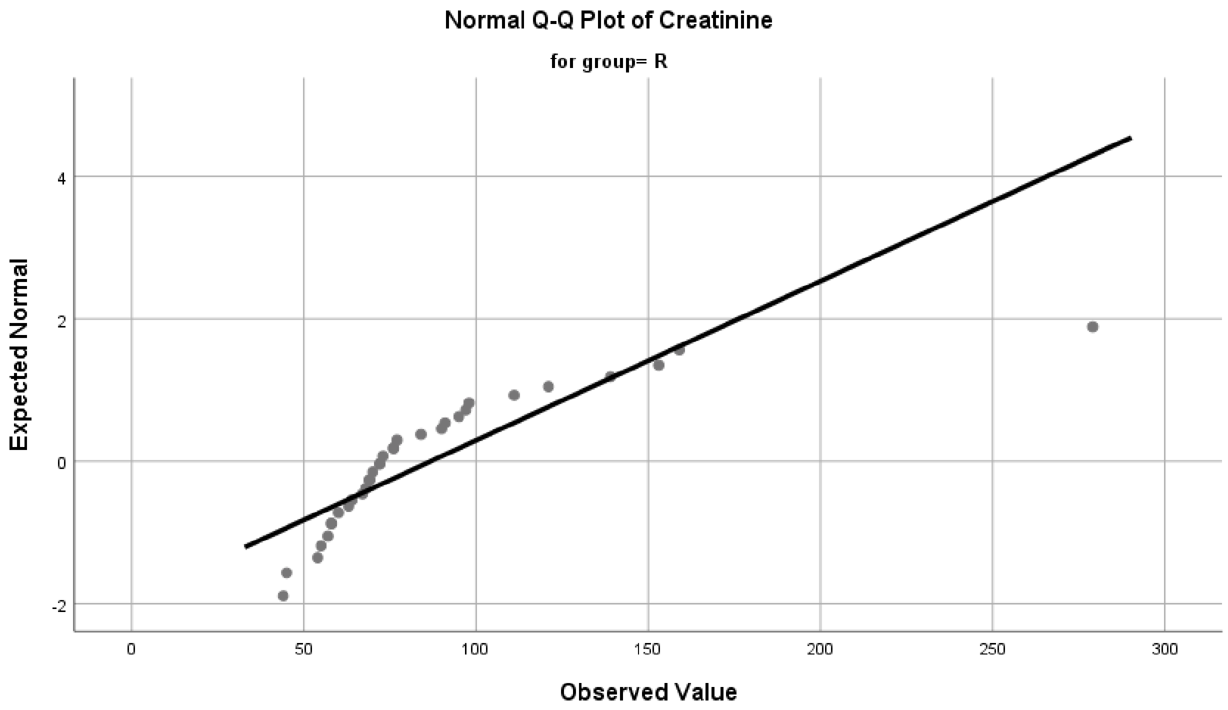

Normal Q-Q Plot of Creatinine
for group= R
Expected Normal
Observed Value
4
2
0
-2
0
50
100
150
200
250
300

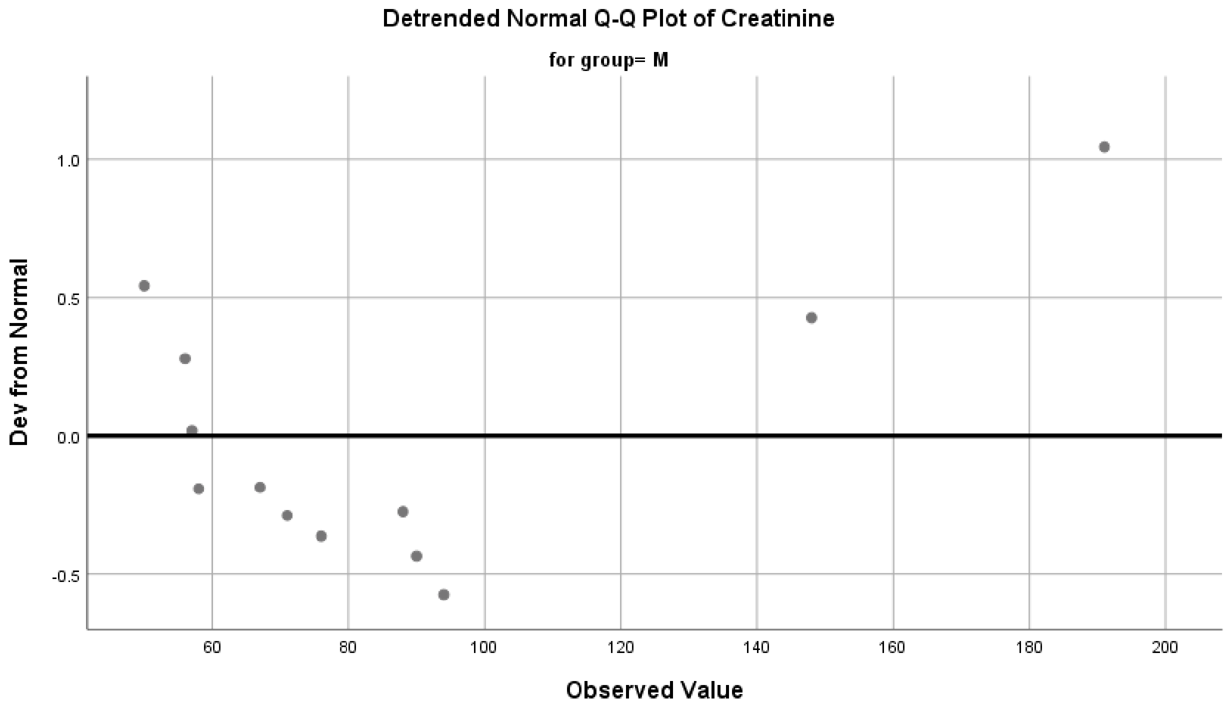

Detrended Normal Q-Q Plot of Creatinine
for group= M
Dev from Normal
Observed Value
1.0
0.5
0.0
-0.5
60
80
100
120
140
160
180
200

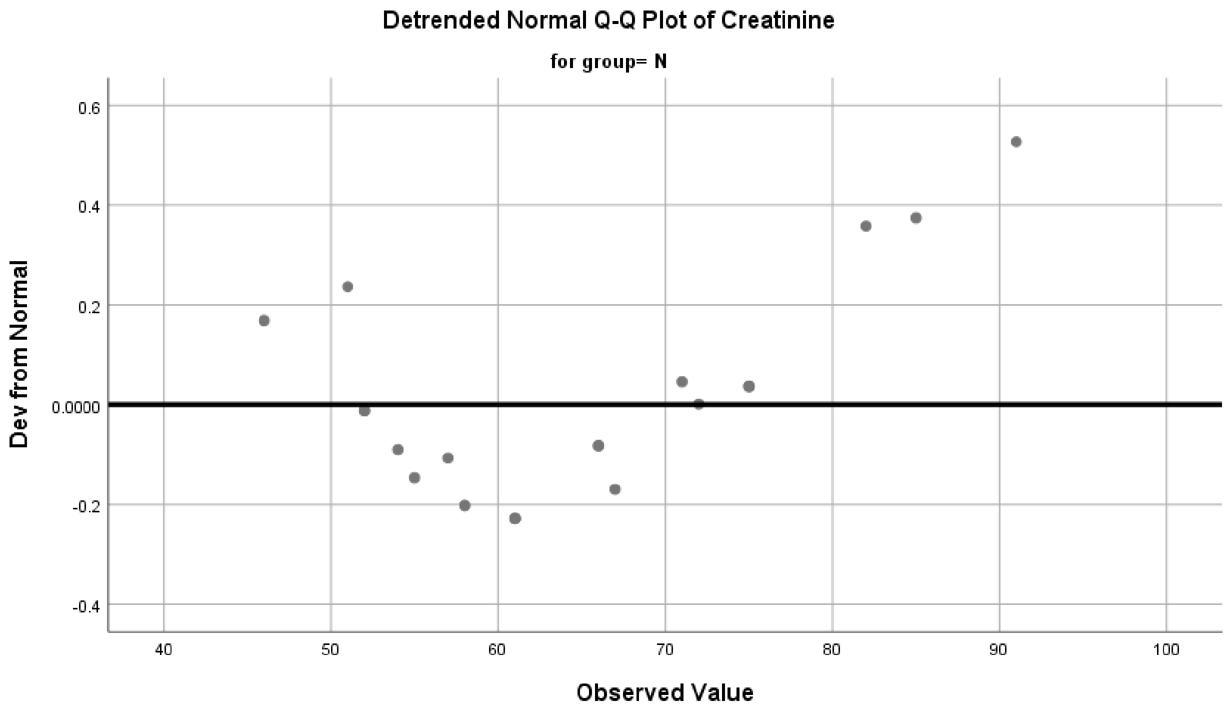
Detrended Normal Q-Q Plot of Creatinine
for group= N
Dev from Normal
Observed Value
0.6
0.4
0.2
0.0000
-0.2
-0.4
40
50
60
70
80
90
100

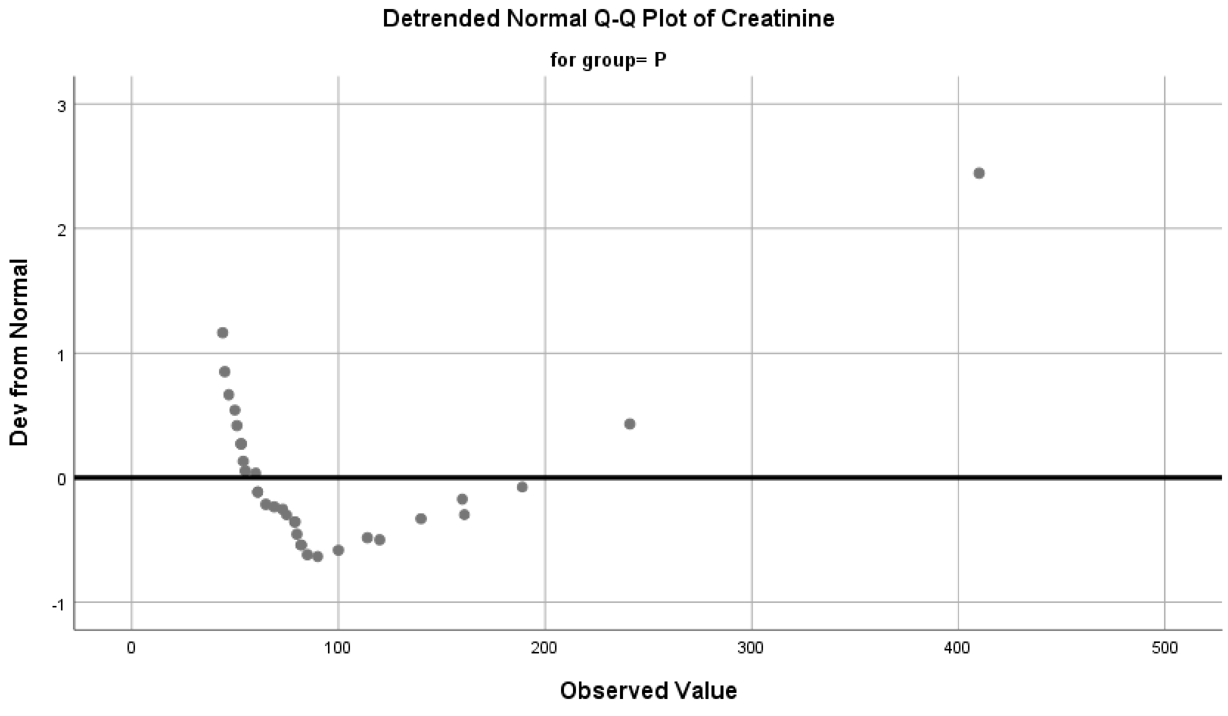
Detrended Normal Q-Q Plot of Creatinine
for group= P
Dev from Normal
Observed Value
3
2
1
0
-1
0
100
200
300
400
500

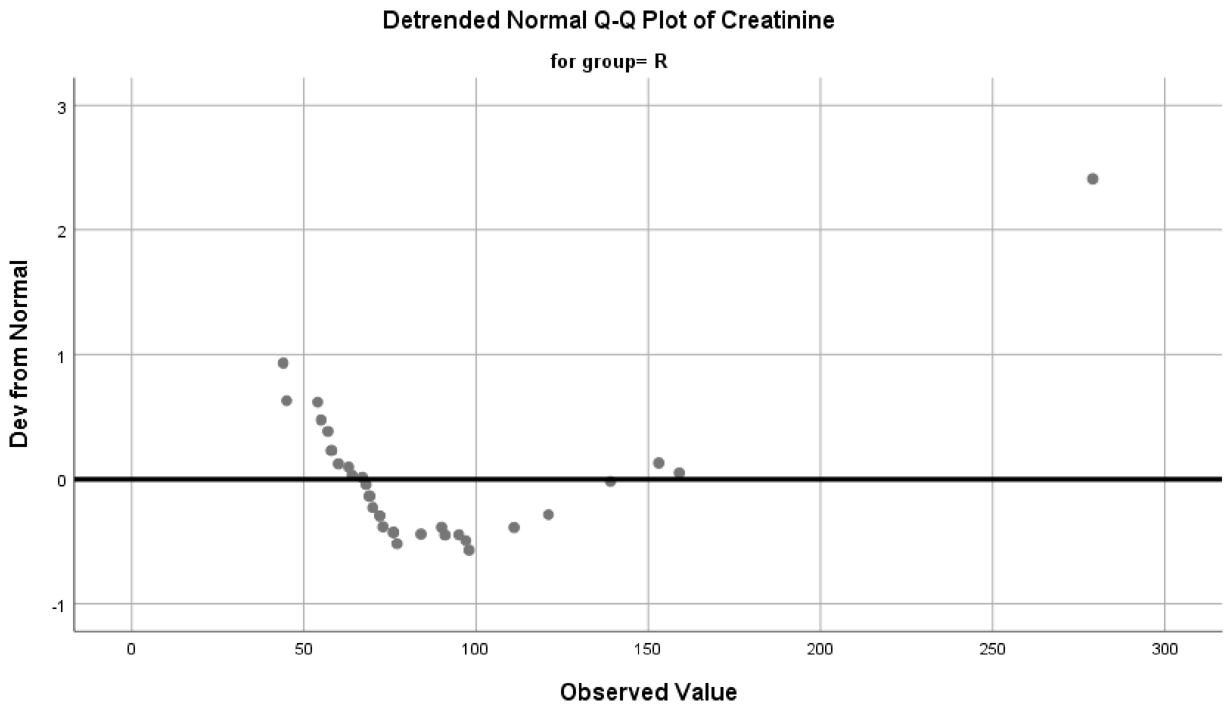

Detrended Normal Q-Q Plot of Creatinine
for group= R
Dev from Normal
3
2
1
0
-1
0
50
100
150
200
250
300
Observed Value

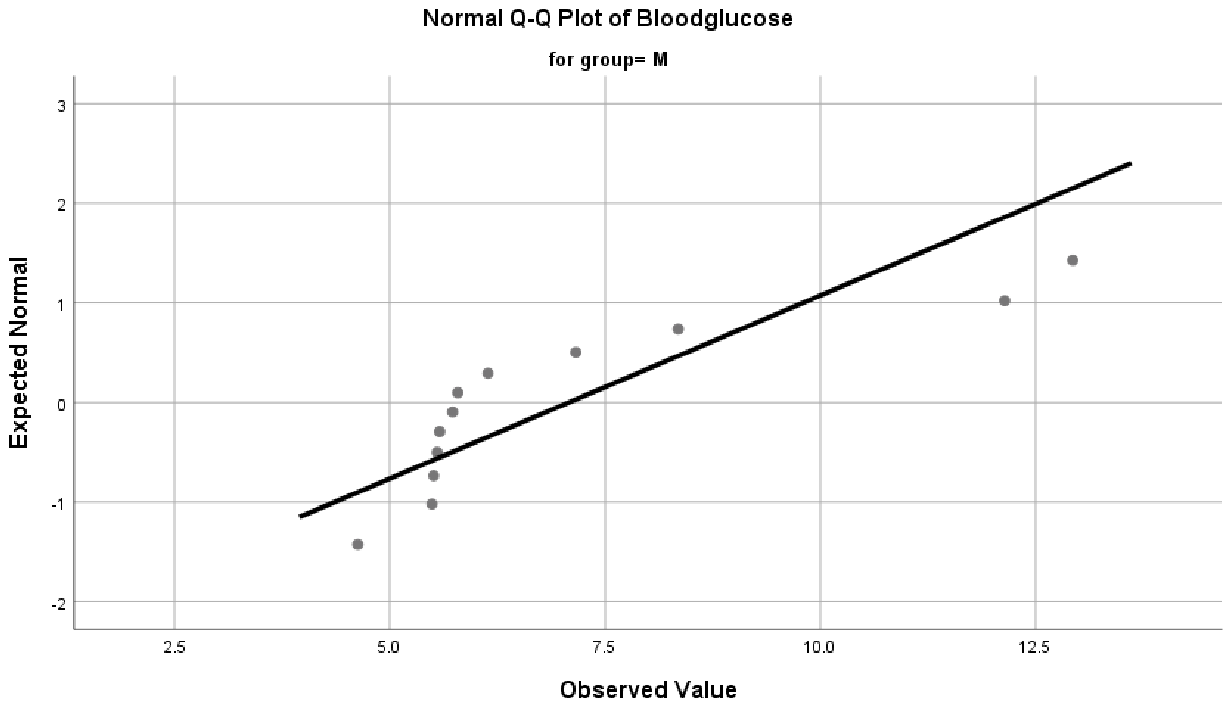

Normal Q-Q Plot of Bloodglucose
for group= M
Expected Normal
3
2
1
0
-1
-2
2.5
5.0
7.5
10.0
12.5
Observed Value

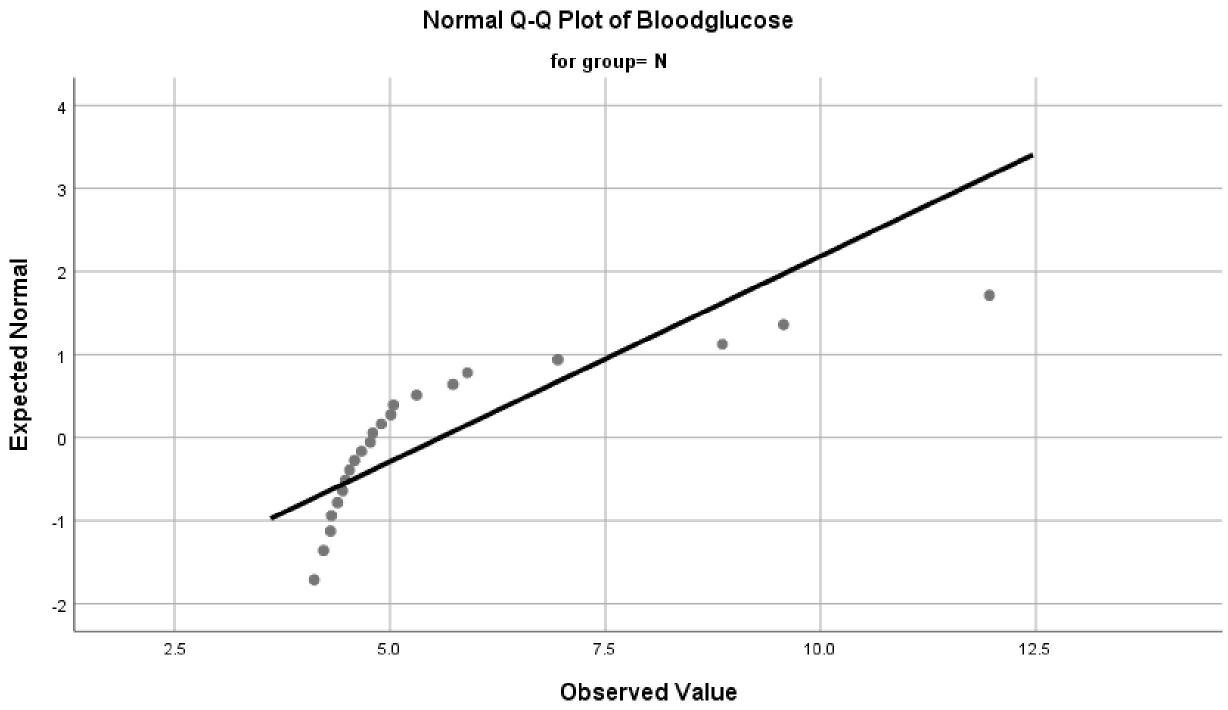
Normal Q-Q Plot of Bloodglucose
for group= N
Expected Normal
Observed Value
4
3
2
1
0
-1
-2
2.5
5.0
7.5
10.0
12.5

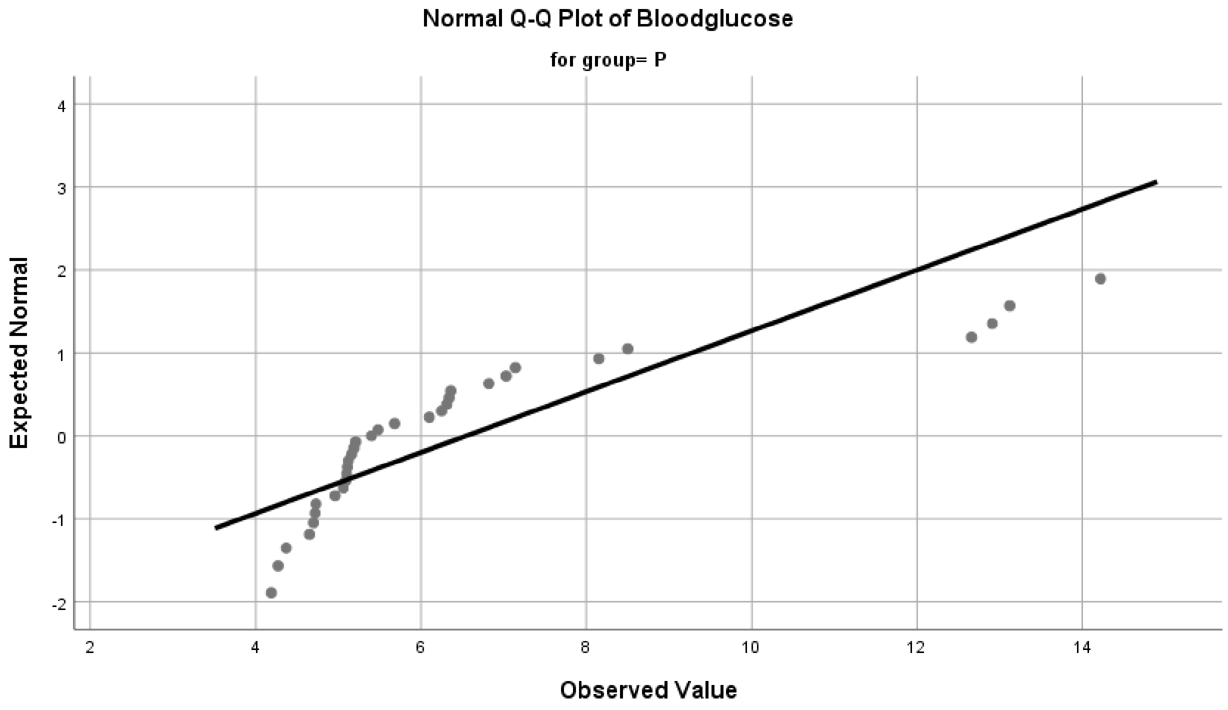
Normal Q-Q Plot of Bloodglucose
for group= P
Expected Normal
Observed Value
4
3
2
1
0
-1
-2
2
4
6
8
10
12
14

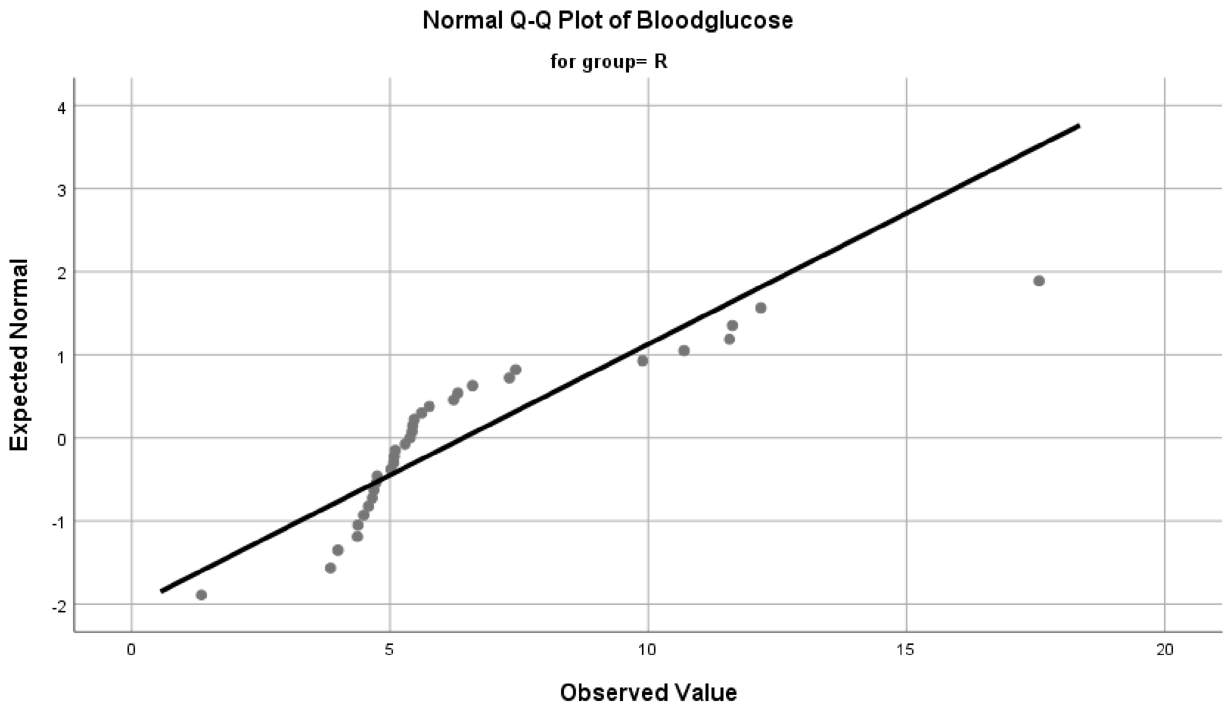
Normal Q-Q Plot of Bloodglucose
for group= R
Expected Normal
Observed Value
4
3
2
1
0
-1
-2
0
5
10
15
20

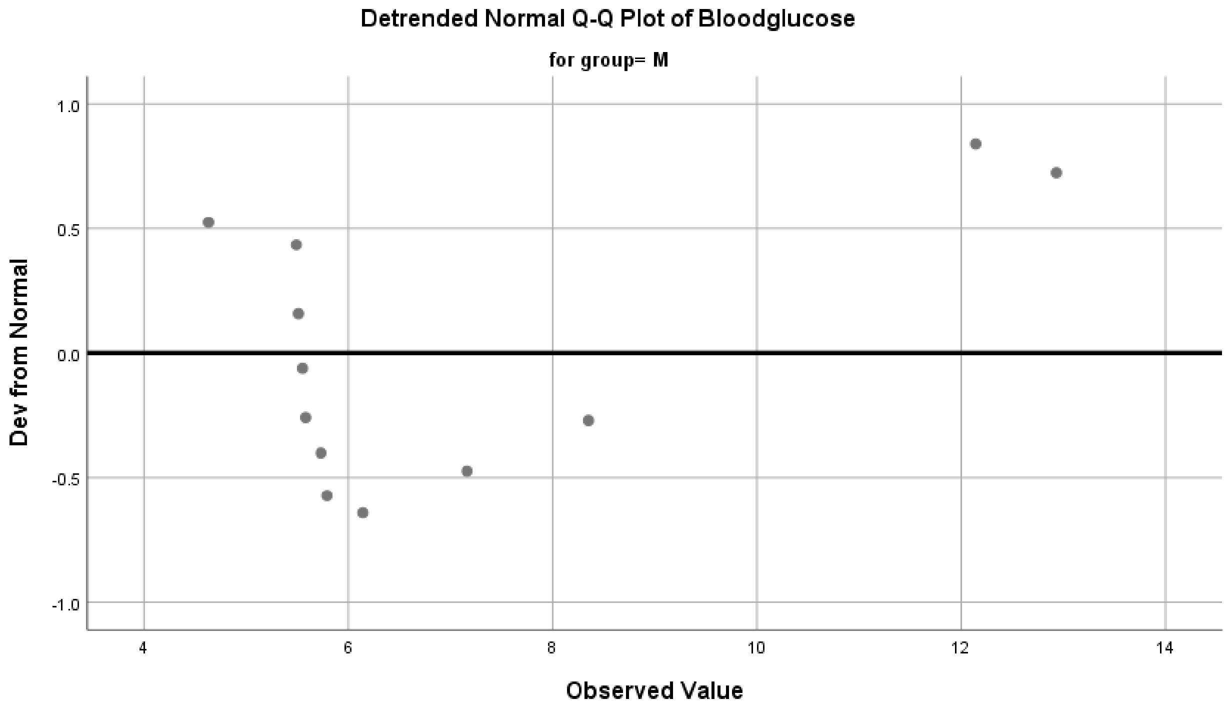
Detrended Normal Q-Q Plot of Bloodglucose
for group= M
Dev from Normal
Observed Value
1.0
0.5
0.0
-0.5
-1.0
4
6
8
10
12
14

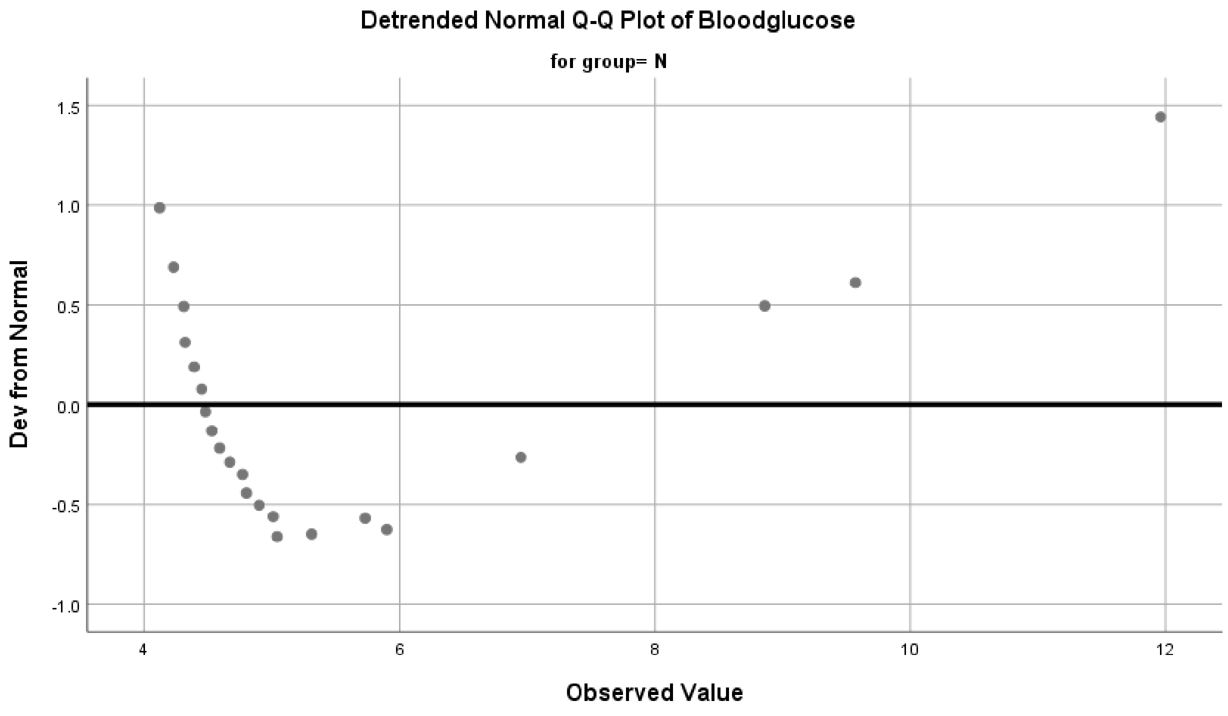
Detrended Normal Q-Q Plot of Bloodglucose
for group= N
Dev from Normal
1.5
1.0
0.5
0.0
-0.5
-1.0
4
6
8
10
12
Observed Value

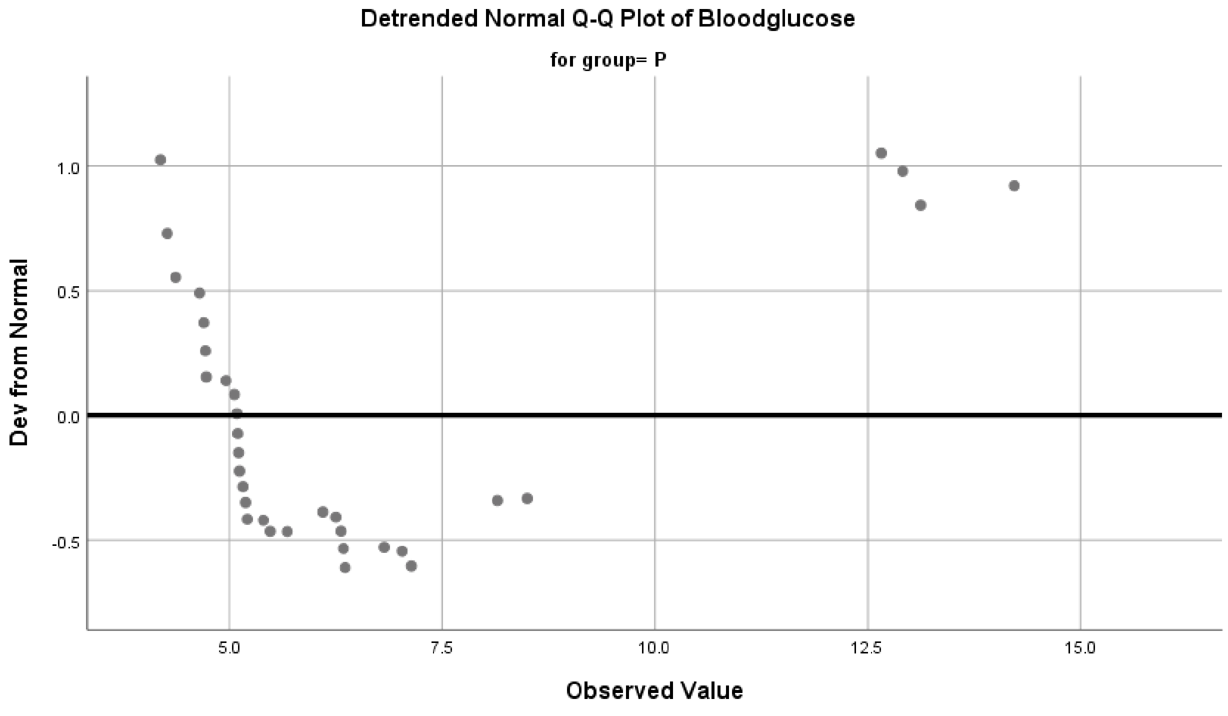
Detrended Normal Q-Q Plot of Bloodglucose
for group= P
Dev from Normal
1.0
0.5
0.0
-0.5
5.0
7.5
10.0
12.5
15.0
Observed Value

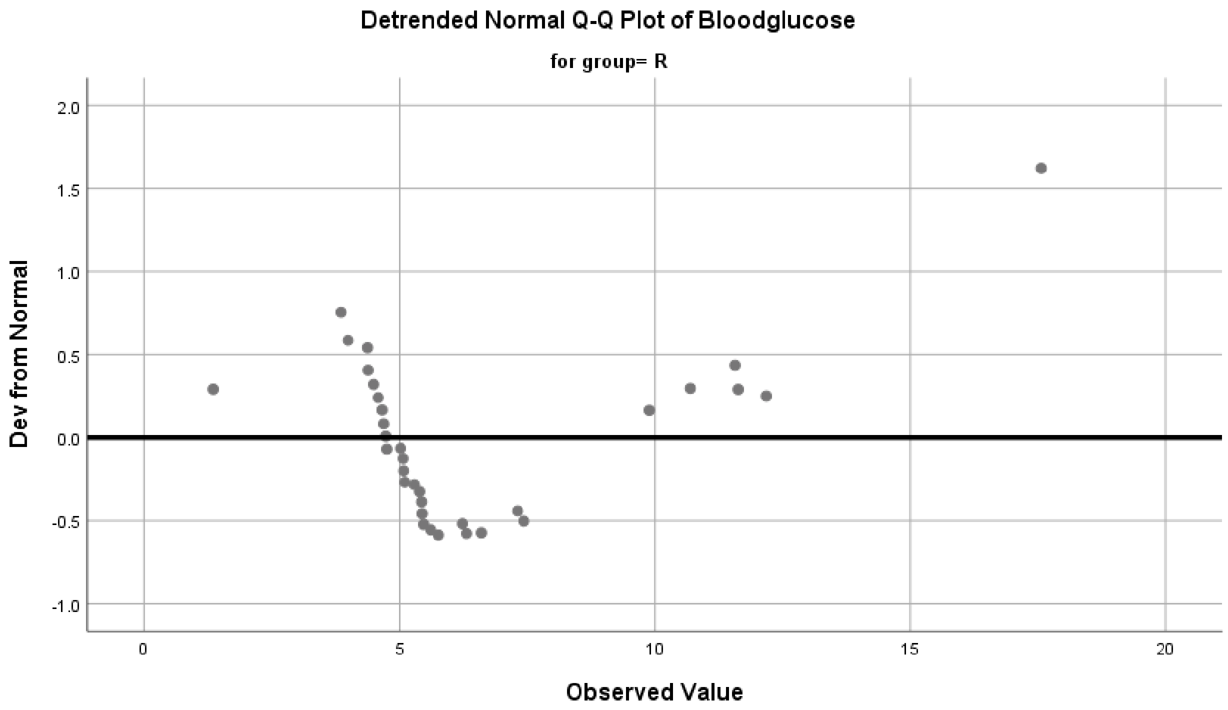
Detrended Normal Q-Q Plot of Bloodglucose
for group= R
Dev from Normal
2.0
1.5
1.0
0.5
0.0
-0.5
-1.0
0
5
10
15
20
Observed Value

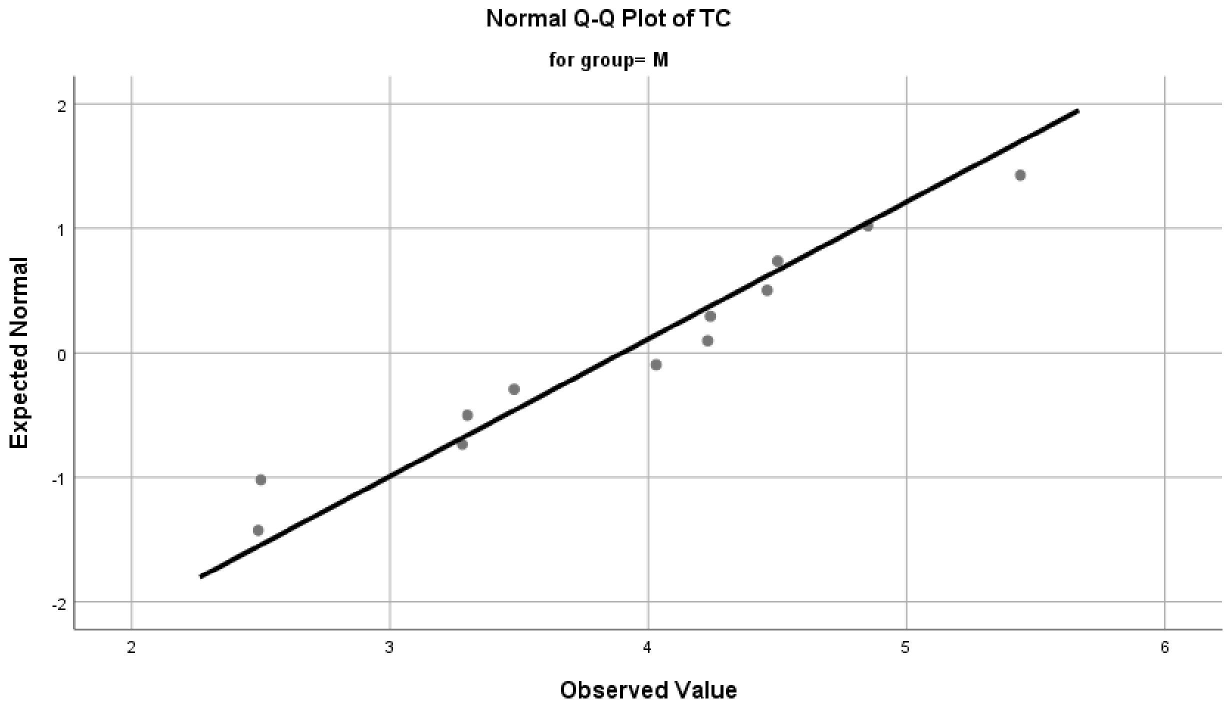
Normal Q-Q Plot of TC
for group= M
Expected Normal
2
1
0
-1
-2
2
3
4
5
6
Observed Value

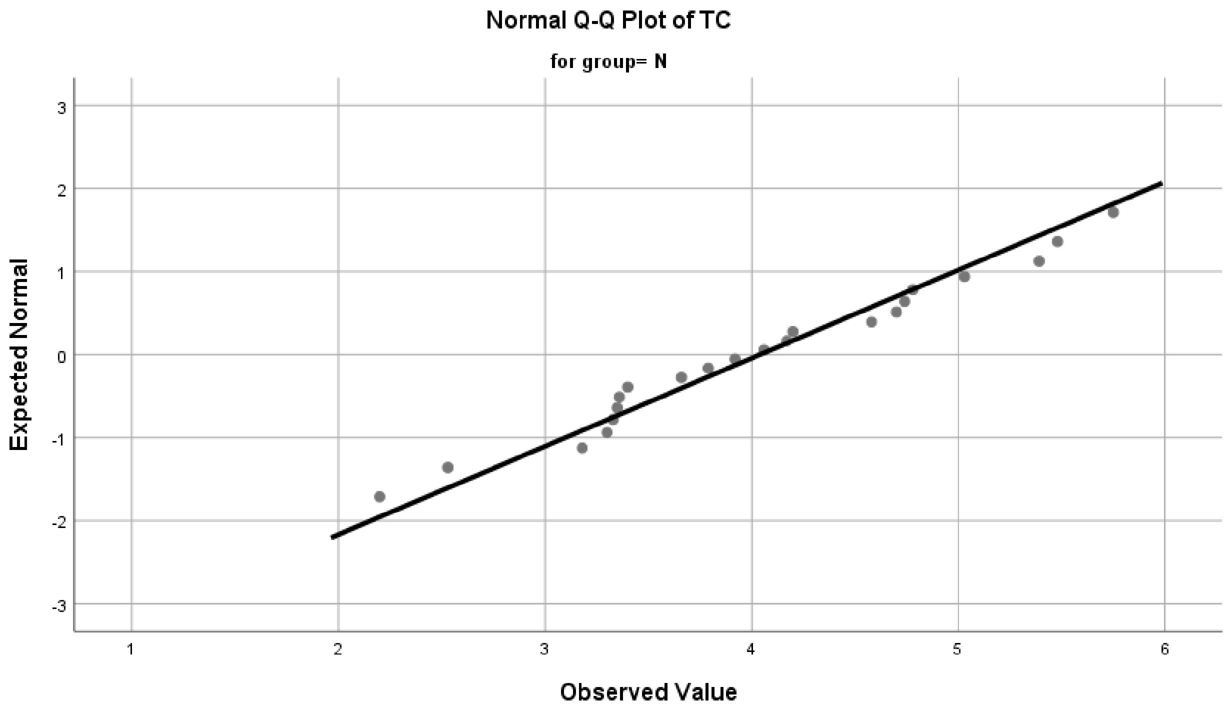
Normal Q-Q Plot of TC
for group= N
Expected Normal
Observed Value

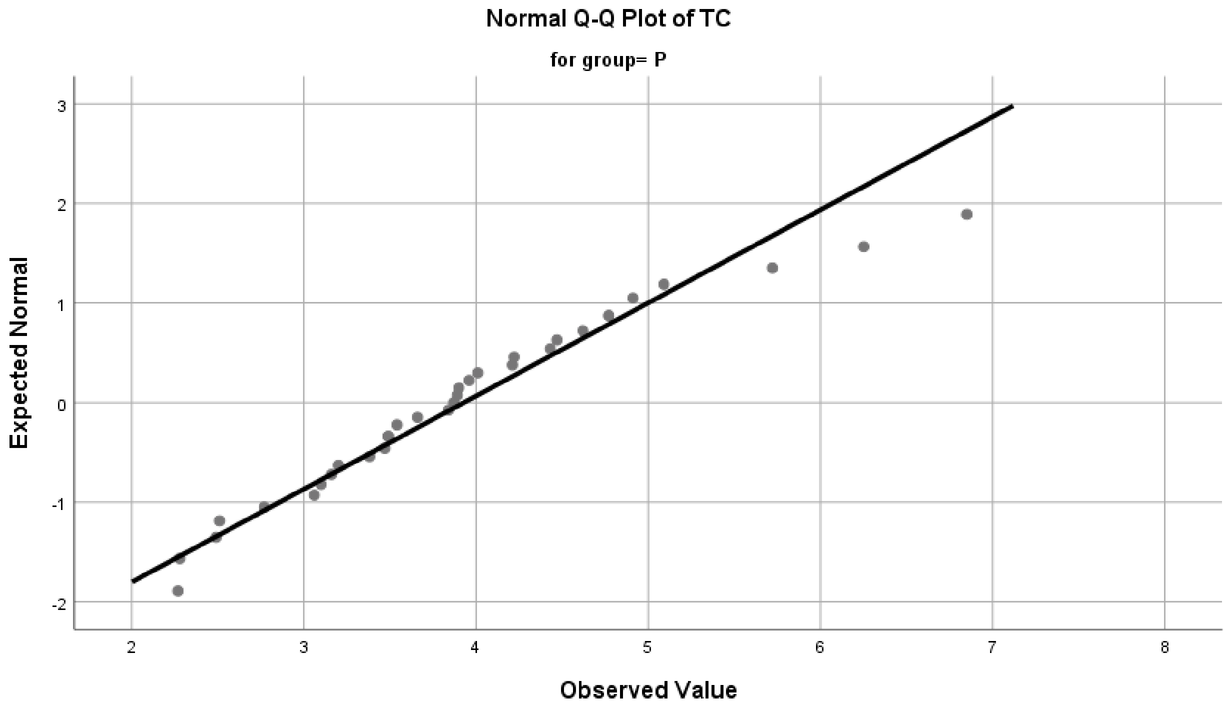
Normal Q-Q Plot of TC
for group= P
Expected Normal
Observed Value

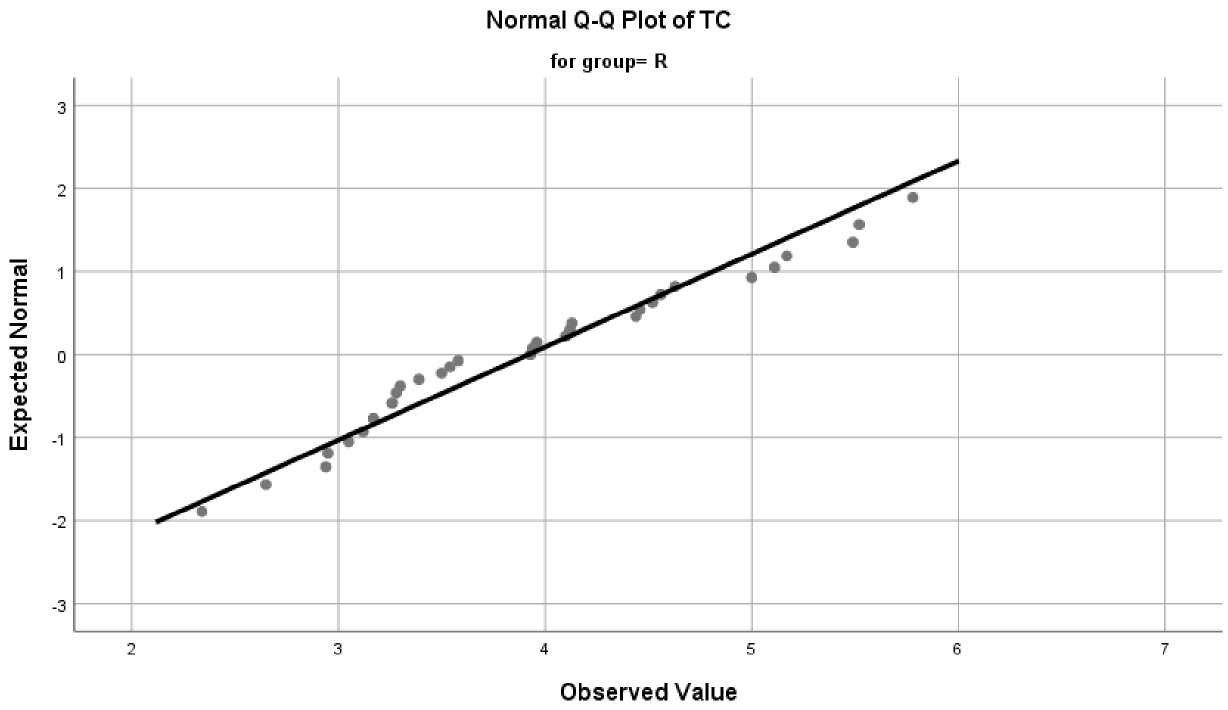

Normal Q-Q Plot of TC
for group= R
Expected Normal
Observed Value
3
2
1
0
-1
-2
-3
2
3
4
5
6
7

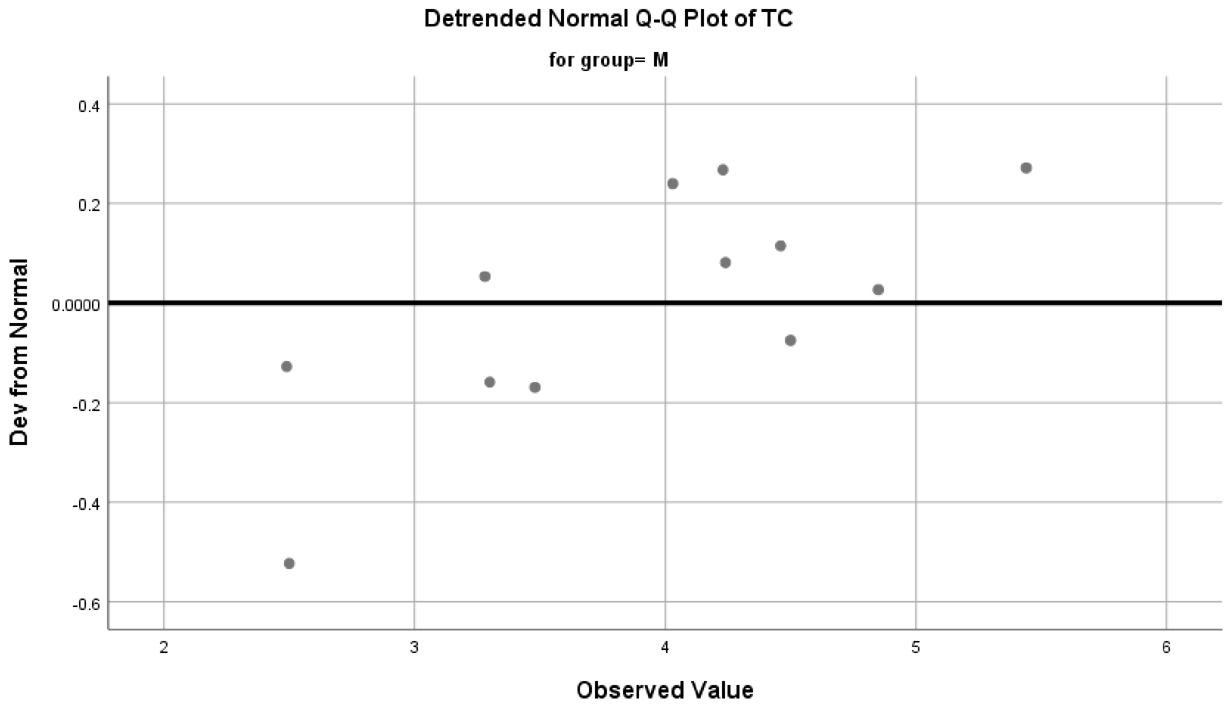

Detrended Normal Q-Q Plot of TC
for group= M
Dev from Normal
Observed Value
0.4
0.2
0.0000
-0.2
-0.4
-0.6
2
3
4
5
6

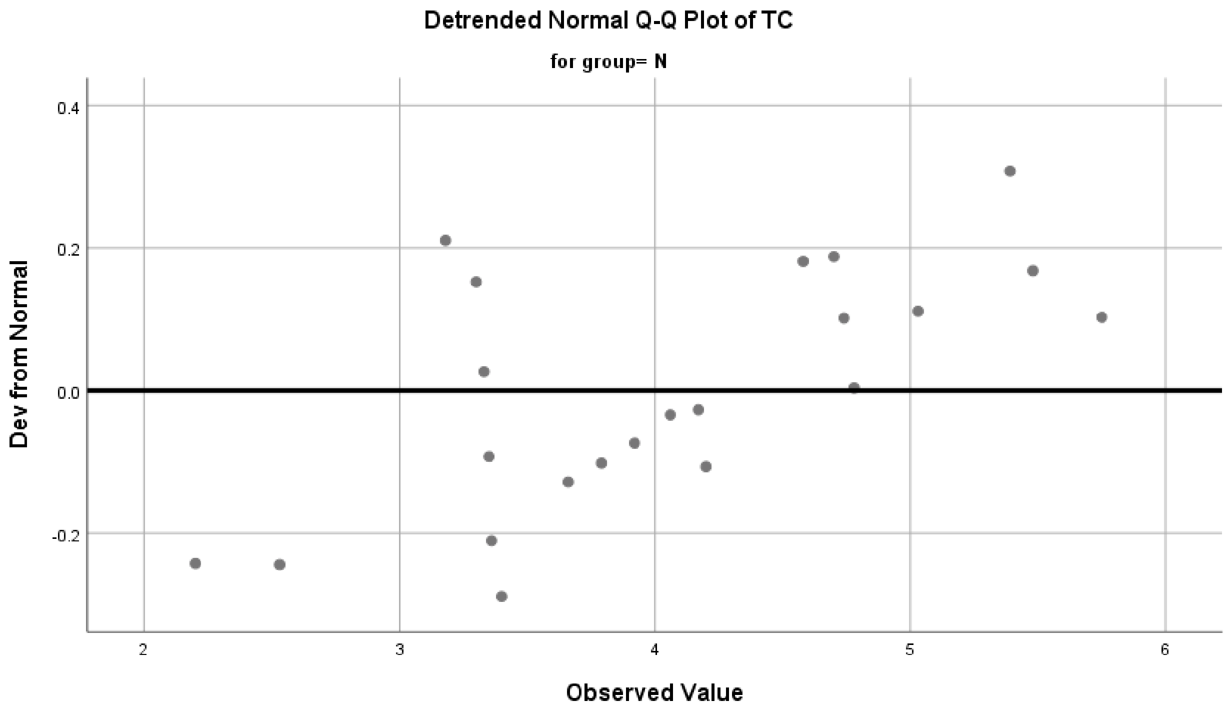

Detrended Normal Q-Q Plot of TC
for group= N
Dev from Normal
0.4
0.2
0.0
-0.2
2
3
4
5
6
Observed Value

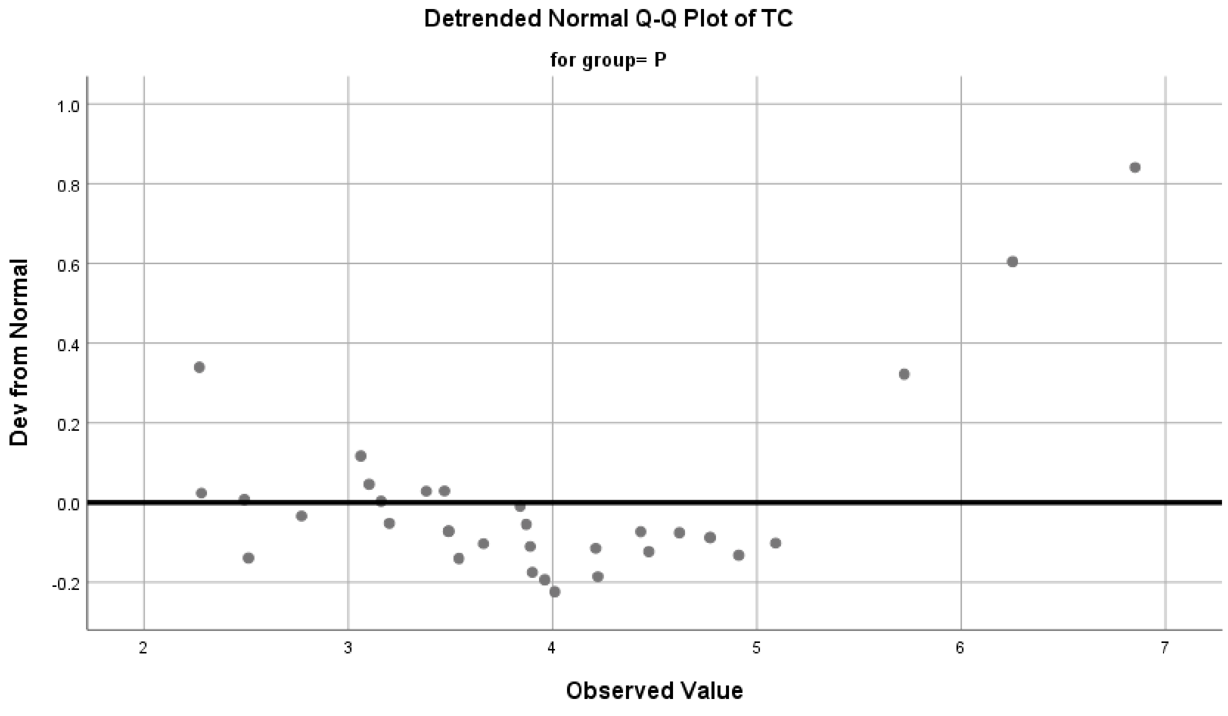

Detrended Normal Q-Q Plot of TC
for group= P
Dev from Normal
1.0
0.8
0.6
0.4
0.2
0.0
-0.2
2
3
4
5
6
7
Observed Value

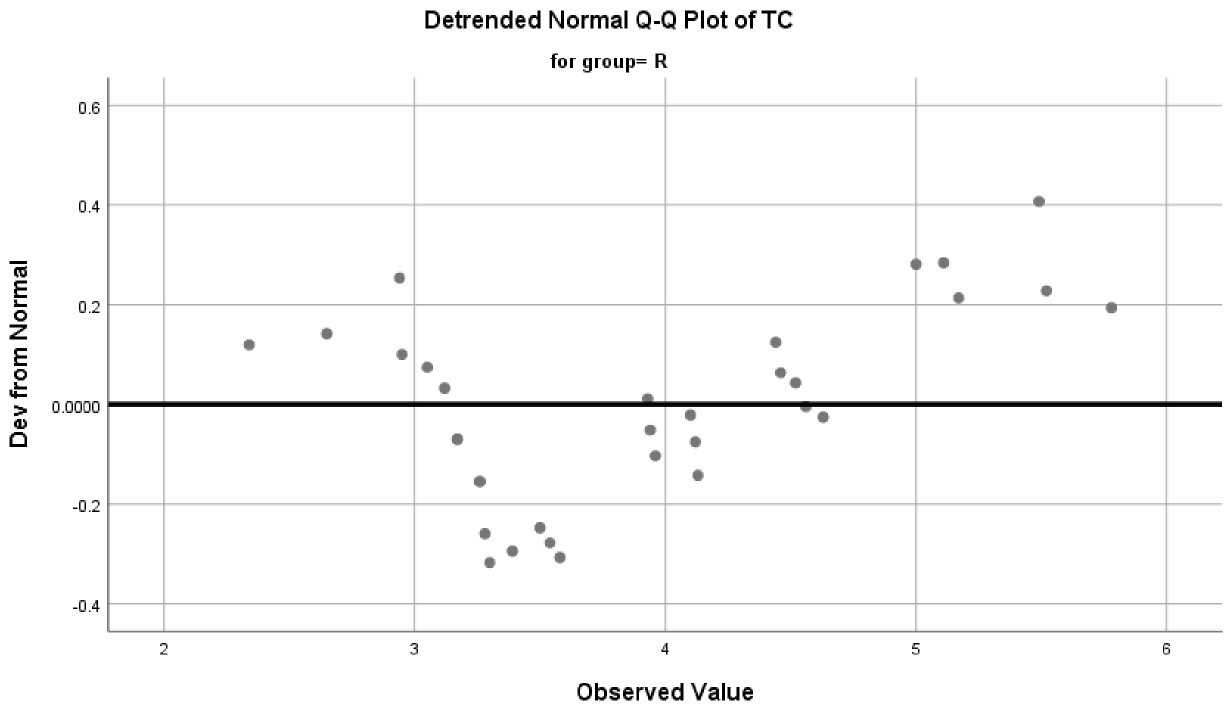
Detrended Normal Q-Q Plot of TC
for group= R
Dev from Normal
0.6
0.4
0.2
0.0000
-0.2
-0.4
2
3
4
5
6
Observed Value

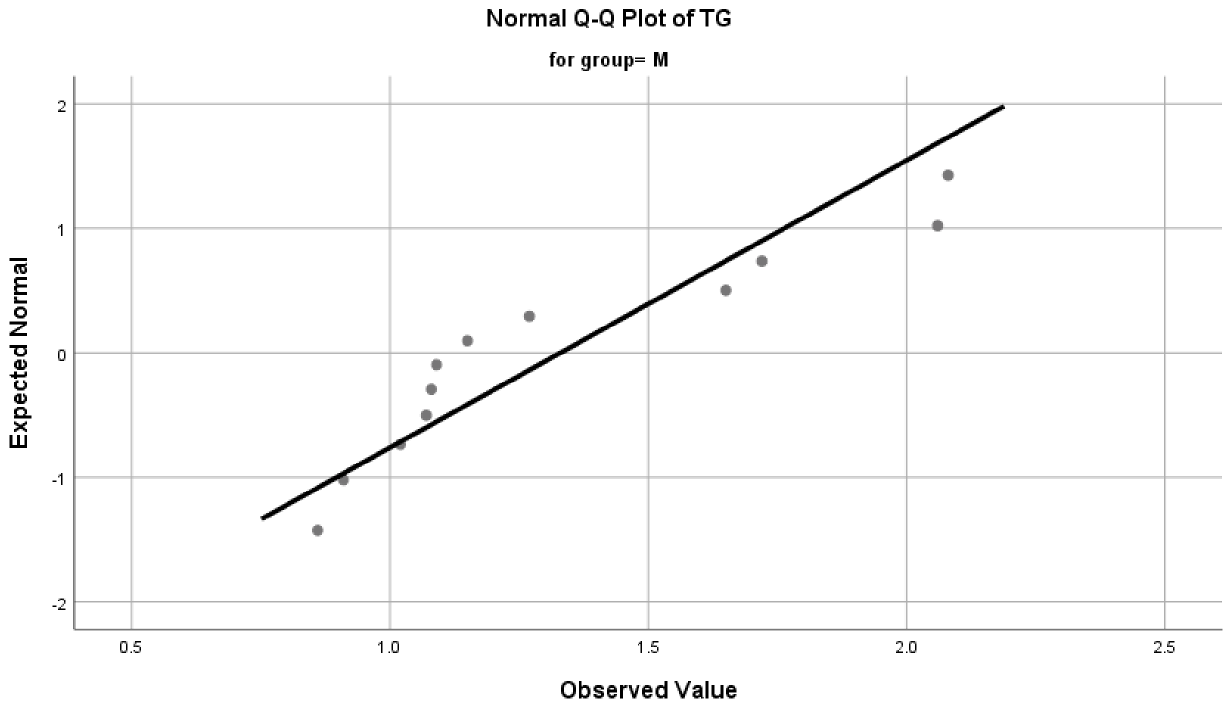
Normal Q-Q Plot of TG
for group= M
Expected Normal
2
1
0
-1
-2
0.5
1.0
1.5
2.0
2.5
Observed Value

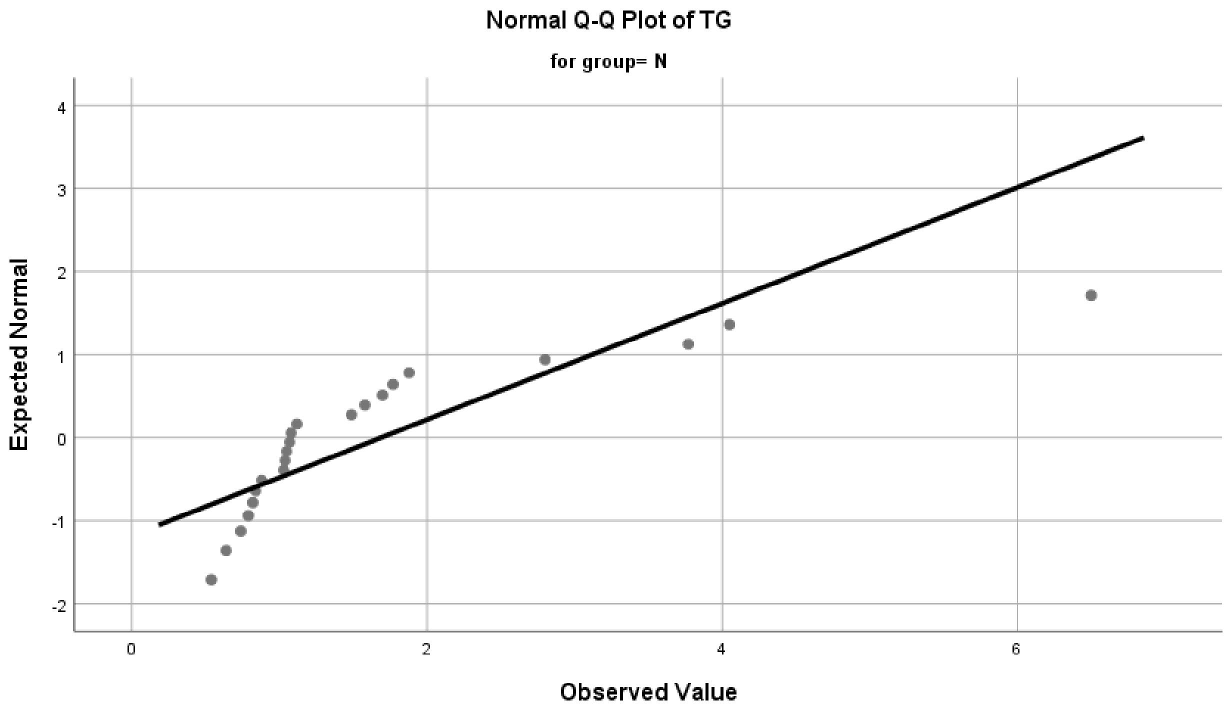

Normal Q-Q Plot of TG
for group= N
Expected Normal
Observed Value

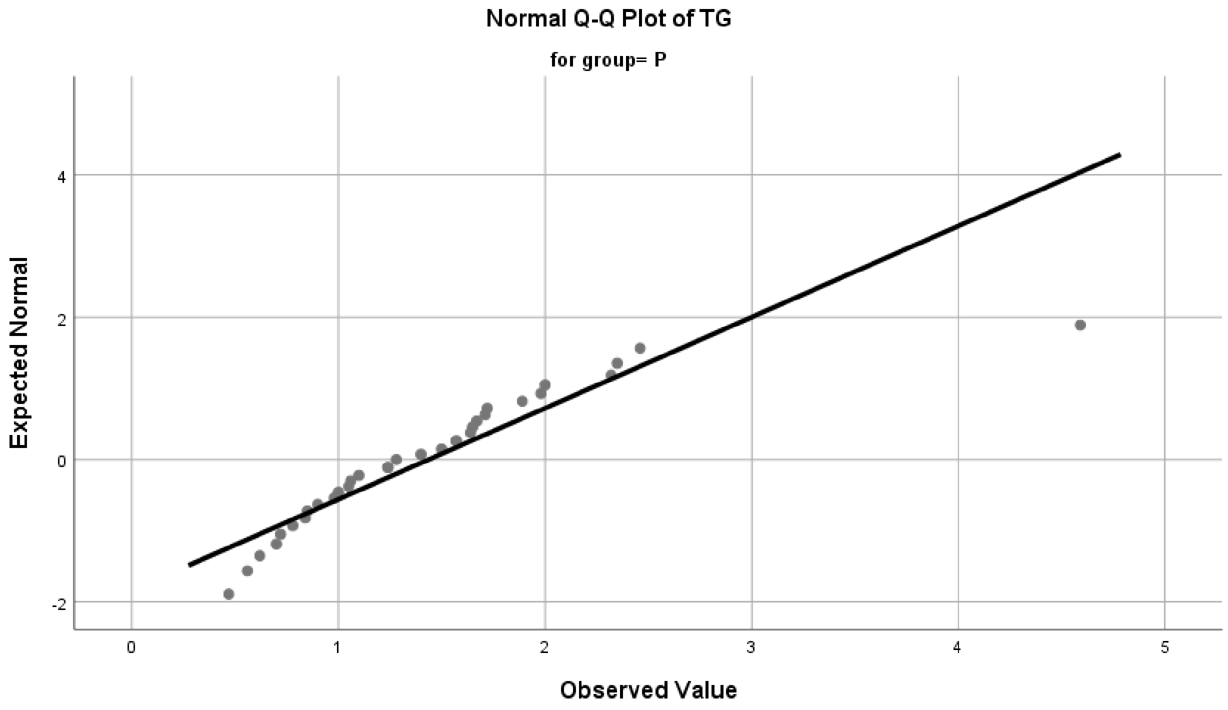

Normal Q-Q Plot of TG
for group= P
Expected Normal
Observed Value

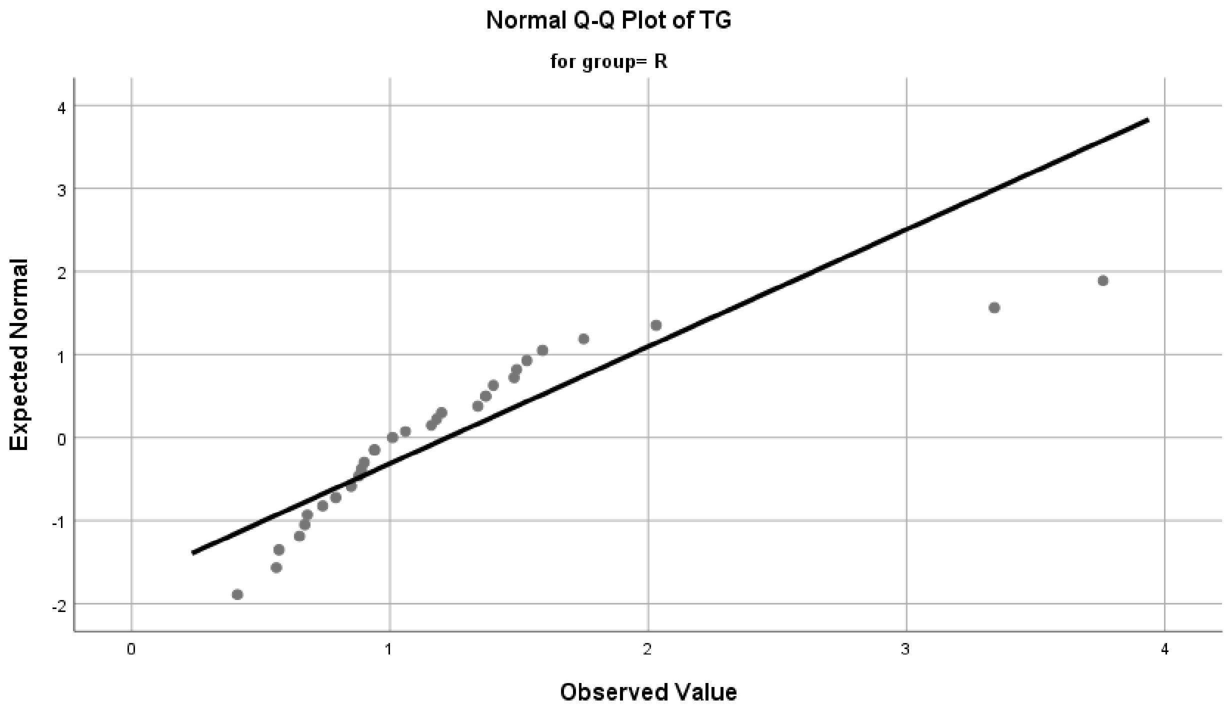
Normal Q-Q Plot of TG
for group= R
Expected Normal
Observed Value
4
3
2
1
0
-1
-2
0
1
2
3
4

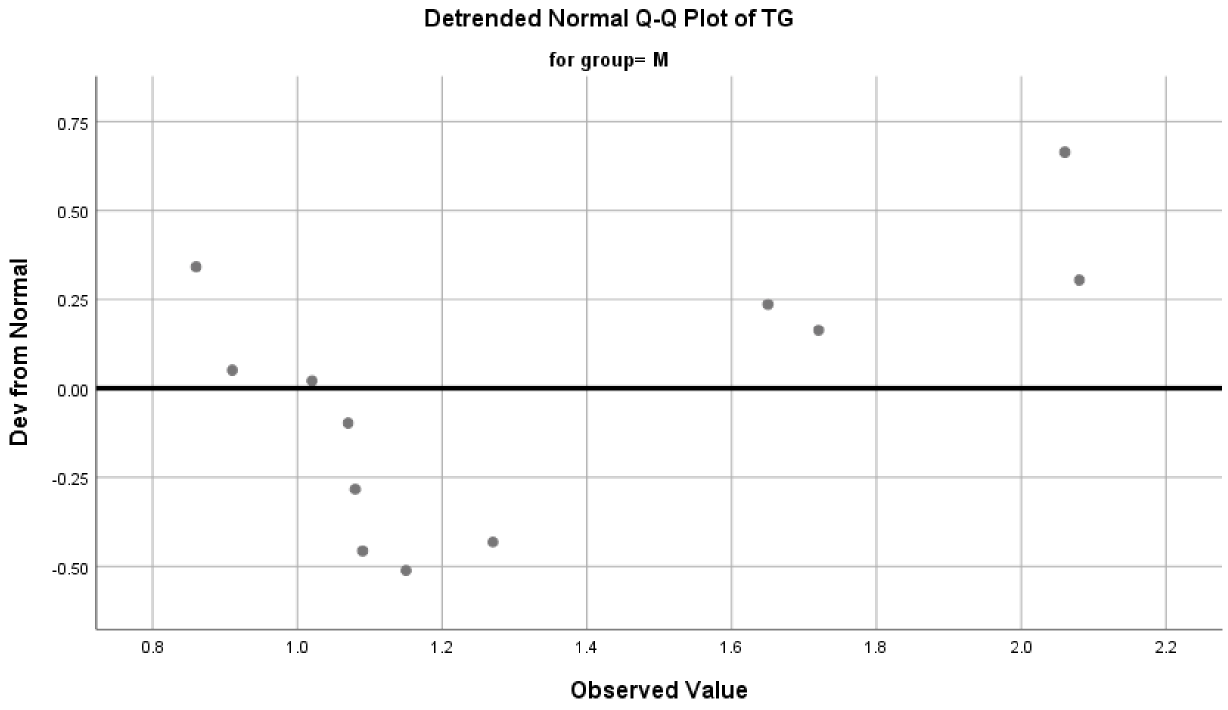
Detrended Normal Q-Q Plot of TG
for group= M
Dev from Normal
Observed Value
0.75
0.50
0.25
0.00
-0.25
-0.50
0.8
1.0
1.2
1.4
1.6
1.8
2.0
2.2

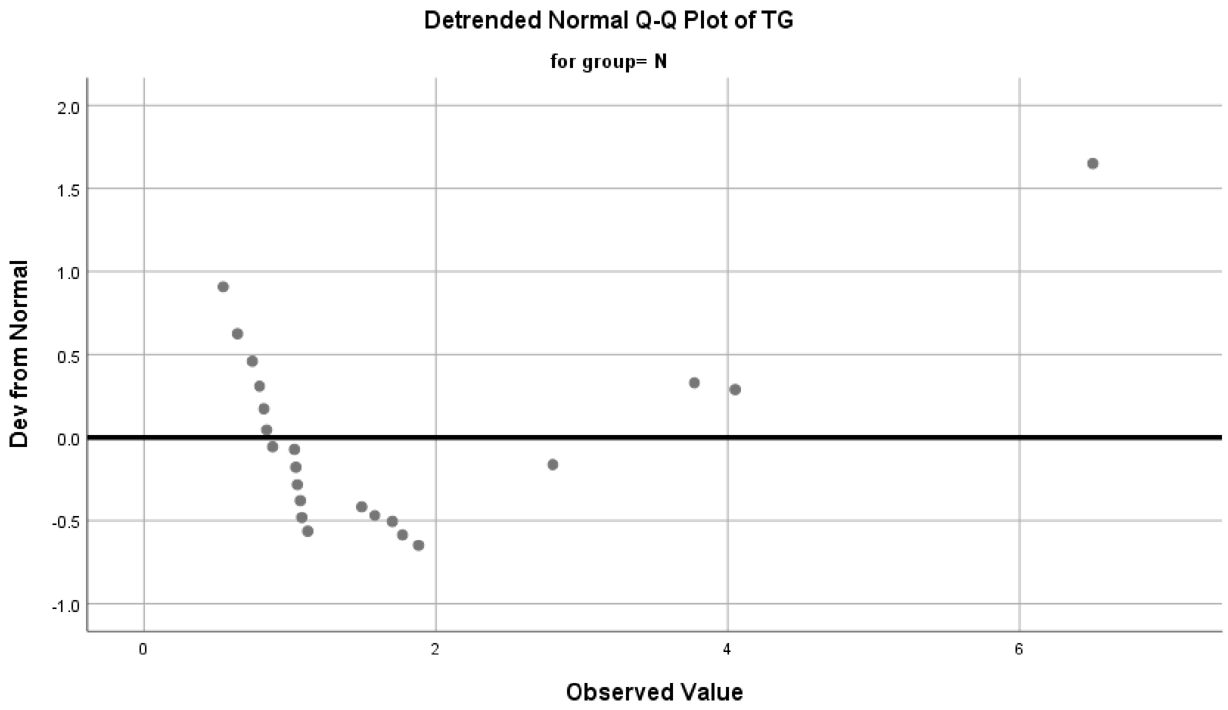
Detrended Normal Q-Q Plot of TG
for group= N
Dev from Normal
Observed Value
2.0
1.5
1.0
0.5
0.0
-0.5
-1.0
0
2
4
6

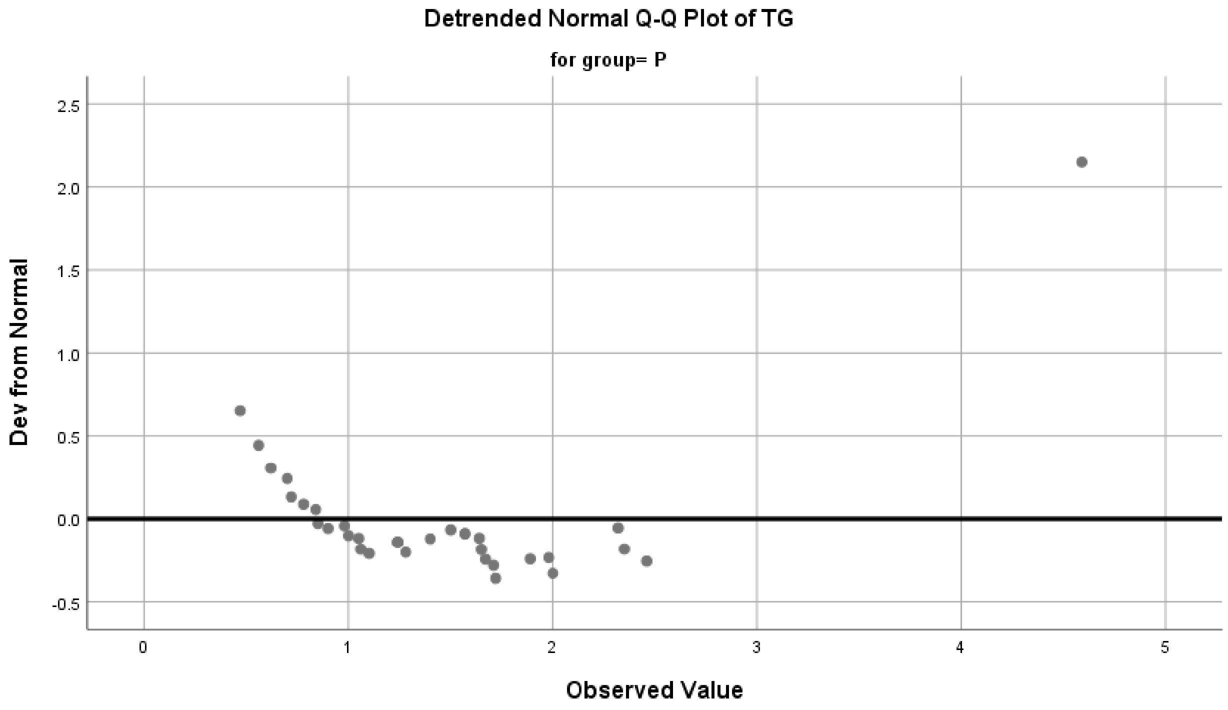
Detrended Normal Q-Q Plot of TG
for group= P
Dev from Normal
Observed Value
2.5
2.0
1.5
1.0
0.5
0.0
-0.5
0
1
2
3
4
5

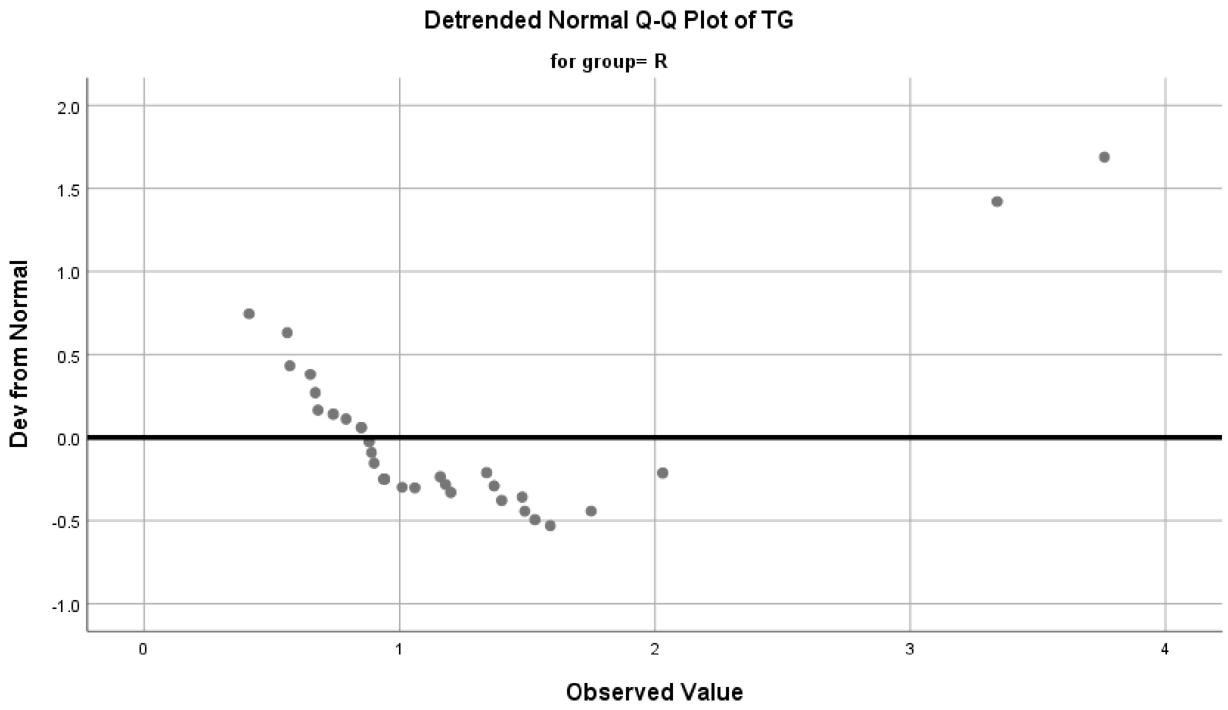
Detrended Normal Q-Q Plot of TG
for group= R
Dev from Normal
2.0
1.5
1.0
0.5
0.0
-0.5
-1.0
0
1
2
3
4
Observed Value

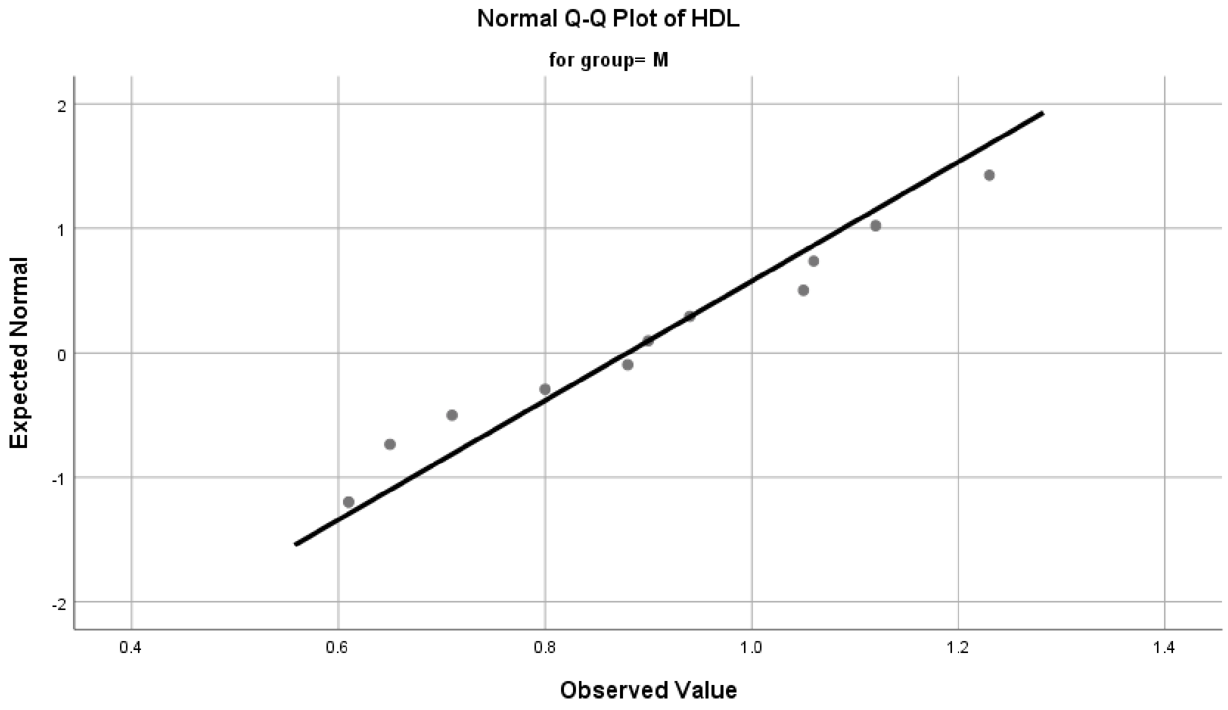
Normal Q-Q Plot of HDL
for group= M
Expected Normal
2
1
0
-1
-2
0.4
0.6
0.8
1.0
1.2
1.4
Observed Value

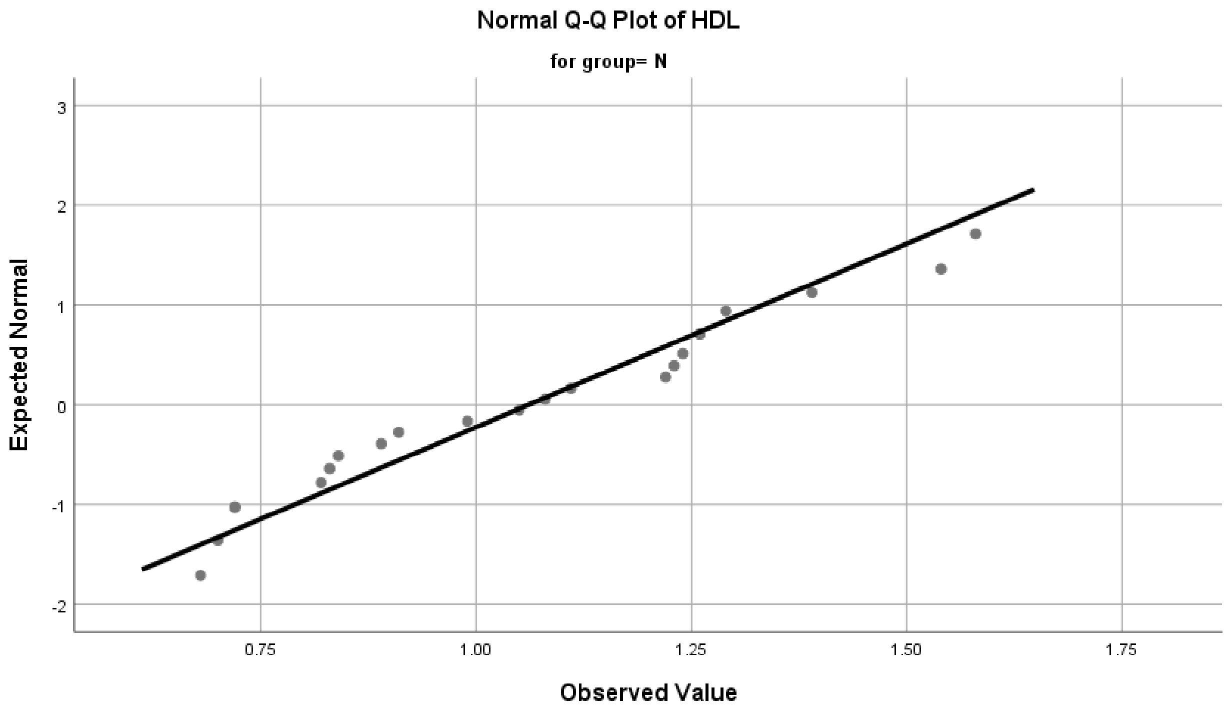
Normal Q-Q Plot of HDL
for group= N
Expected Normal
Observed Value
3
2
1
0
-1
-2
0.75
1.00
1.25
1.50
1.75

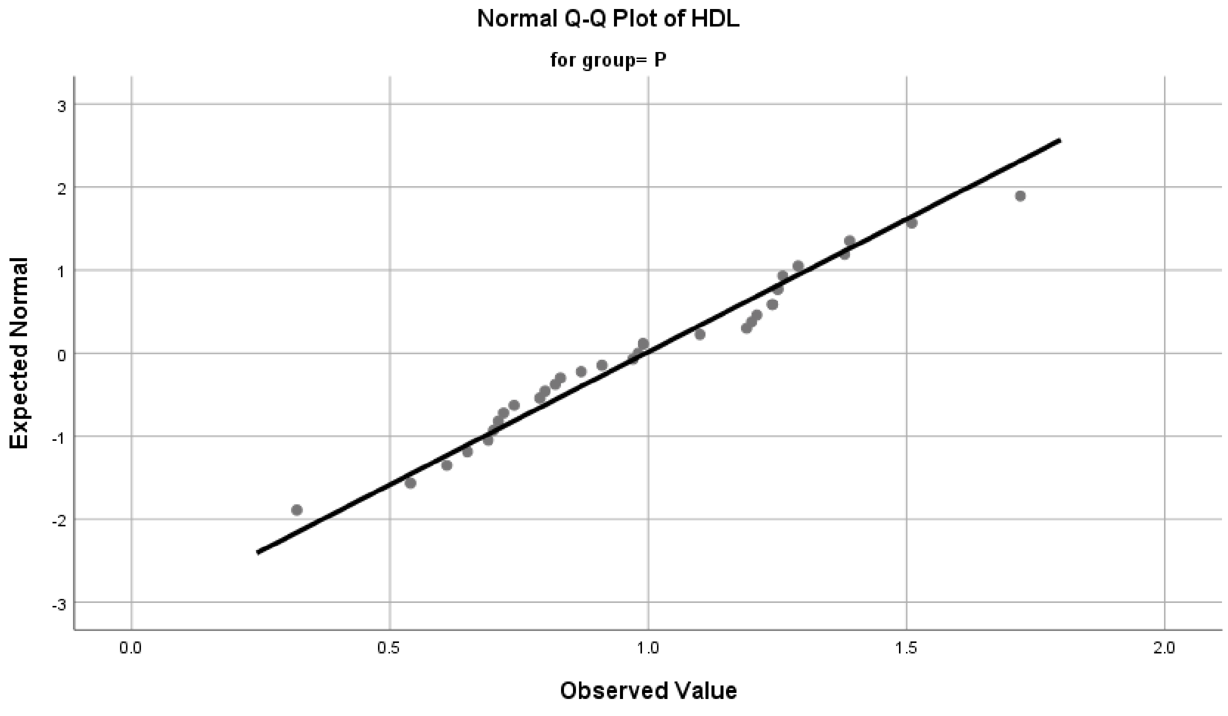
Normal Q-Q Plot of HDL
for group= P
Expected Normal
Observed Value
3
2
1
0
-1
-2
-3
0.0
0.5
1.0
1.5
2.0

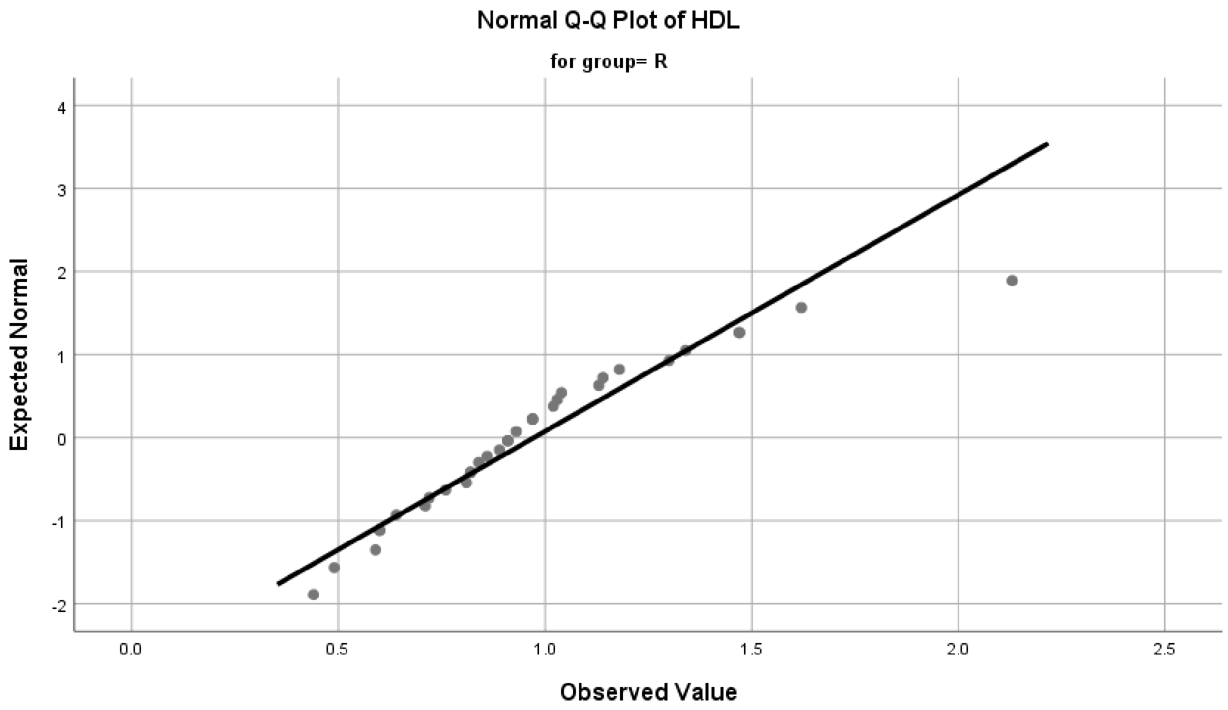
Normal Q-Q Plot of HDL
for group= R
Expected Normal
Observed Value
4
3
2
1
0
-1
-2
0.0
0.5
1.0
1.5
2.0
2.5

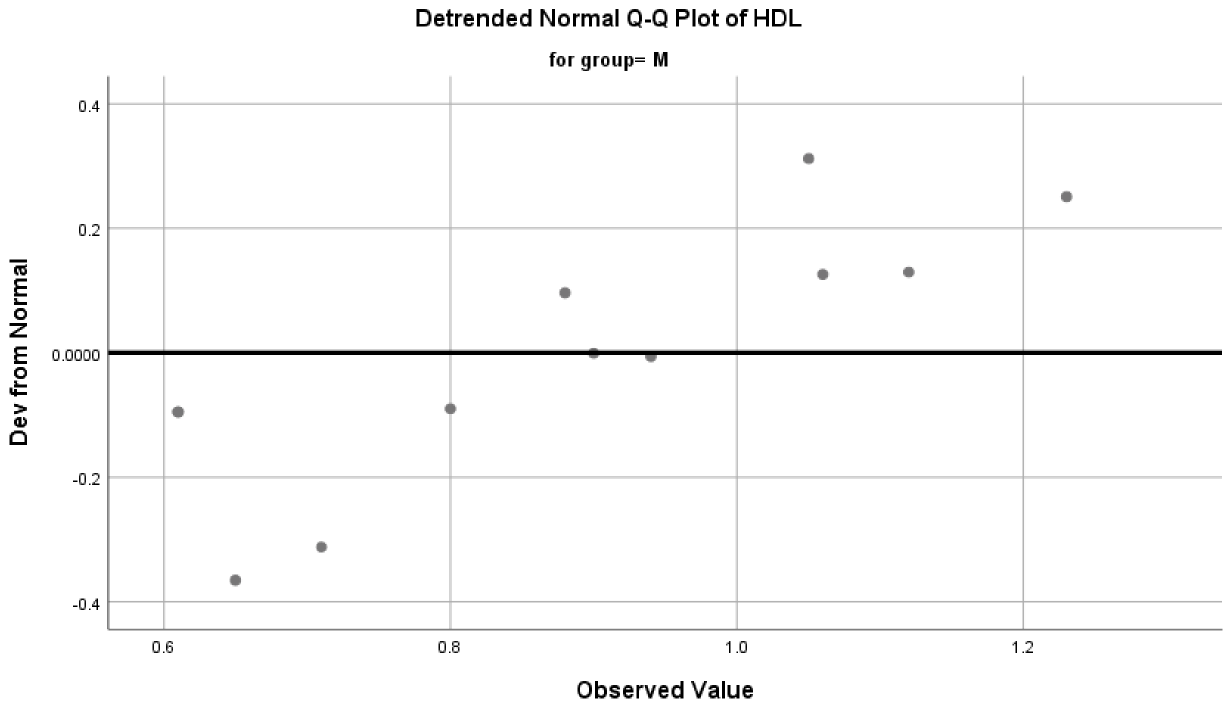
Detrended Normal Q-Q Plot of HDL
for group= M
Dev from Normal
Observed Value
0.4
0.2
0.0000
-0.2
-0.4
0.6
0.8
1.0
1.2

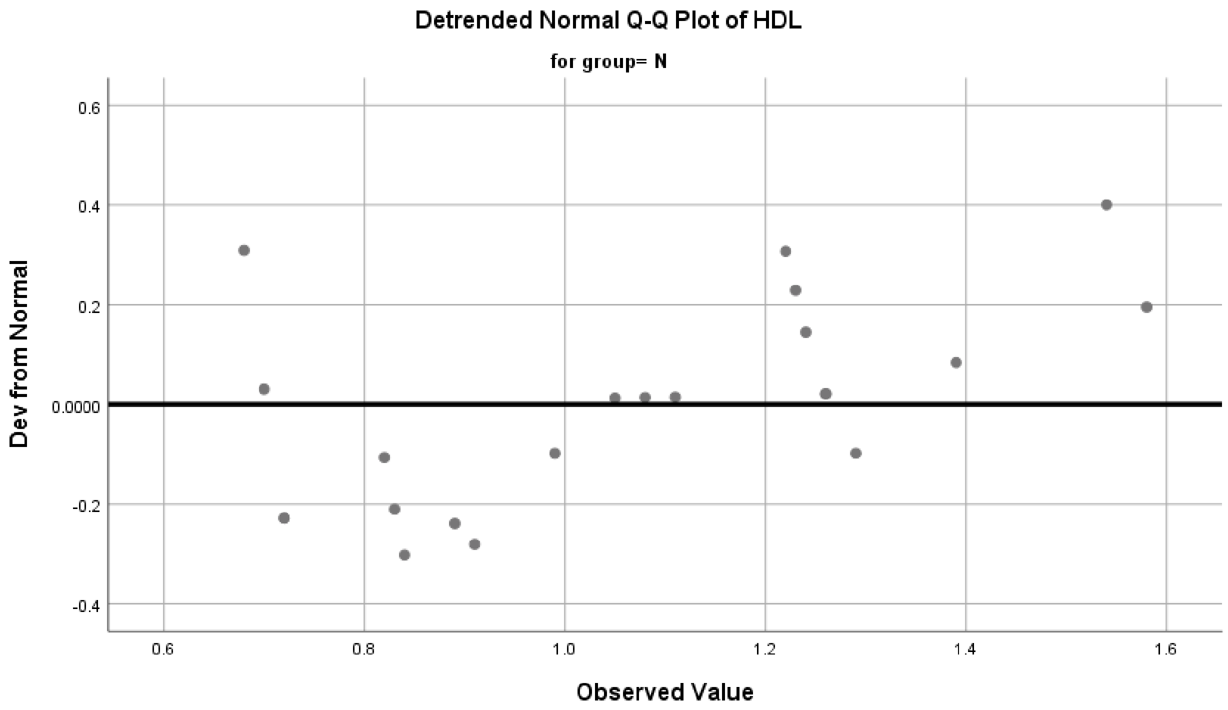
Detrended Normal Q-Q Plot of HDL
for group= N
Dev from Normal
0.6
0.4
0.2
0.0000
-0.2
-0.4
0.6
0.8
1.0
1.2
1.4
1.6
Observed Value

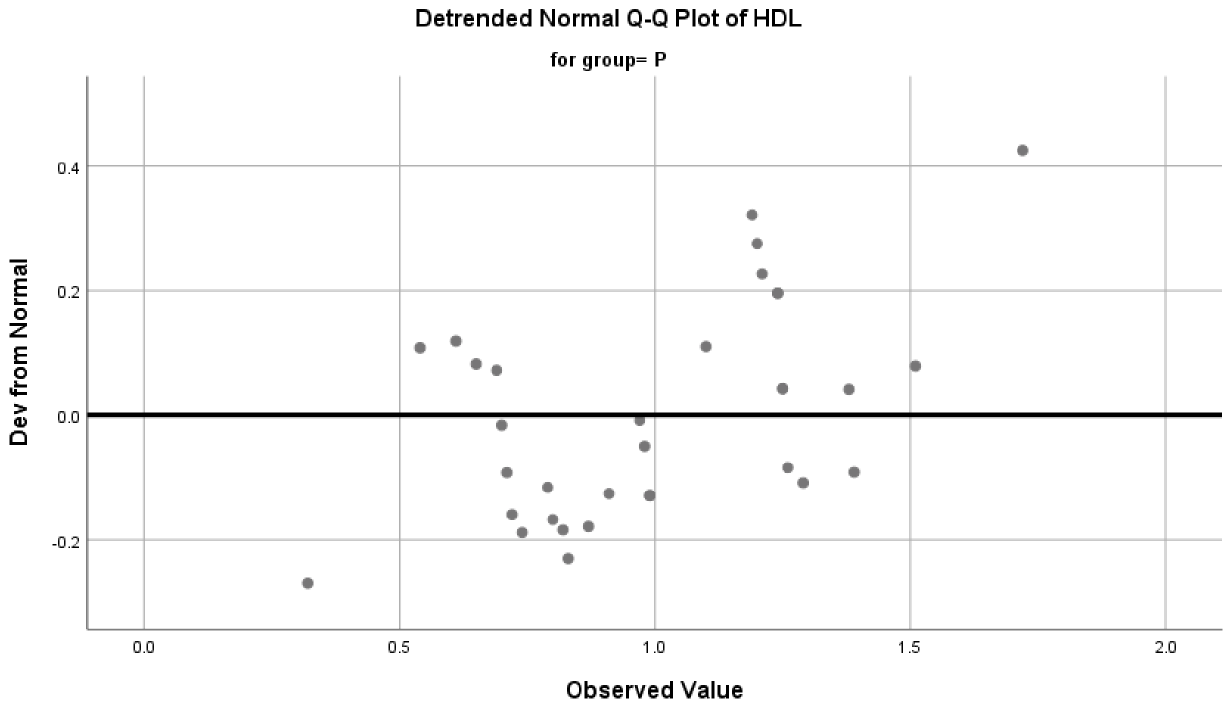
Detrended Normal Q-Q Plot of HDL
for group= P
Dev from Normal
0.4
0.2
0.0
-0.2
0.0
0.5
1.0
1.5
2.0
Observed Value

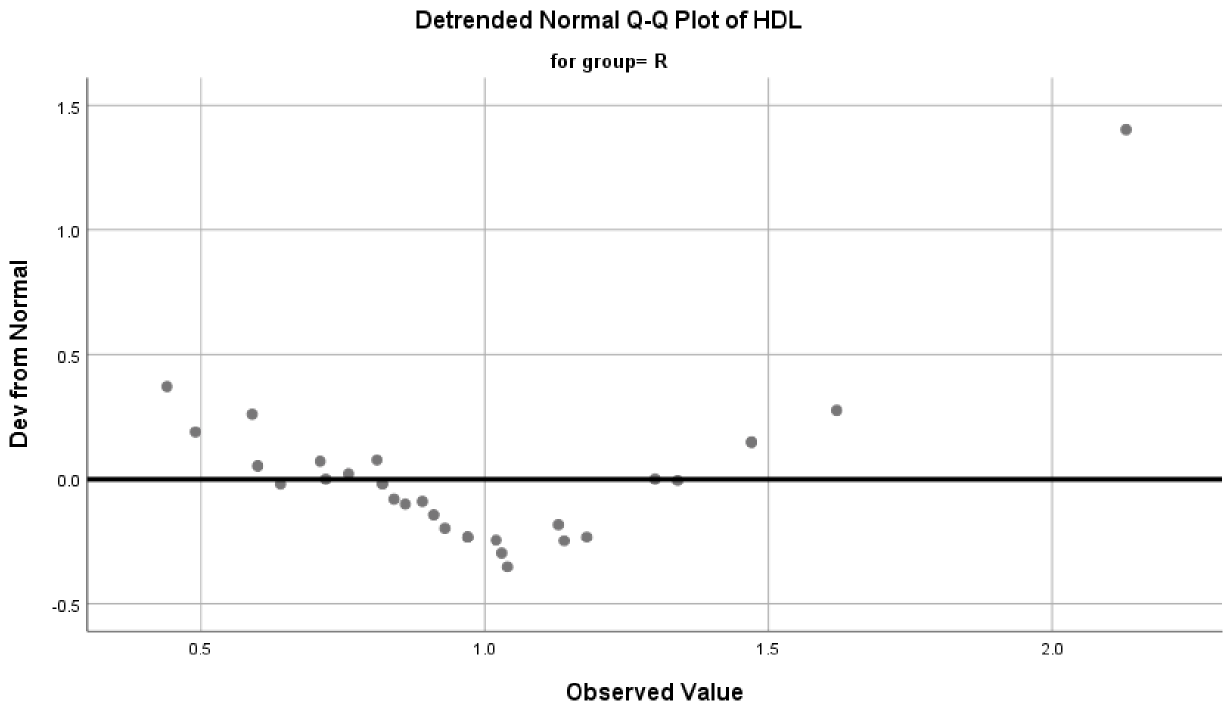
Detrended Normal Q-Q Plot of HDL
for group= R
Dev from Normal
Observed Value
1.5
1.0
0.5
0.0
-0.5
0.5
1.0
1.5
2.0

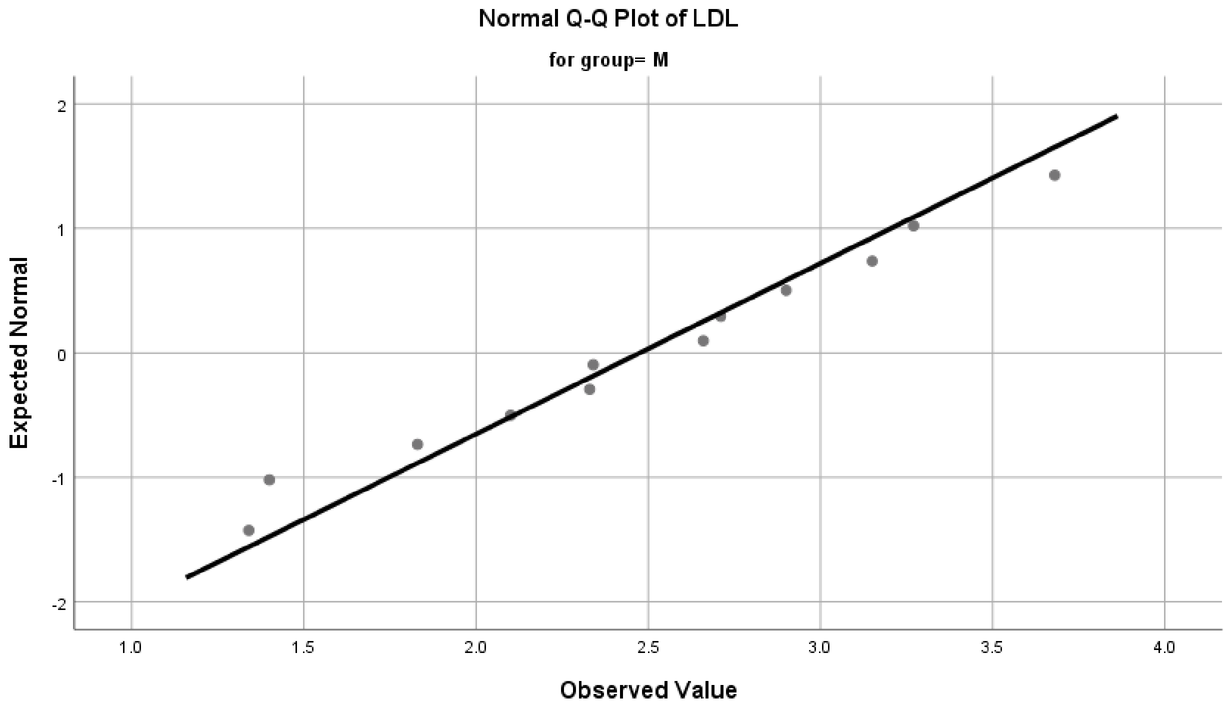
Normal Q-Q Plot of LDL
for group= M
Expected Normal
Observed Value
2
1
0
-1
-2
1.0
1.5
2.0
2.5
3.0
3.5
4.0

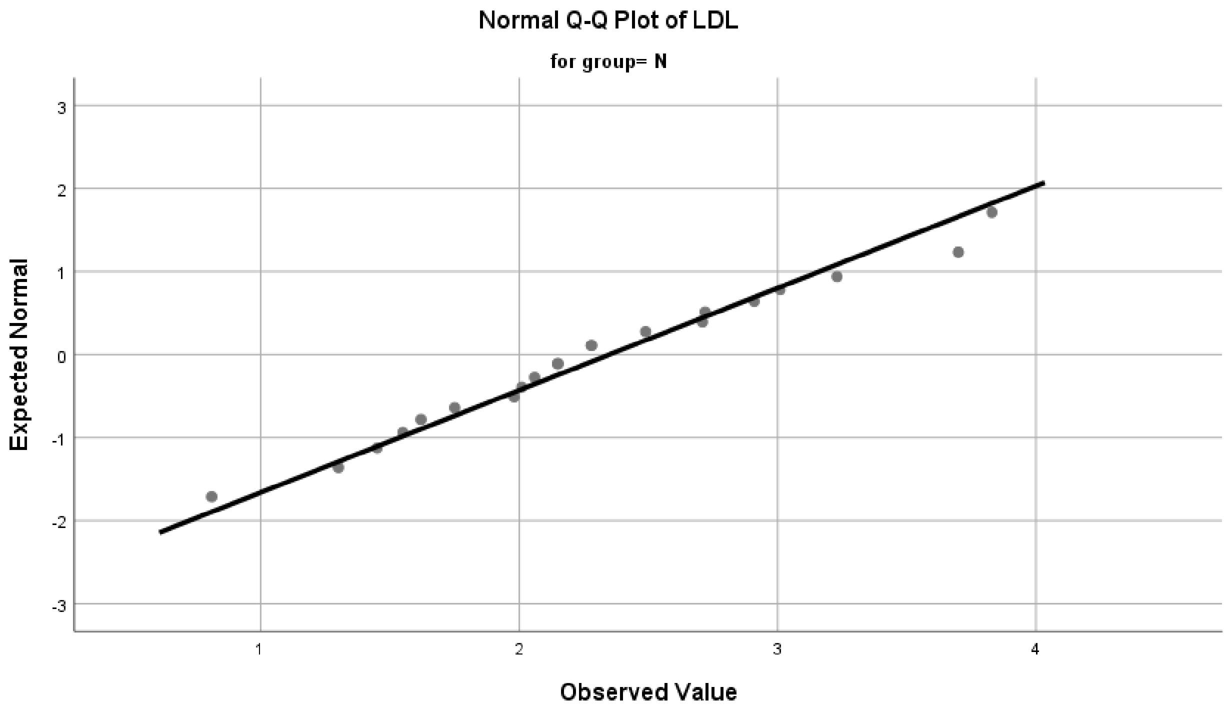
Normal Q-Q Plot of LDL
for group= N
Expected Normal
Observed Value
3
2
1
0
-1
-2
-3
1
2
3
4

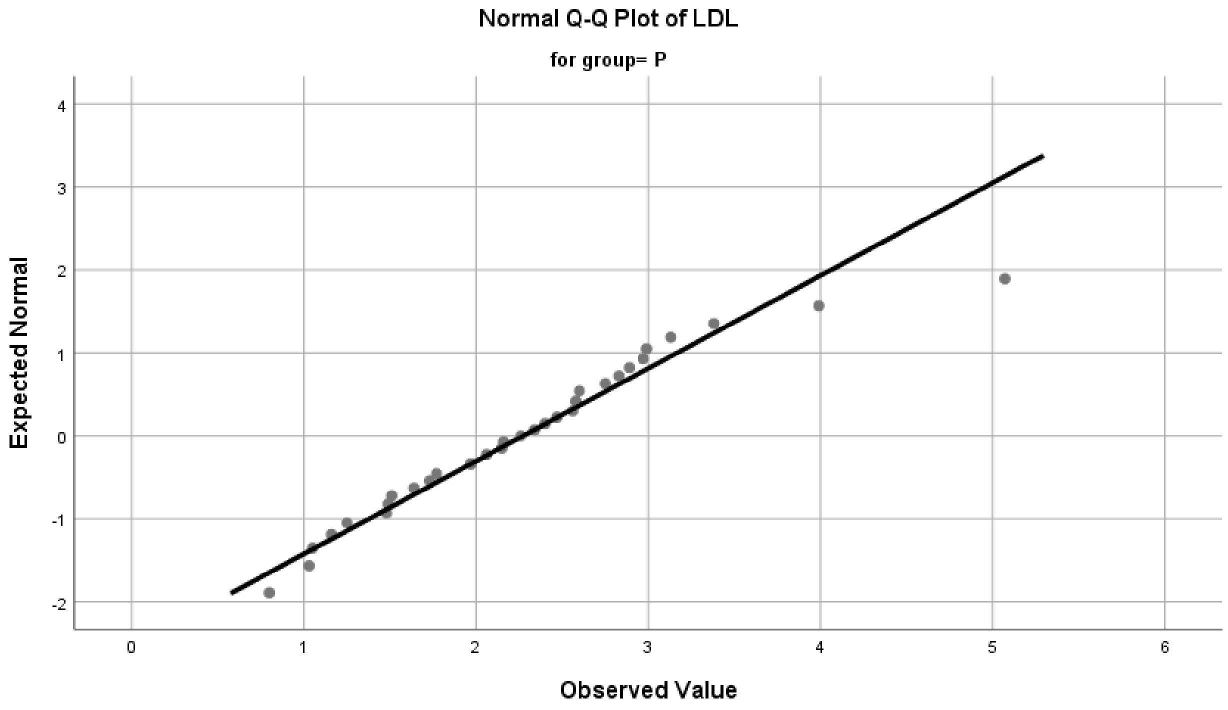
Normal Q-Q Plot of LDL
for group= P
Expected Normal
Observed Value
4
3
2
1
0
-1
-2
0
1
2
3
4
5
6

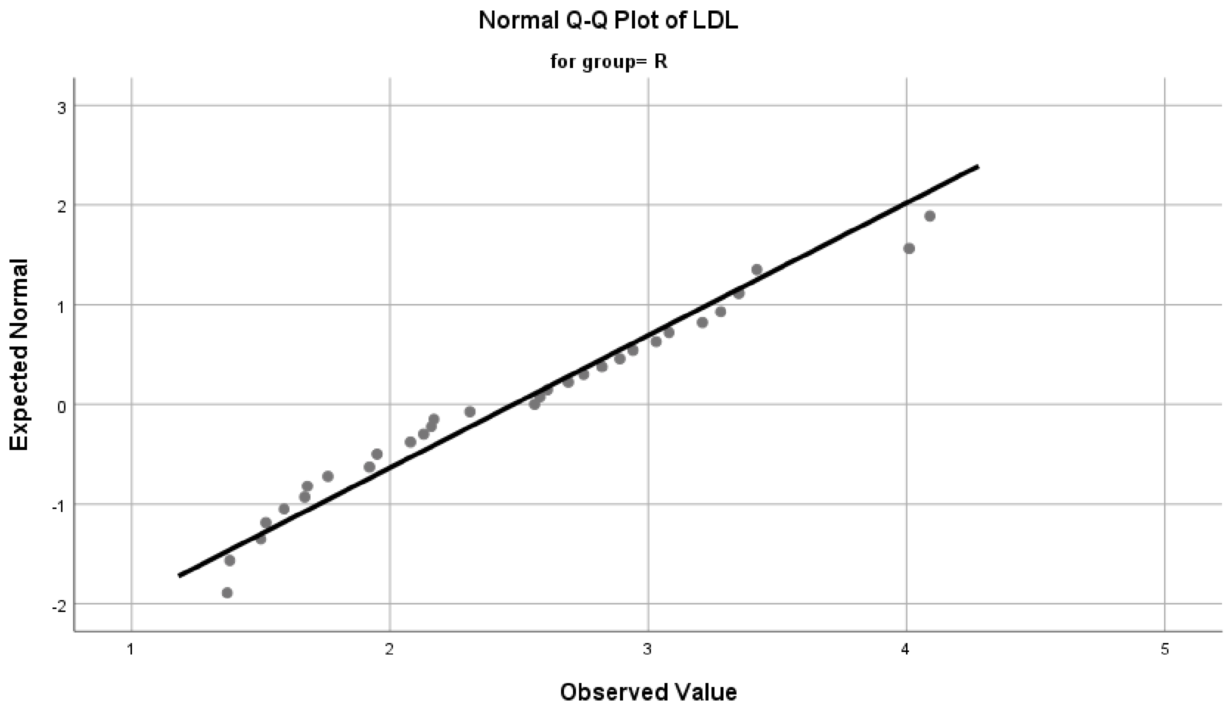

Normal Q-Q Plot of LDL
for group= R
Expected Normal
Observed Value
3
2
1
0
-1
-2
1
2
3
4
5

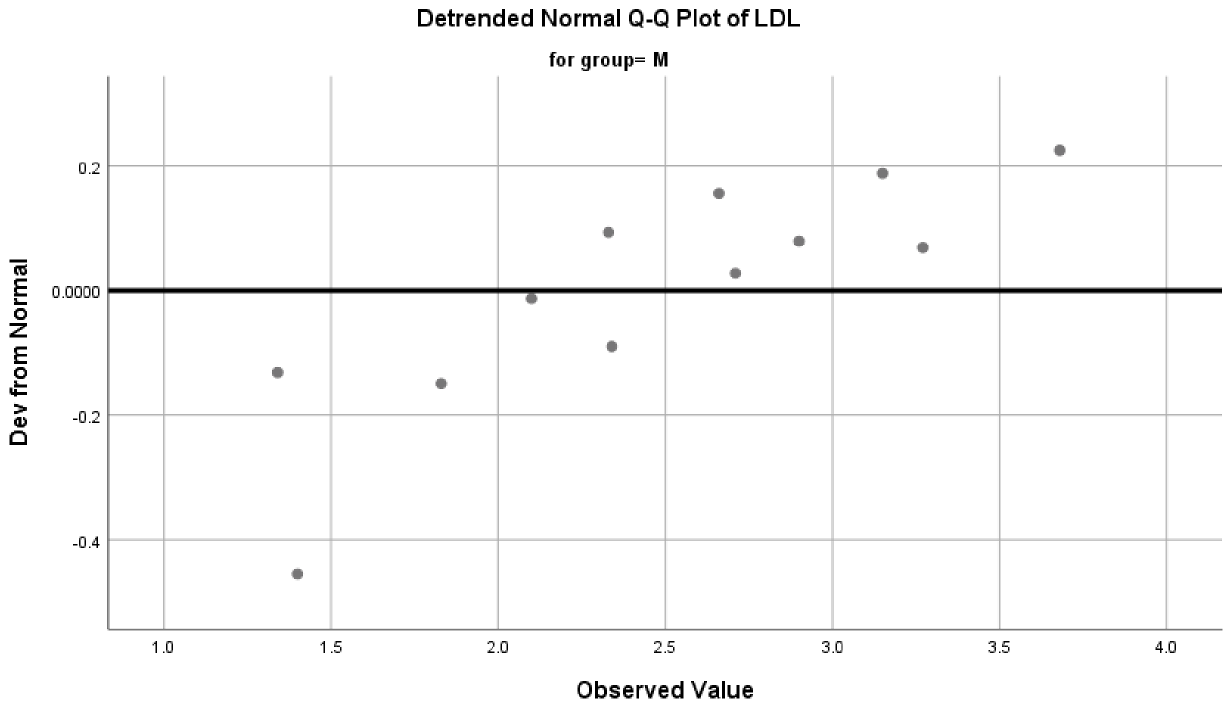

Detrended Normal Q-Q Plot of LDL
for group= M
Dev from Normal
Observed Value
0.2
0.0000
-0.2
-0.4
1.0
1.5
2.0
2.5
3.0
3.5
4.0

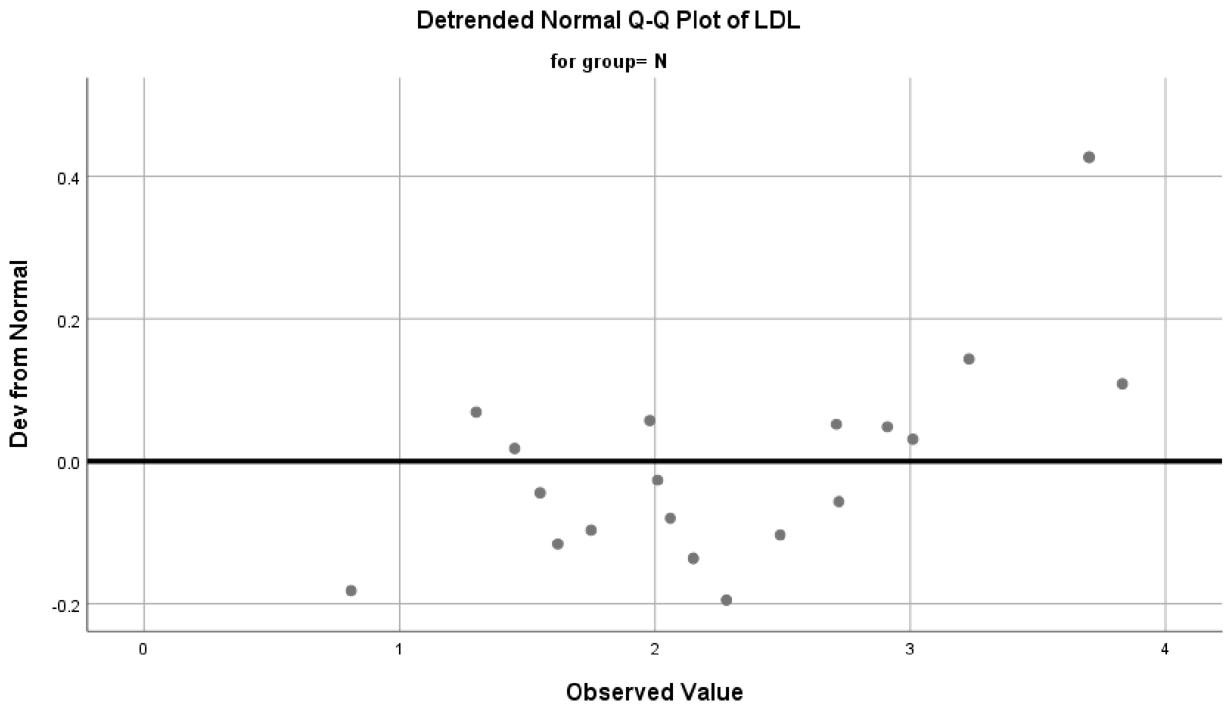

Detrended Normal Q-Q Plot of LDL
for group= N
Dev from Normal
0.4
0.2
0.0
-0.2
0
1
2
3
4
Observed Value

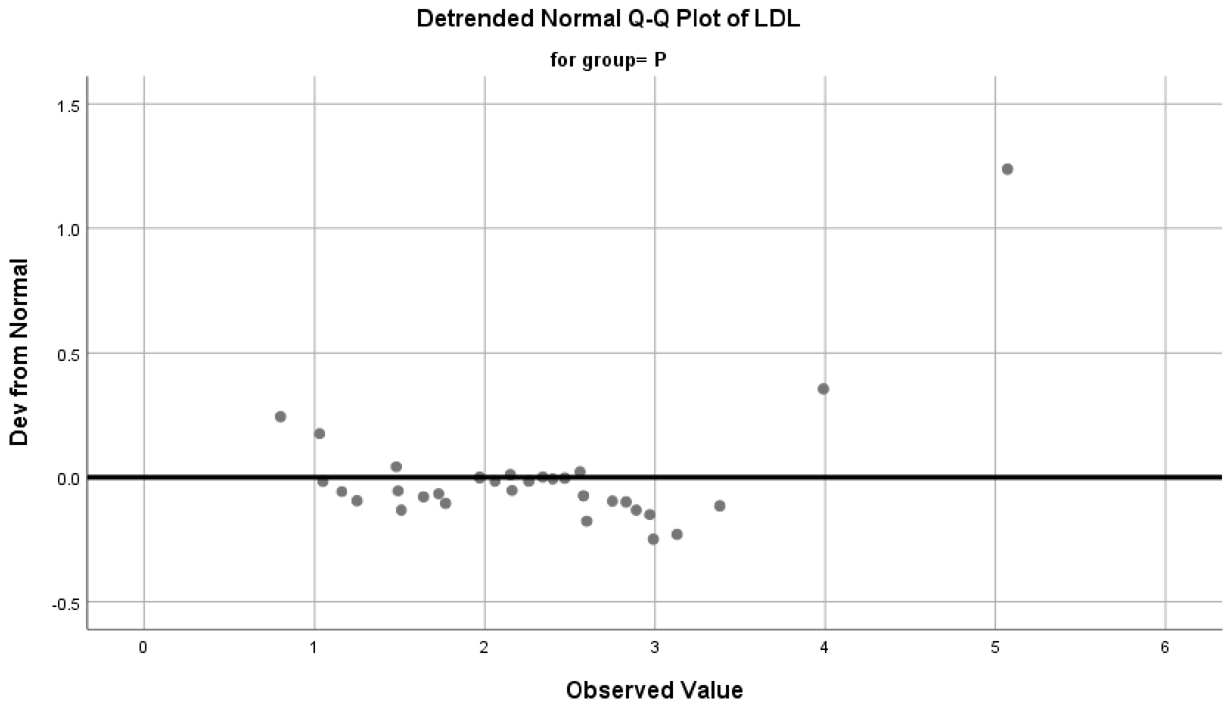

Detrended Normal Q-Q Plot of LDL
for group= P
Dev from Normal
1.5
1.0
0.5
0.0
-0.5
0
1
2
3
4
5
6
Observed Value

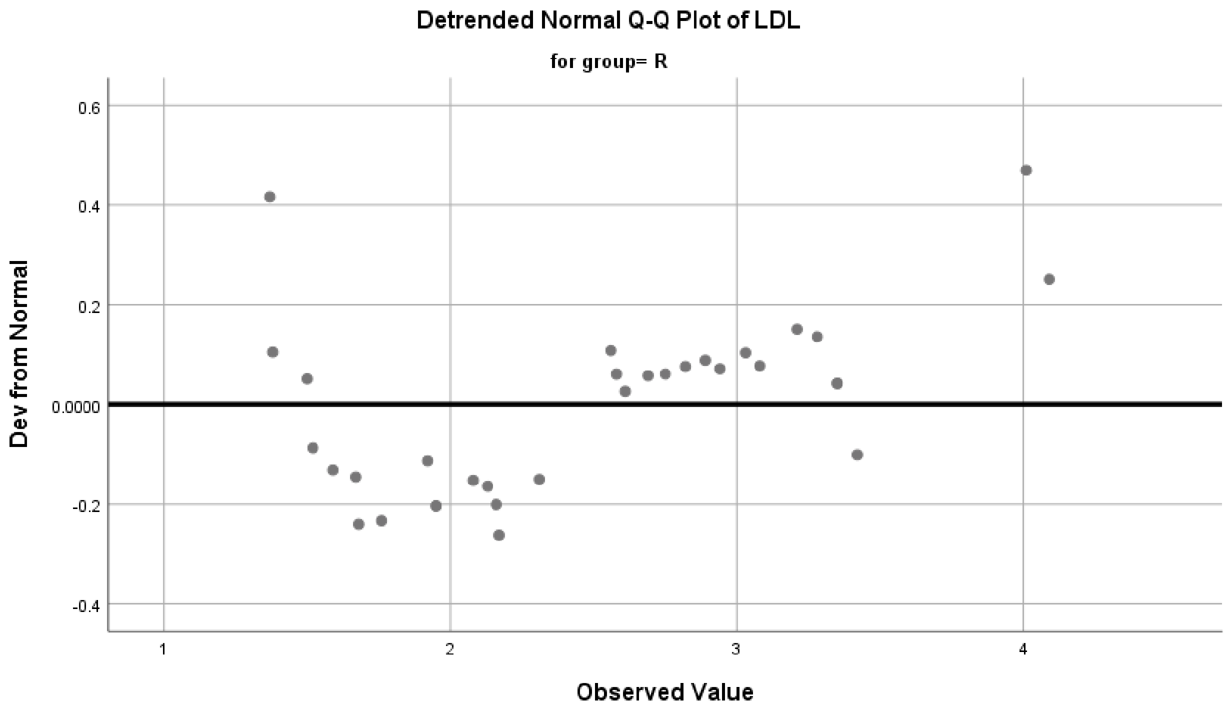
Detrended Normal Q-Q Plot of LDL
for group= R
Dev from Normal
0.6
0.4
0.2
0.0000
-0.2
-0.4
1
2
3
4
Observed Value

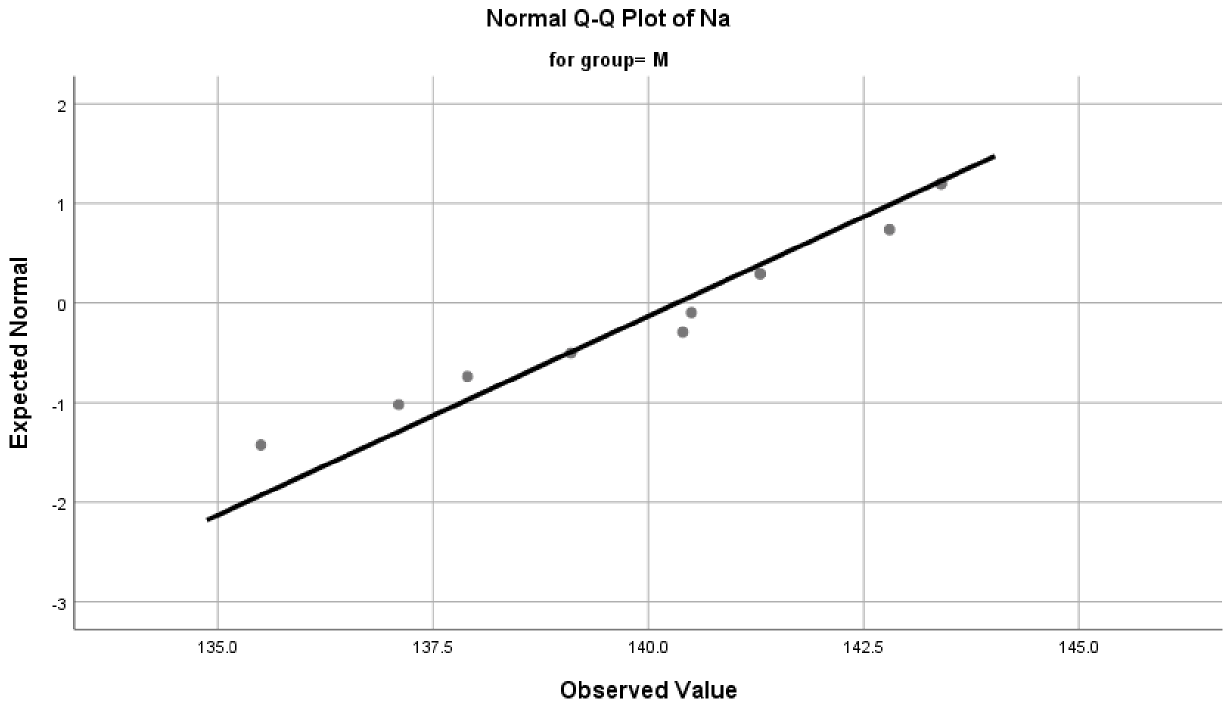
Normal Q-Q Plot of Na
for group= M
Expected Normal
2
1
0
-1
-2
-3
135.0
137.5
140.0
142.5
145.0
Observed Value

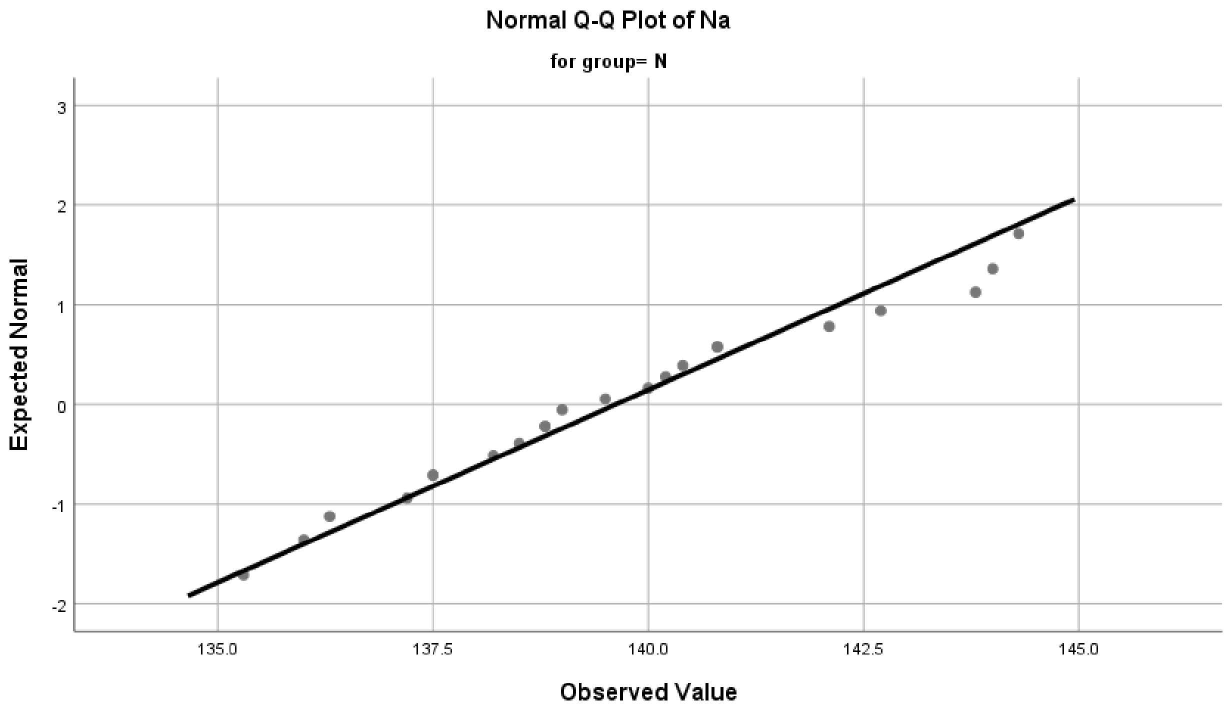

Normal Q-Q Plot of Na
for group= N
Expected Normal
Observed Value
3
2
1
0
-1
-2
135.0
137.5
140.0
142.5
145.0

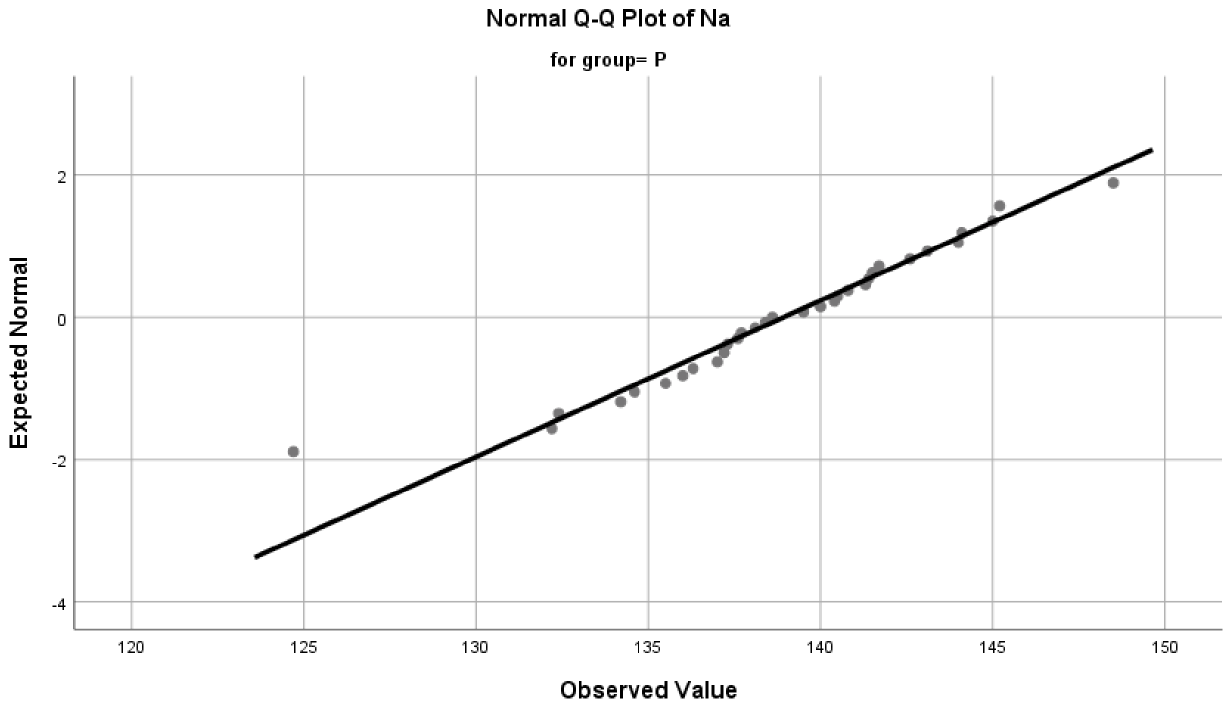

Normal Q-Q Plot of Na
for group= P
Expected Normal
Observed Value
2
0
-2
-4
120
125
130
135
140
145
150

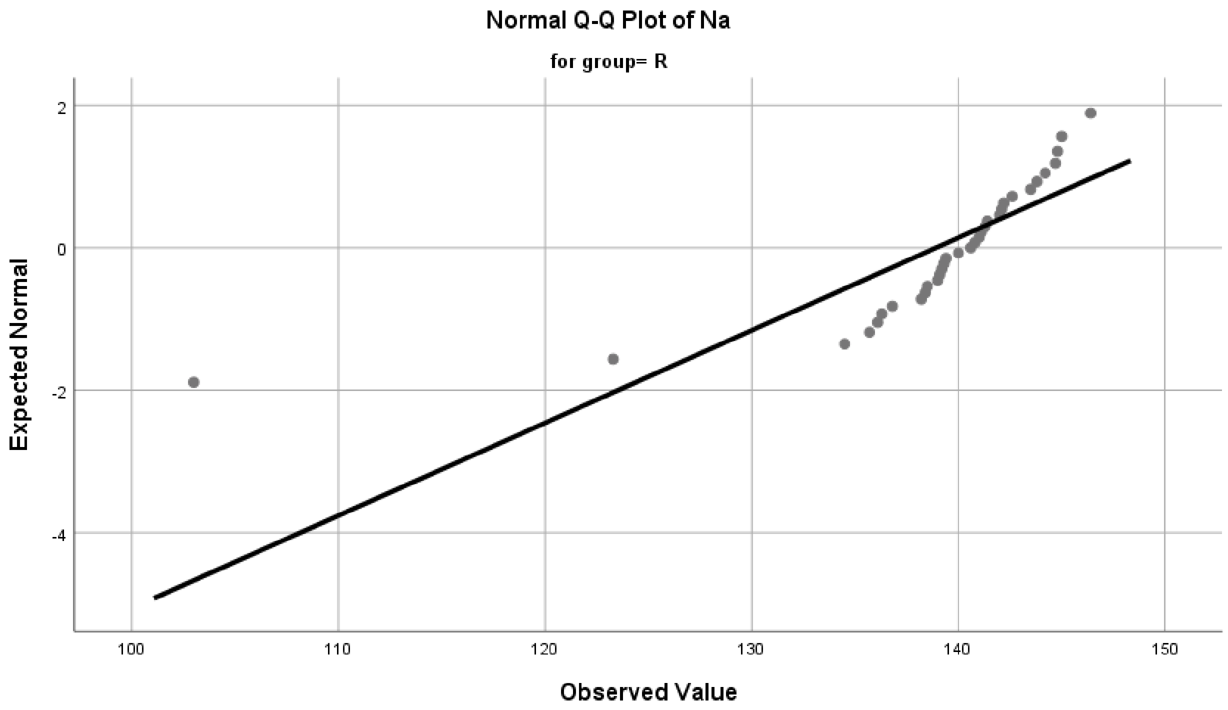
Normal Q-Q Plot of Na
for group= R
Expected Normal
2
0
-2
-4
100
110
120
130
140
150
Observed Value

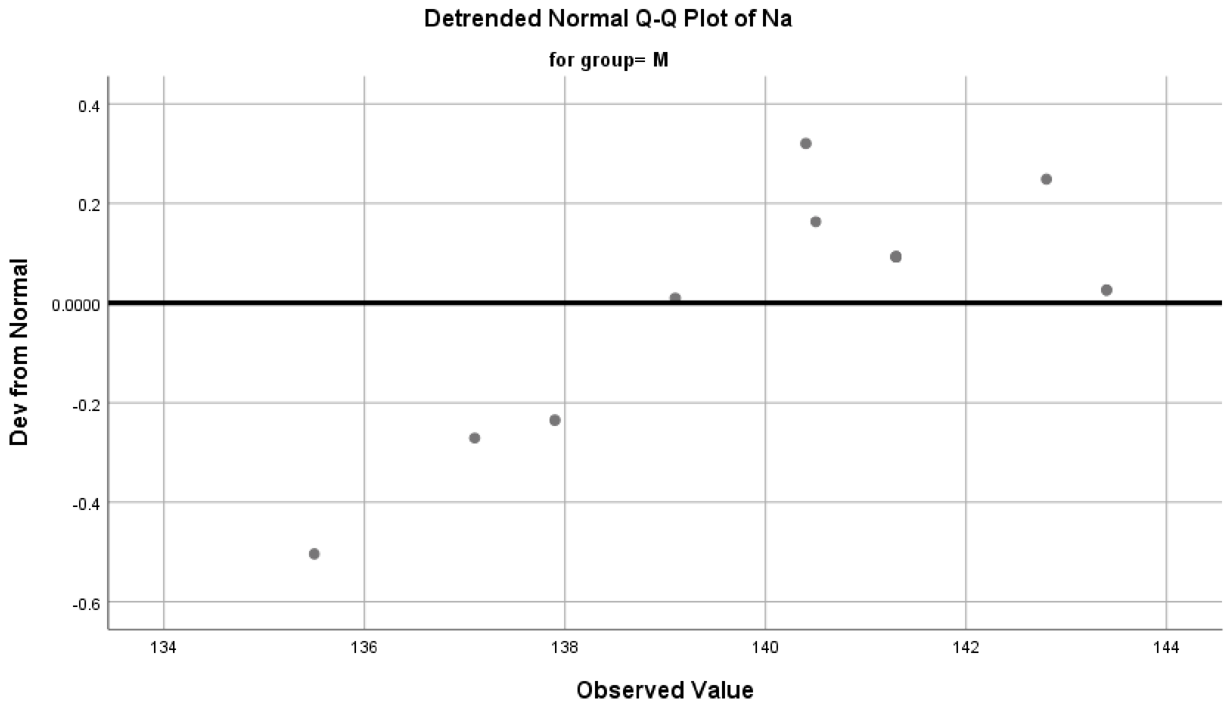
Detrended Normal Q-Q Plot of Na
for group= M
Dev from Normal
0.4
0.2
0.0000
-0.2
-0.4
-0.6
134
136
138
140
142
144
Observed Value

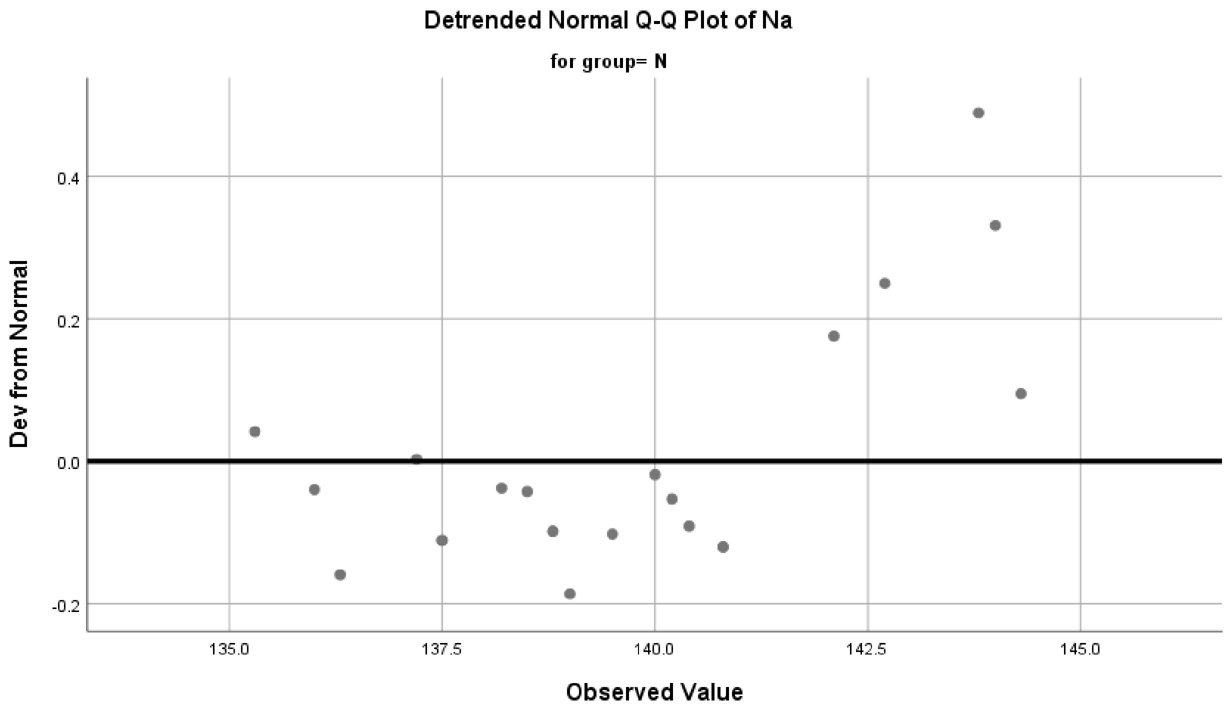
Detrended Normal Q-Q Plot of Na
for group= N
Dev from Normal
Observed Value
0.4
0.2
0.0
-0.2
135.0
137.5
140.0
142.5
145.0

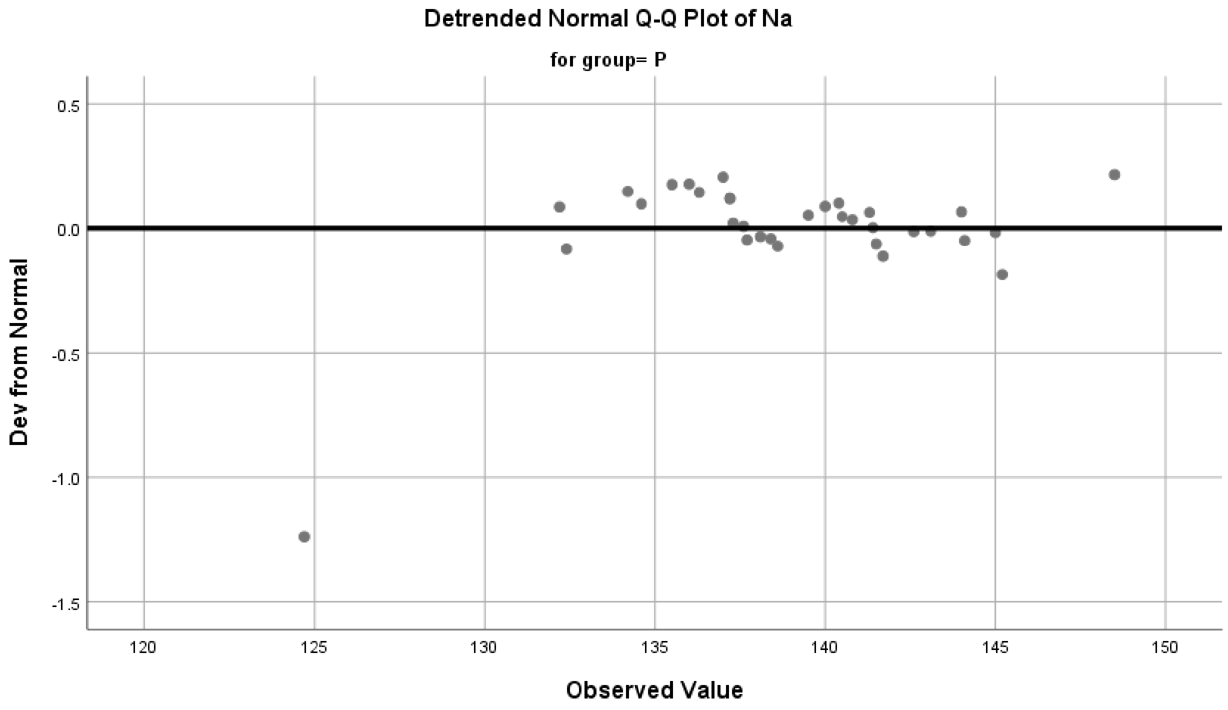
Detrended Normal Q-Q Plot of Na
for group= P
Dev from Normal
Observed Value
0.5
0.0
-0.5
-1.0
-1.5
120
125
130
135
140
145
150

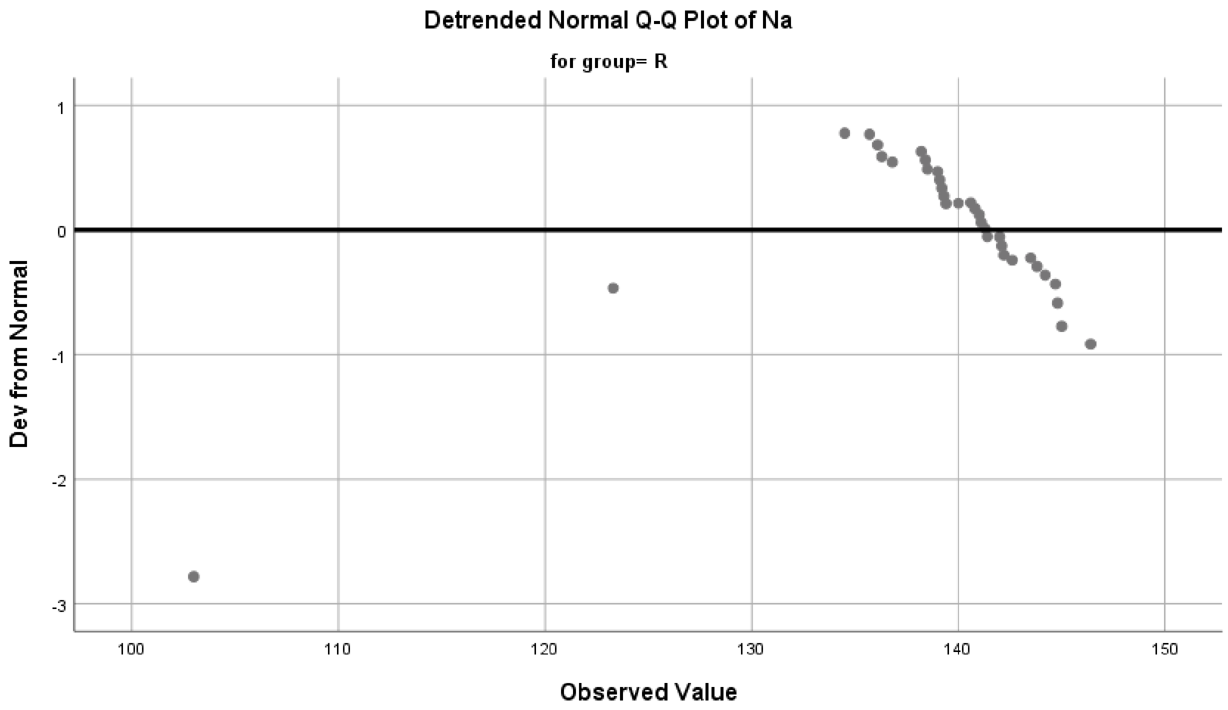
Detrended Normal Q-Q Plot of Na
for group= R
Dev from Normal
1
0
-1
-2
-3
100
110
120
130
140
150
Observed Value
