# Supplementary material for: The diagnostic value of peroxisome proliferator-activated receptor-γ coactivator-1α in identifying different chronic heart failure phenotypes
Source: Front Cardiovasc Med. 2022 Sep 6;9:973705. doi: 10.3389/fcvm.2022.973705 (PMC9485562; doi:10.3389/fcvm.2022.973705)
Supplement: Supplementary file 2 [file Image_1.pdf]

Supplementary Figure S1. The correlation analysis between PGC1α, blood glucose, and NT-proBNP.

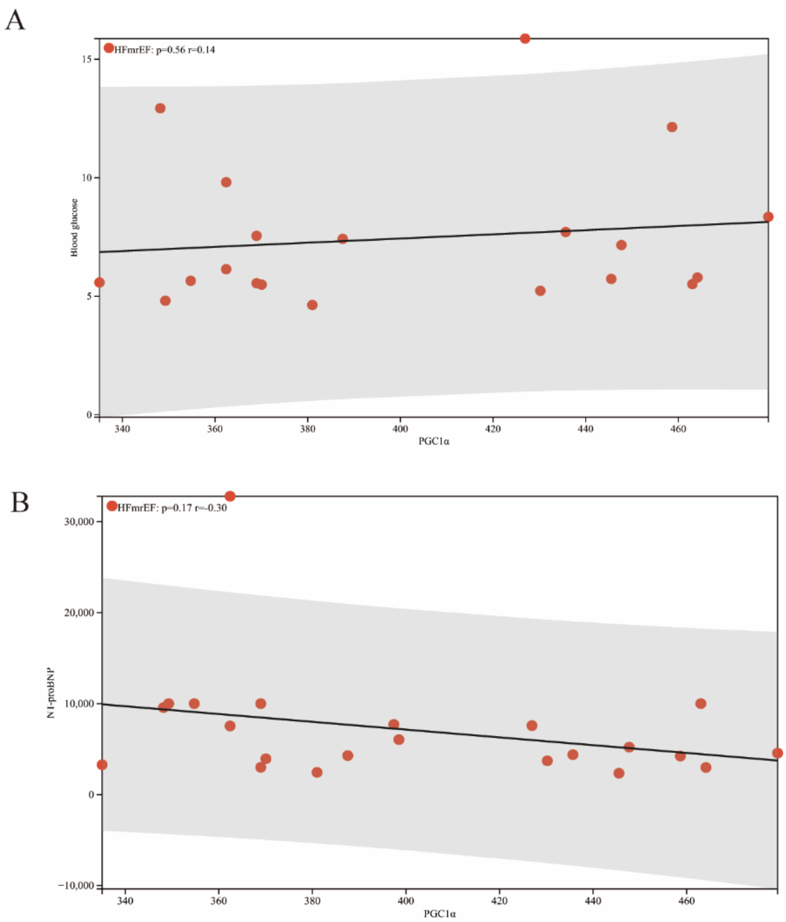

(A). The correlation analysis between PGC1α and blood glucose in the HFmrEF group. (B). The correlation analysis between PGC1α and NT-proBNP in the HFmrEF group.
